# Combining measurements to estimate properties and characterization extent of complex biochemical mixtures; applications to Heparan Sulfate

**DOI:** 10.1038/srep24829

**Published:** 2016-04-26

**Authors:** Joël R. Pradines, Daniela Beccati, Miroslaw Lech, Jennifer Ozug, Victor Farutin, Yongqing Huang, Nur Sibel Gunay, Ishan Capila

**Affiliations:** 1 Momenta Pharmaceuticals Inc., Research Department, Cambridge, MA 02142, USA

## Abstract

Complex mixtures of molecular species, such as glycoproteins and glycosaminoglycans, have important biological and therapeutic functions. Characterization of these mixtures with analytical chemistry measurements is an important step when developing generic drugs such as biosimilars. Recent developments have focused on analytical methods and statistical approaches to test similarity between mixtures. The question of how much uncertainty on mixture composition is reduced by combining several measurements still remains mostly unexplored. Mathematical frameworks to combine measurements, estimate mixture properties, and quantify remaining uncertainty, i.e. a characterization extent, are introduced here. Constrained optimization and mathematical modeling are applied to a set of twenty-three experimental measurements on heparan sulfate, a mixture of linear chains of disaccharides having different levels of sulfation. While this mixture has potentially over two million molecular species, mathematical modeling and the small set of measurements establish the existence of nonhomogeneity of sulfate level along chains and the presence of abundant sulfate repeats. Constrained optimization yields not only estimations of sulfate repeats and sulfate level at each position in the chains but also bounds on these levels, thereby estimating the extent of characterization of the sulfation pattern which is achieved by the set of measurements.

Complex mixtures of molecular species are common in biology; examples are antibodies^1^, glycoproteins^2^ and glycosaminoglycans^3^. Some of these mixtures have important therapeutic functions^4^,^5^. Interest in such mixtures has been increasing over recent years with the first FDA approval of a generic version of low-molecular-weight heparin^6^ and the prospect of developing generic versions of glycoproteins, called biosimilars^7^ in the US. One critical step during biosimilar development is the characterization of the mixture with analytical chemistry methods. For instance, because both activity and safety of a monoclonal antibody might change with its post-translational modifications, being able to identify and quantify these characteristics is paramount. To this end, much progress has been made in recent years with respect to analytical chemistry methods as applied to glycoproteins^8^. The resulting large number of measurements also stimulated the exploration of potential new statistical frameworks to test similarity between mixtures^9^. As features that are quantified by analytical methods are enumerated, the number of possible molecular species grows rapidly^4^. This complicates the problem of how many measurements might be needed to approximately resolve a mixture in terms of individual molecular species abundances. While a single measurement might not greatly restrict species abundances, the combination of well-selected measurements can be surprisingly restrictive when formalized in mathematical terms. Namely, it is shown here that constrained optimization applied to a set of measurements yields bounds for groups of molecular species abundances, thereby giving an estimate of the extent of characterization provided by this set. Mathematical approaches are of current interest to develop biosimilars^10^ and two main mathematical frameworks are presented here.

Many quantitative measurements on a mixture can be interpreted as weighted sums of species abundances, i.e. linear constraints on species abundances. Because relative abundances of species are between 0 and 1, a set of measurements corresponds to a bounded convex polyhedral set^11^. The first considered mathematical framework is optimization of convex functions over such set. This not only yields maximum-entropy estimates of mixture properties but also upper and lower bounds on these and thus quantifies remaining uncertainty on the mixture. The second mathematical framework is utilized to probe structural features of a mixture. Features are postulated by choosing a type of model for species abundances. After expressing experimental measurements as explicit functions of model parameters, constrained optimization is utilized to try and make the model reproduce experimental data. Proceeding by elimination over model types leads to identifying which relationships between individual species abundances are supported by experimental data and thus characterizes structural features of the mixture.

The mixture studied in this paper is Heparan Sulfate (HS). HS consists of oriented linear chains of disaccharides having different levels of sulfation. It is ubiquitously found in mammalian tissues, mediates interaction between cells and extracellular matrix and has diverse biological functions in normal and pathological conditions^12^,^13^,^14^,^15^,^16^. While HS displays significant structural diversity between tissues^17^, it is thought that sulfation during HS biosynthesis tends to generate block structures along chains^18^,^19^,^20^. Blocks imply correlation: a sulfated disaccharide is more likely to be adjacent to another sulfated rather than unsulfated disaccharide. It is also thought that average sulfate level varies between the two extremities of HS chains^21^. Such variation is referred to as nonhomogeneity, as opposed to homogeneity, two terms borrowed from the field of Markov chains. By utilizing mathematical modeling it is shown here that both nonhomogeneity and correlation must be incorporated in models of HS to reproduce experimental data. Constrained optimization then yields bounds on nonhomogeneity along chains and estimations of correlation along chains. Taken together, results show that a selected set of only twenty-three measurements provides deep insight into the structure of a complex HS mixture having potentially more than two million molecular species. Other potential applications of constrained optimization and mathematical modeling to the quantitative characterization of complex mixtures are briefly mentioned in the discussion.

## Mathematical Preamble and Experimental Measurements

Call **p** = (*p*_1_, ..., *p*_*n*_) the vector of relative abundances of all possible *n* individual molecular species in a mixture. Values of *p*_*i*_ are nonnegative and they sum to 1. One measurement *b* on the mixture yields a linear constraint: 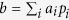. For instance, if *b* is the overall proportion of a particular glycan in a glycoprotein mixture, then *a*_*i*_ is the number of this glycan in glycoprotein form *i*. Uncertainty on measured values can be represented with inequalities and after adding slack and surplus variables to **p** this translates back into equality form. A vector **b** of *m* measurements is formalized as a set of linear constraints: **Ap** = **b** with **p** ≥ **0** and where **A** is a matrix. The polyhedral set 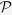 defined by these constraints might be empty due to measurement inaccuracies. Finding whether 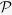 is empty or not, i.e. whether measurements are compatible with each other or not, is called a feasibility problem^22^ and it can be solved for instance via linear programming^23^,^24^. While exact determination of all *p*_*i*_ is likely to require at least *n* measurements, even a small number *m* of constraints can greatly restrict some individual abundances. Consider indeed the following linear program:


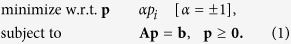


Solutions 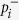 (*α* = 1) and 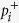 (*α* = −1) correspond to smallest and largest possible values of *p*_*i*_. Quantity 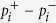 measures the remaining uncertainty on species *i*, depends not only on types of measurements (matrix **A**) but also on their values (vector **b**) and thus can be less than 1 even when *m* < *n*. Two more general constrained-optimization frameworks are considered in this paper. In the first one, the optimization variable is vector **p** of individual species abundances:


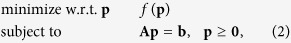


but objective function *f* is either a different linear function to estimate bounds on some mixture properties or the negative entropy to obtain maximum-entropy estimates. The second framework is utilized to investigate some structural features of the mixture by testing whether a model *q*(*θ*) of **p** can reproduce experimental data **b** or not:


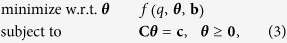


where objective function *f* is nonnegative and only zero for perfect fit. Obtaining *f* requires expressing some experimental measurements as explicit functions of model *q*(*θ*) and a methodology based on probability calculus is presented in this paper. Explicit constraints in optimization framework (3) depend on model type *q* and on some of the experimental measurements.

Modeling in optimization framework (3) is convenient for the mixture studied here, because Bovine Kidney HS (BKHS) consists of oriented linear chains of disaccharides: [non-reducing end] *d*_1_... *d*_*i*_... *d*_*n*_ [reducing end], where *d*_*i*_ stands for disaccharide at position *i*. NMR measurements showed that the average chain length in BKHS is *n* = 16 disaccharides. BKHS chains can be cleaved by enzymes called *heparinases*. Utilizing a cocktail of *heparinases* I, III and IV, BKHS was fully cleaved, resulting fragments were separated and their relative abundances quantified, at the exception of fragments containing a non-reducing end. This gave 13 building blocks which were partitioned into two groups based on their 2-*O*-sulfation status: S for 2- *O*-sulfated and U for unsulfated. Indeed, 2-*O*-sulfation determines the propensity of cleavage by *heparinase* I and by *heparinase* III. Cleavage yields of U and S by these two enzymes were estimated with additional experiments and results are summarized in Table 1(a). Components of digests by either *heparinase* I or *heparinase* III were separated by their length and relative abundances of fragments which do not include a non-reducing end were quantified. Estimated fragment length distributions are presented in Table 1(b). Detailed experimental results and protocols are provided in the supplementary material.

Even after grouping disaccharides into S/U categories, with average chain length *n* = 16 the mixture still represents at least 2^16^ = 65,536 different sequences. Yet, utilizing optimization framework (3) shows that the few measurements presented in Table 1 are enough to suggest existence of variation of sulfate level along chains (nonhomogeneity) and overrepresentation of sequences with blocks of sulfated disaccharides (correlation).

## Evidence for Nonhomogeneity and Correlation

Because disaccharide categories S and U have different propensities of cleavage by *heparinases* I and III, distributions of fragment length in *heparinase* digests reflect the pattern of sulfation along chains. To characterize this pattern, models of individual species abundances which allow for certain types of patterns are tested for their ability to reproduce *heparinase* digest data while preserving overall disaccharide composition. Pattern types are defined by two properties and their opposite. Nonhomogeneity (N) means that average sulfate level can vary with position in chains and homogeneity (H) refers to the opposite. Correlation (C) means that sulfation tends to occur in blocks along chains while independence (I) refers to lack of such correlation. There are four possible pattern types: H&I, H&C, N&I and N&C. It is shown by elimination that only combination N&C can explain experimental data. To facilitate presentation, results are shown here with models in which all BKHS chains have same length *n* = 16. In the supplementary material, results are shown to be robust to moderate changes of *n* and some results are generalized to the case of a mixture of BKHS chains having different lengths.

### Homogeneity and independence

A convenient approach to study a model of species abundances is to consider randomly drawing one chain from the mixture: the probability of drawing a sequence *s* = *d*_1_... *d*_*n*_ is the relative abundance *p*_*s*_ of this sequence. Under H&I one obtains:


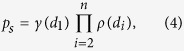


where *ρ* (*d*) is the overall proportion of disaccharide *d* between positions *i* = 2 and *n* (Table 1). Proportions *γ* of S and U at the non-reducing end (*i* = 1) do not contribute to experimental measurements but are still represented for completeness. Combining random drawing of one chain with its random cleavage at each disaccharide *d* based on *heparinase* yields *c*(*d*) (Table 1) and utilizing probability calculus gives the distribution *g*(*l*) of fragment length *l* which is expected under H&I:


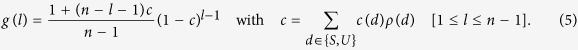


**Figure 1:**
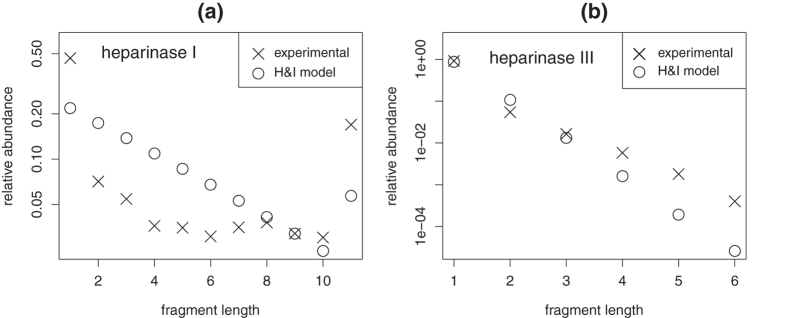
Distributions of fragment lengths *l* in *heparinase* digests (crosses) as compared to distributions expected under model H&I (dots). Relative abundances are summed for *l* ≥ 11 (*heparinase* I, (**a**)) and for *l* ≥ 6 (*heparinase* III, (**b**)).

Parameter *c* can be directly estimated from Table 1, so that optimization framework (3) is not required for H&I. Analysis of equation (5) shows that log *g* (*l*) is bound to be an almost linear function of *l*. This is illustrated with Fig. 1. Dots correspond to values computed with equation (5) for *n* = 16. Modeled log-abundances show close to linear variations with fragment length *l*. Linear behavior is however not observed with experimental data (crosses). As shown in the supplementary material, linear behavior is induced by H&I even when BKHS is modeled with a mixture of diverse chain lengths *n*. Therefore, properties H&I cannot explain the experimental data. Nonhomogeneity or correlation must be incorporated.

### Homogeneity and correlation

Correlation is now introduced in the form of a homogeneous Markov model^25^: frequency of disaccharide *b* at position *i* + 1 can depend on disaccharide *a* at position *i* but not on the value of *i*. Such a model allows for overrepresentation of species having blocks of sulfate. Model parameters are transition probabilities *P*_*ab*_ which satisfy three types of constraints:


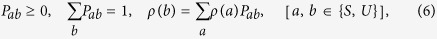


where *ρ*(*a*) is again the overall proportion of disaccharide *a*. The last constraint is known as balance equation and, combined with composition *ρ* at position *i* = 2, it guarantees that modeled abundances preserve overall disaccharide composition *ρ*. Under this H&C model, the relative abundance of species *s* = *d*_1_... *d*_*n*_ is given by


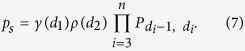


Combining random drawing of one sequence based on the above abundances to random cleavage of this chain by a *heparinase* and utilizing probability calculus gives the following expression for distribution *g*(*l*) of fragment length *l* in *heparinase* digest:


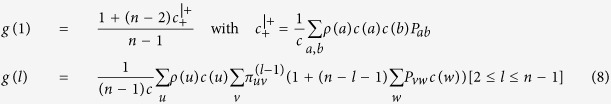


where 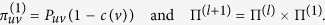


**Figure 2:**
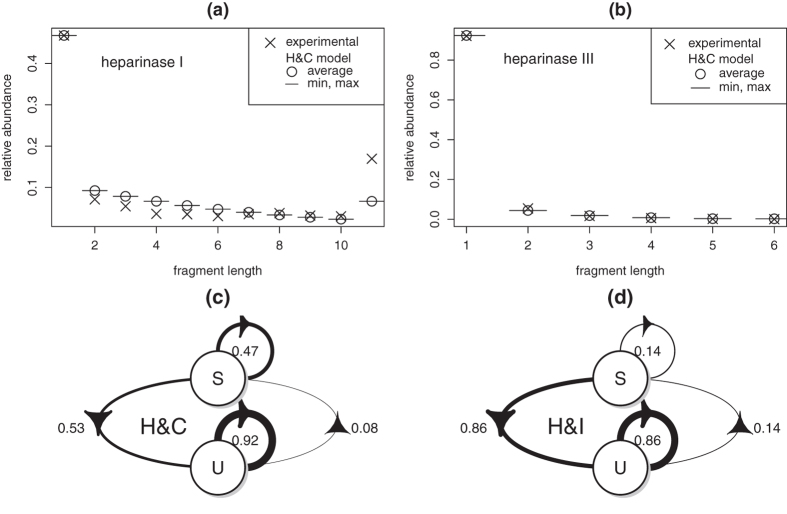
Model H&C. Distributions of fragment lengths *l* in *heparinases* I (**a**) and III (**b**) digests (crosses) as compared to distributions expected under model H&C (dots). Modeling results are summarized for 100 runs of optimization with different initial conditions. Transition probabilities of homogeneous Markov models for H&C (**c**) and H&I (**d**).

Matrices Π^(*l*)^ can be efficiently computed, so that optimizing matrix **P** to try and reproduce *heparinase* digest data can be performed in reasonable time. Call *f*_*z*_(*l*) and *g*_*z*_(*l*) the experimentally measured and modeled fragment length distributions after digestion by *heparinase z*. For each *heparinase* a maximum fragment length *l*_*z*_ is defined (11 and 6 for *heparinases* I and III) and *f*_*z*_(*l*_*z*_) and *g*_*z*_(*l*_*z*_) stand for abundances of fragment of length at least *l*_*z*_. Then, solving the optimization problem


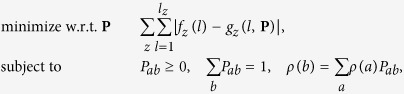


means finding a homogeneous Markov model which best fits *heparinase* digest data. Because the objective function might not be convex, simulated annealing^26^ is utilized to avoid local minima. After each perturbation of matrix **P**, constraints are enforced by projection^27^. Results are presented in panels (a) and (b) of Fig. 2 for 100 runs of optimization with different starting points. Markov models are not able to reproduce *heparinase* I digest data even for different values of BKHS chain length *n* (supplementary material). Model H&C yields a better fit than model H&I by allowing abundant repeats of sulfated disaccharides S. This is shown by panels (c) and (d) of Fig. 2 which graphically represents transition probabilities *P*_*ab*_ for optimized H&C and for H&I (*P*_*ab*_ = *ρ*(*b*)). Abundant repeats of S yield high proportion of fragments of length 1 in modeled *heparinase* I digest. This implies high abundance of long stretches of U along chains and thus relatively high abundance of long fragments in modeled *heparinase* I digest. But as shown by Fig. 2, this is not sufficient to reproduce observed abundances. Since correlation alone cannot reproduce experimental data, one concludes that nonhomogeneity is required.

### Nonhomogeneity and independence

Nonhomogeneity of sulfation along chains is now introduced in the form of a *n* × 2 matrix Γ, where Γ_*ij*_ (Γ_*i*_(*d*)) is the proportion of disaccharide *j* (*d*) at position *i* from the non-reducing end. Matrix Γ has the following constraints:


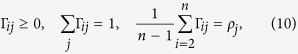


where *ρ*_*j*_ is the overall proportion of disaccharide number *j*. The last constraint preserves overall disaccharide composition. Assuming independence between positions, the N&I model yields the following relative abundance of species *s* = *d*_1_... *d*_*n*_:


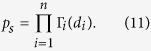


These abundances preserve overall disaccharide composition *ρ* when Γ satisfies constraints (10). Composition Γ_**1**_ at the non-reducing end does not intervene in calculations summarized next. Combining random drawing of one chain with equation (11) to its random cleavage by a *heparinase* and utilizing probability calculus yields distribution *g*(*l*) of fragment length *l* which is expected under N&I:


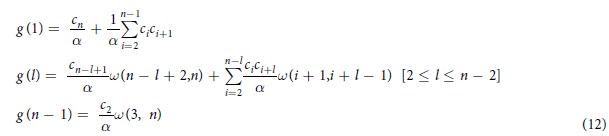


with 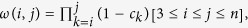 *α* = (*n* − 1)*c*, *c* = ∑_*b*_*c*(*b*)*ρ*(*b*) and *c*_*i*_ = ∑_*b*_*c*(*b*)Γ_*i*_(*b*) [2 ≤ *i* ≤ *n*].

Because quantities *ω* can be efficiently computed, the problem of optimizing Γ so as to fit experimentally observed distribution *f*_*z*_(*l*) of fragment length in *heparinase z* digest can be solved in reasonable time:


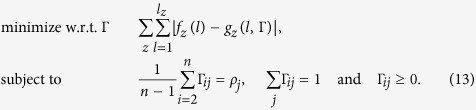


**Figure 3:**
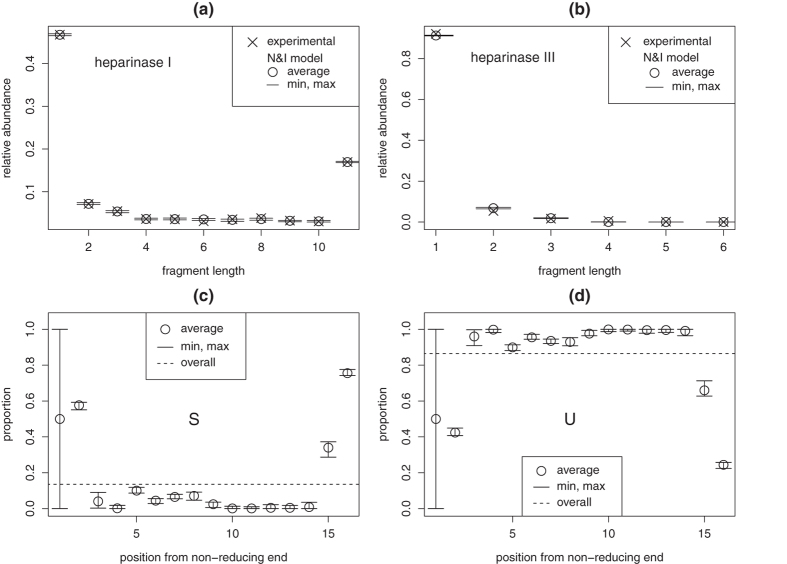
Model N&I. Distributions of fragment length *l* in *heparinases* I (**a**) and III (**b**) digests (crosses) as compared to distributions expected under model N&I (dots). Optimized profiles of S (**c**) and U (**d**) proportions along chains. Modeling results are summarized for 100 runs of optimization with different initial conditions.

Approximate solutions are obtained with simulated annealing while constraints are enforced at each perturbation by projection. Results are presented in Fig. 3(a,b). While fit is better than with combination H&C, the N&I model still cannot perfectly reproduce experimental data: the objective function in optimization problem (13) never goes below 0.04 (supplementary material). Similar results are obtained with different values of chain length *n* (supplementary material). Inability of model N&I to reproduce experimental data is best seen by examining optimized matrices Γ. Figure 3(c,d) displays optimized values of Γ_*ij*_ as a function of position *i*, i.e. the obtained profile of S/U composition along chains. Composition at position *i* = 1 is not constrained by experimental data and thus takes any possible value. For other positions, the obtained pattern is overrepresentation of S at chain extremities. This yields high abundance of long stretches of U, which is required to yield high abundance of long fragments in modeled *heparinase* I digest. This however implies having about 75% of S at the reducing end. It will be shown later that such a high proportion is in contradiction with experimental data which allow only for a maximum of 56%. Adding this upper bound in constraints of optimization problem (13) and running again simulated annealing yields final values of the objective function which are about three times larger (supplementary material). Therefore, nonhomogeneity alone is unable to reproduce experimental data.

Since models which correspond to combinations H&I, H&C and N&I cannot reproduce experimental data, the only remaining possibility is combination N&C of nonhomogeneity and correlation.

## Quantification of Nonhomogeneity and Correlation

To quantify nonhomogeneity and correlation of sulfation along BKHS chains, optimization framework (2) is utilized. Results are first presented with a mixture where all chains have same length *n* = 16 disaccharides and later extended to the case of a mixture of chain lengths between 10 and 20 disaccharides.

### Model with one chain length

The first step consists in expressing experimental measurements **b** as linear constraints **Ap** = **b** on vector **p** of molecular species abundances. For overall proportion *ρ*_*j*_ of disaccharide *j* between positions 2 and *n* one has


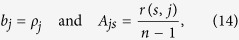


where *r*(*s*, *j*) is the number of disaccharide *j* in molecular species *s*. Calling *f*_*z*_(*l*) the relative abundance of fragments of length *l* after digestion by *heparinase z* and *d*_1_... *d*_*n*_ the sequence of species *s*, the resulting constraint is


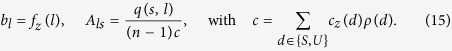


Numerator *q*(*s*, *l*) of *A*_*ls*_ is the expected number of fragments of length *l* when cleaving sequence *s* by *heparinase z* and the denominator is the expected number of fragments in the entire mixture. Expressions of *q*(*s*, *l*) are as follows:


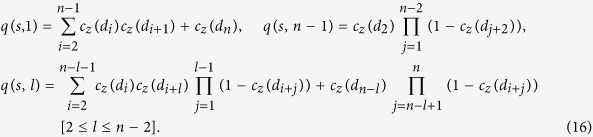


After including a constraint for all abundances summing to 1, overall disaccharide composition provides one linearly independent constraint and *heparinase* digests provide fifteen more constraints. Compatibility between constraints is then tested by solving a phase-I linear problem with artificial variables **x**:


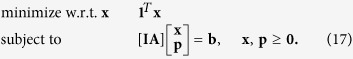


**Figure 4:**
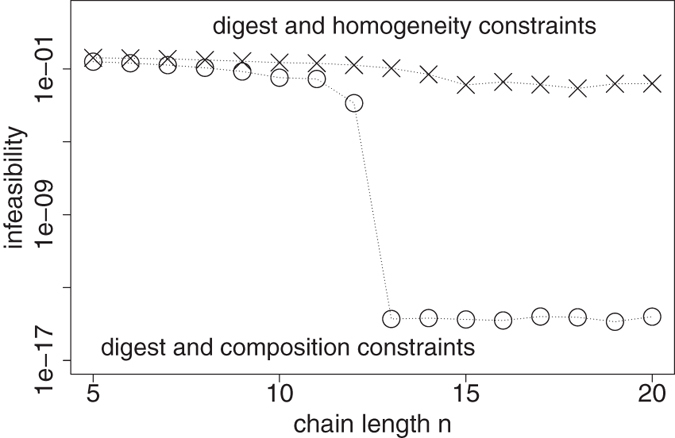
Infeasibility of constraint sets as a function of BKHS chain length *n*. Dots: constraints of *heparinase* digests and overall disaccharide composition. Crosses: constraints of *heparinase* digests and homogeneity.

The final value of the objective function is called infeasibility and should be close to numerical precision if constraints are compatible with each other. Obtained values of infeasibility are presented as a function of BKHS chain length *n* in Fig. 4. Dots correspond to the combination of *heparinase* digest and overall disaccharide composition constraints. Infeasibility becomes close to numerical precision when *n* ≥ 13. Since the experimentally determined average BKHS chain length is *n* = 16 disaccharides, one can state that experimental measurements are compatible with each other because there exist vectors **p** of species abundances which can explain all measurements. Solving a different feasibility problem provides another evidence that homogeneity of sulfation level along chains is not supported by *heparinase* digest data. Constraint (14) is replaced with constraints imposing same disaccharide composition at each position *i* from the non-reducing end:


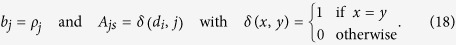


Estimated values of infeasibility with these constraints combined with those of *heparinase* digests are displayed with crosses as a function of BKHS chain length *n* in Fig. 4. While infeasibility dips around the estimated average chain length *n* = 16, it never reaches numerical precision and thus provides another evidence for nonhomogeneity of sulfation level along chains.

**Figure 5:**
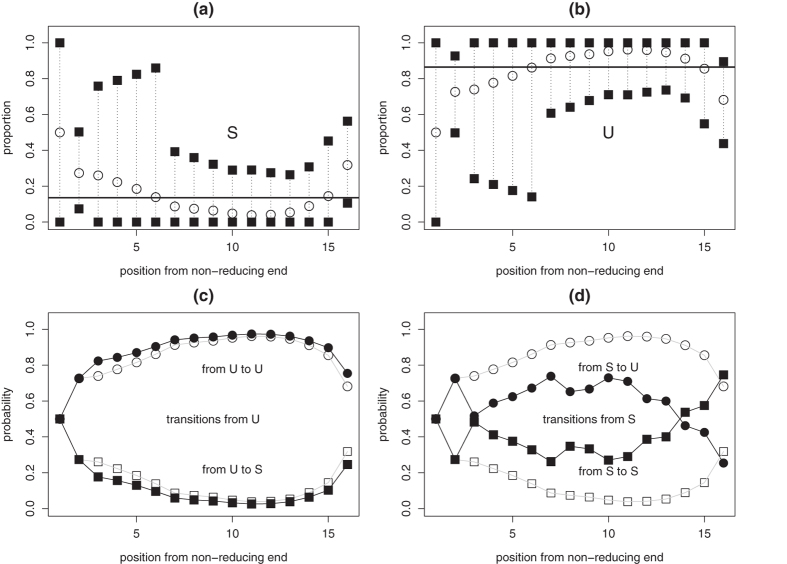
(**a,b**) Maximum-entropy estimates of S and U composition along BKHS chains (circles) and lower and upper bounds at each position estimated via linear programming (squares). Horizontal lines display overall S and U proportions. (**c,d**) Profiles of transition probabilities between disaccharides along chains, as estimated with maximum-entropy modeling (black symbols) and compared to the profile of disaccharide composition along chains (white symbols).

With only constraints (14) and (15) the problem of estimating **p** is highly underdetermined (17 constraints for more than 65,000 variables). A standard approach for underdetermined problems is maximum-entropy modeling^28^. This corresponds to optimization framework (2) with the objective function set to the negative entropy of **p**: 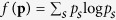. This problem is convex and it can be solved via its dual^22^,^29^, a geometric program in convex form for which efficient algorithms exist^29^,^30^. Once maximum-entropy model p^*^ has been estimated, resulting mixture properties are examined. Circles in Fig. 5(a,b) display the profile of S and U proportions along BKHS chains as obtained with model p^*^. Comparing maximum-entropy values to overall disaccharide composition (horizontal lines) suggests that sulfated disaccharides S are more likely to be abundant near the non-reducing end (2 ≤ *i* ≤ 6). High abundance of S is also possible at the reducing end (*i* = 16). It is informative to compare relative abundances of BKHS sequences obtained under p^*^ to abundances estimated with a less constrained maximum-entropy model based only on overall disaccharide composition, i.e. model H&I. In both models the most abundant species is xU_15_, where x stands for the disaccharide at the non-reducing end. However, abundances of species containing S near the non-reducing end or repeats of S tend to be higher with p^*^ than with H&I. For instance, species xS_4_U_11_ is about 139 times more abundant under p^*^ than under H&I. Further characterization of model p^*^ is provided in the supplementary material.

Maximum-entropy modeling provides one particular point p^*^ of the polyhedral set 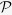 defined by experimental constraints. One can in addition explore boundaries of 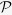 with linear programming. This is illustrated with the profile of S/U composition along chains. For a given position *i* from the non-reducing end, the objective function in optimization framework (2) is now set to the following linear function: *f* (**p**) = *α***c**^*T*^ **p** with *α* = ±1 and *c*_*s*_ = 1 if species *s* has sulfated disaccharide S at position *i* and 0 otherwise. Solution to this problem for *α* = 1 yields the lower bound for proportion of S at position *i* and setting *α* = −1 gives the upper bound. Estimated bounds are displayed with black squares in Fig. 5(a,b). Because utilized experimental measurements do not provide information about non-reducing end, composition at position *i* = 1 is not restricted. For *i* ≥ 2, ranges of allowed proportions of sulfated disaccharide S vary with *i*. Consistent with maximum-entropy results, higher proportions of S are allowed towards the non-reducing end (2 ≤ *i* ≤ 6), while U must represent at least 50% of the disaccharides near the reducing end (7 ≤ *i* ≤ 14). Another noticeable result displayed in Fig. 5(a) is the upper bound of 56% for proportion of S at the reducing end (*i* = 16). This is lower than the 75% required by model N&I to best fit *heparinase* digest data (Fig. 3(c,d)) and thus confirms that model N&I cannot perfectly reproduce experimental data.

Patterns of nonhomogeneity are rather opposite between model N&I (Fig. 3(c,d)) and maximum-entropy model p^*^ (Fig. 5(a,b)); the latter perfectly reproduces experimental data while having more S towards the non-reducing end. This is possible thanks to strong correlation. Black symbols in Fig. 5(c,d) show transition probabilities between disaccharides at each position *i* which are estimated with p^*^. White symbols show disaccharide composition at each position *i* under p^*^ and represent transition probabilities expected in the absence of correlation. Transition probabilities from U at *i* to S/U at *i* + 1 do not deviate much from an independence model. Transition probabilities from S show stronger deviations from independence and correlation is more pronounced near the reducing end. In summary, utilizing optimization framework (2) suggests that disaccharides S are likely to be less abundant near the reducing end but, when present there, might display block structures.

### Model with a mixture of chain lengths

**Figure 6:**
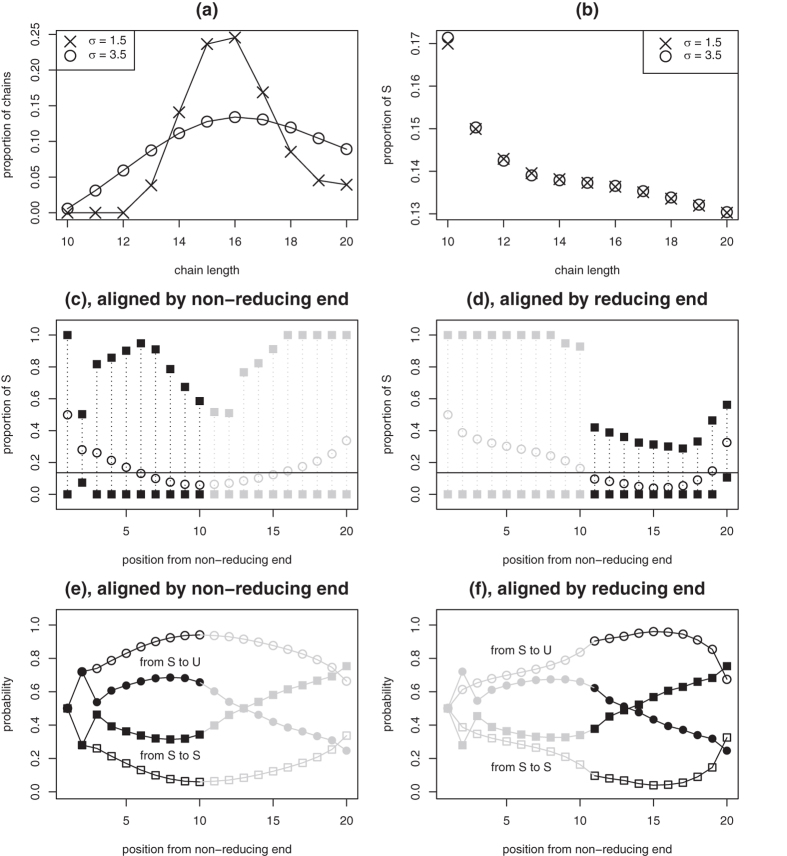
(**a,b**) Two models of BKHS chain length distributions (**a**) and resulting chain disaccharide composition as a function of their length under maximum-entropy modeling (**b**). (**c–f**) Estimations of nonhomogeneity and correlation profiles when BKHS is modeled as a mixture of chain lengths (10 ≤ *n* ≤ 20, *σ* = 3.5). Chains are either aligned by their non-reducing end (**c,e**) or their reducing end (**d,f**). (**c,d**) maximum-entropy composition profile (circles), bounds at each position (squares) and overall composition (horizontal line). (**e,f**) transition probabilities from S to S or U (black) compared to probabilities under independence (white).

Results presented so far were with a model in which all BKHS chains have same length *n*. To check that this does not induce nonhomogeneity and correlation, results are verified with models where chain length has nonzero variance. Call **n** = (*n*^−^, ..., *n*^+^) a vector of increasing chain lengths and **w** the vector of their relative abundances. Distribution **w** has the following constraints: **w** ≥ **0**, **1**^*T*^**w** = 1 and **n**^*T*^**w** = 16, the last one imposing an average chain length of 16 disaccharides. Simple models of **w** are obtained by first setting *w*(*n*) to the integral of a Gaussian density of mean 16 and standard deviation *σ* between lengths *n* and *n* + 1, and then projecting onto the polyhedral set defined by distribution constraints. Panel (a) of Fig. 6 provides two examples of modeled chain length distributions **w** for *σ* = 1.5 and *σ* = 3.5, when *n*^−^ = 10 and *n*^+^ = 20.

The set of all molecular species is now the set of all S/U sequences of length between 10 and 20. Distribution **w** translates into linear constraints **Ap** = **b** on vector **p** of species abundances: *b*_*n*_ = *w*(*n*) and *A*_*nj*_ = 1 if species *j* has length *n* and 0 otherwise. After adding constraints for overall disaccharide composition and *heparinase* digest data, optimization framework (2) is utilized to examine properties of the mixture. Panel (b) of Fig. 6 shows one remarkable property obtained with maximum-entropy model p^*^: short chains have higher overall proportion of S. A relevant question is then whether variation of S with chain length is implied by *heparinase* digest constraints or rather just possible and occurring under maximum-entropy modeling. Adding constraints of same overall disaccharide composition for all chain lengths and solving the resulting feasibility problem (17) yields that there exist feasible vectors **p**. Relationship between chain length and disaccharide composition is therefore information which was not provided by *heparinase* digests. In other words, utilizing optimization framework (2) suggested that characterization of BKHS size fractions is an informative complementary approach.

Results obtained with optimization framework (2) for *σ* = 3.5, *n*^−^ = 10 and *n*^+^ = 20 are summarized in Fig. 6(c–f). Because chains have now diverse lengths, properties near the non-reducing end are best estimated after aligning chains by their non-reducing end. Likewise, alignment by reducing end is utilized to examine properties near the reducing end. Panels (c,d) of Fig. 6 show maximum-entropy S composition as a function of position in chains (circles), bounds on S at each position (squares) and overall composition (horizontal line). The pattern is similar to that obtained when all chains have length *n* = 16: larger proportions of S are allowed towards the non-reducing end. Note that estimated bounds near position 20 are 0 and 1 when chains are aligned by their non-reducing end. This can be achieved while preserving all constraints because only a subset of chains have length 20. Bounds near the reducing end are better estimated after aligning by reducing end, because this estimation includes all chains. Result is then that proportion of S is more limited near the reducing end. Panels (e,f) of Fig. 6 show profiles of transition probabilities along chains from S to U or S as estimated by maximum-entropy modeling (black) and probabilities which would correspond to independence (white). Results are similar to those obtained when all chains have same length: maximum-entropy modeling suggests overrepresentation of species having stretches of sulfated disaccharides S and, while overall proportion of S is likely less near the reducing end, block structures are more likely at this end.

In summary, utilizing optimization framework (2) provides insight into the potential structure of BKHS with only seventeen linearly independent constraints even though the last considered BKHS mixture model represents more than two million individual species. Optimization framework (2) suggests nonhomogeneity in the form of higher sulfation levels towards non-reducing end and higher correlation of sulfation state between adjacent disaccharides near reducing end. Nonhomogeneity and correlation might reflect different aspects of sulfation mechanisms which take place during BKHS biosynthesis.

## Discussion

Applying constrained-optimization framework (2) to BKHS experimental measurements showed that combining a few selected measurements can provide deep insight into the structure of a mixture having potentially millions of molecular species. Maximum-entropy modeling was the obvious first approach to model a mixture with only a few measurements. Yet, linear programming provided a more critical view of mixture properties. First, it demonstrated inability of homogeneity to explain experimental data. Second, it provided bounds on nonhomogeneity along chains, thereby estimating the extent of characterization achieved by the set of measurements with respect to this feature. Intervals provided by bounds account for remaining uncertainty and thus would be appropriate, for instance, to compare the sulfation pattern along HS chains in two different tissues. Non-overlapping intervals would hint at dissimilarity while overlapping intervals would not preclude similarity. Third and finally, linear programming showed that characterizing BKHS size fractions yields information complementary to *heparinase* digests. One potential limitation of optimization framework (2) is a very large number of molecular species. Because constraint matrices are sparse, memory usage and computation time can be reduced by implementing special linear algebra and optimization methods^31^,^32^. In the case of BKHS, taking into account not only 2- *O*-sulfation but also N-sulfation, 3- *O*-sulfation and 6- *O*-sulfation results in sixteen possible states for each disaccharide and the number (16)^*n*^ of species becomes prohibitive for utilizing optimization framework (2). One would then have to explore the possibility of working with a parametric model, i.e. optimization framework (3) with a nonhomogeneous Markov model having 256 × *n* parameters. More generally, when the number of species is too large, a modeling choice such as partial independence might be required. Consider for instance that individual species are defined by sequences of three attributes: *abc*. If overall composition of *c* is known, assuming independence of *c* with other attributes yields *p*(*a*_*i*_*b*_*j*_*c*_*k*_) = *p*(*a*_*i*_*b*_*j*_)*p*(*c*_*k*_). The mixture is then represented by all *ab* sequences, a vector of smaller dimension. Contribution *h*(*a*_*i*_*b*_*j*_) of sequence *a*_*i*_*b*_*j*_ to a constraint is given by ∑_*k*_*h*(*a*_*i*_*b*_*j*_*c*_*k*_)*p*(*c*_*k*_), which can be estimated via sequence enumeration.

Optimization framework (3) was utilized to demonstrate that only combination of nonhomogeneity and correlation could reproduce experimental data. This required deriving distribution of fragment length in a *heparinase* digest as an explicit function of parameters of a species abundance model. Calculation methods were briefly mentioned and are detailed in the supplementary material. These methods can be applied to other properties of fragments. One could for instance derive the expected disaccharide composition of fragments of length *l*. These equations would explain how nonhomogeneity along BKHS chains translate into potential variation of disaccharide composition with fragment length *l*. Such a mathematical exercise explains how properties of cleavage fragments, which are in general easier to experimentally characterize, reflect features of uncleaved chains. Moreover, as was illustrated with model H&I, some equations can be analyzed independently of parameter values and can then yield strong statements.

In conclusion, constrained optimization and mathematical modeling can yield deep insight into the structure of complex biochemical mixtures when applied to the combination of a small number of measurements. Careful selection of measurement types is crucial to provide complementary views of a complex mixture^4^,^10^, so that these measurements can be efficiently combined to estimate extent of characterization via linear programming. Application of the methods presented in this paper to different types of mixtures, development of alternate computational methods and further exploration of mathematical approaches are likely to help advance our understanding of complex mixtures.

## Additional Information

**How to cite this article**: Pradines, J. R. *et al.* Combining measurements to estimate properties and characterization extent of complex biochemical mixtures; applications to Heparan Sulfate. *Sci. Rep.* **6**, 24829; doi: 10.1038/srep24829 (2016).

## Supplementary Material

Supplementary Information

## Figures and Tables

**Table 1:** Experimental measurements on bovine kidney heparan sulfate.

(a)			
category	proportion	*heparinase* I yield	*heparinase*III yield
S	0.1358	1.000	0.033
U	0.8642	0.030	1.000
**(b)**			
***heparinase*** **I digest**		***heparinase*** **III digest**	
**fragment length**	**proportion**	**fragment length**	**proportion**
1	0.4676	1	0.9211
2	0.0711	2	0.0545
3	0.0544	3	0.0164
4	0.0361	4	0.0058
5	0.0351	5	0.0018
6	0.0307	≥6	0.0004
7	0.0353		
8	0.0380		
9	0.0321		
10	0.0302		
≥11	0.1694		

(a) overall proportions of disaccharides categories S and U, excluding the non-reducing end, and *heparinase* cleavage yields. (b) distributions of fragment length (number of disaccharides) after *heparinase* I or III digestion.
