# Supplementary material for: Combining measurements to estimate properties and characterization extent of complex biochemical mixtures; applications to Heparan Sulfate
Source: Sci Rep. 2016 Apr 26;6:24829. doi: 10.1038/srep24829 (PMC4845011; doi:10.1038/srep24829)
Supplement: Supplementary Information [file srep24829-s1.pdf]

Combining measurements to estimate properties and characterization extent of complex biochemical mixtures; applications to Heparan Sulfate

Supplementary material

Joël R Pradines, Daniela Beccati, Miroslaw Lech, Jennifer Ozug, Victor Farutin, Yongqing Huang, Nur Sibel Gunay and Ishan Capila.

Momenta Pharmaceuticals Inc., Research Department, Cambridge, MA 02142, USA.

Contents

1 Experimental material, methods and data **2**

2 Parametric models of molecular species abundances **6**
2.1 Notations and calculation methods 6
2.2 Homogeneity and independence 7
2.2.1 Model of species abundances 7
2.2.2 Model of digest 8
2.3 Homogeneity and correlation 10
2.3.1 Model of species abundances 10
2.3.2 Model of digest 11
2.3.3 Estimation of model parameters 13
2.4 Nonhomogeneity and independence 16
2.4.1 Model of species abundances 16
2.4.2 Model of digest 17
2.4.3 Estimation of model parameters 19

3 Non-parametric modeling **21**
3.1 Constraints on species abundances 21
3.2 Linear programming 23
3.3 Maximum-entropy modeling 23

4 Source code **26**
4.1 Input files 26
4.2 Summary of classes 28
4.3 Code 29

1 Experimental material, methods and data

Material

The utilized Heparan Sulfate (HS) material is commercially available Bovine Kidney HS (BKHS) (1mg, Seikagaku).

Quantification by UV absorbance at 232 nm

Quantification of relative abundances of di- and tetrasaccharides (overall composition analysis) and BKHS fragments after *heparinase* I or III digestion was reliably performed by measuring UV absorbance at 232 nm. Absorbance at 232 nm occurs only after cleavage of a BKHS chain by a *heparinase* between the glucosamine and downstream uronic acid residues. This implies that relative abundances of saccharides at the non-reducing end of BKHS chains were not measured and this fact was taken into account for mathematical modeling.

Overall oligosaccharide composition analysis

BKHS was digested at 30 ^oC for 16h using a cocktail of *Bacteroides Heparinases* I, III, and IV manufactured in house. The digested sample was then incubated for 6 hours at 30 ^oC simultaneously with *2-O-sulfatase* and $\Delta^{4,5}$*-glycuronidase*. The digested sample was frozen and lyophilized.

Supplementary Table S1: Overall proportions of individual disaccharides and tetrasaccharides (excluding the non-reducing end) and their grouping into categories U and S which reflect their different propensities for cleavage by *heparinase* I and *heparinase* III. Length is the number of disaccharides.

description	proportion	length	category	category proportion
ΔUH_{NS}	0.1644	1	U	
ΔUH_{NAc6S}	0.1042	1	U	
ΔUH_{NS3S}	0.0005	1	U	
ΔUH_{NS6S}	0.0597	1	U	
$\Delta U_{gal}H_{NS6S}$	0.0010	1	U	
ΔUH_{NAc}	0.5232	1	U	
$\Delta U_{gal}H_{NS}$	0.0053	1	U	
$\Delta UH_{NAc6S}GH_{NS3S}$	0.0050	2	U	
$\Delta UH_{NAc6S}GH_{NS3S6S}$	0.0008	2	U	
			U	0.8642
$\Delta U_{2S}H_{NAc}$	0.0077	1	S	
$\Delta U_{2S}H_{NS}$	0.0785	1	S	
$\Delta U_{2S}H_{NAc6S}$	0.0014	1	S	
$\Delta U_{2S}H_{NS6S}$	0.0482	1	S	
			S	0.1358

Digested BKHS was then analyzed by Ion-Pairing Reverse Phase HPLC (IPRP-HPLC) using 30 mM tetra-n-butyl ammonium chloride (TBA) as the ion-pairing reagent in 15% acetonitrile (ACN). The digested sample was separated using an analytical C18 Discovery column (4.6 x 250 mm, Supelco) maintained at 25 ^oC, with a flow rate of 0.7 mL/min over 130 min of total run time, with a gradient ranging from 0 to 1 M NaCl. The elution profile was monitored by UV absorption at 232 nm. Peaks were identified by co-injection with commercial standards or through peak isolation and characterization by mass spectroscopy and NMR spectroscopy. The UV elution profile at 232 nm was integrated to determine the relative

abundances of di- and tetrasaccharides listed in table S1.

Note that to simplify mathematical treatment, all units in modeled chains were considered to be disaccharides (length 1), even though category U contains two tetrasaccharides (length 2). This simplification was deemed reasonable, because tetrasaccharides represent less than 0.6% of all units.

Estimation of average BKHS chain length

2D-NMR was utilized to estimate the average chain length of BKHS. The two-dimensional HSQC spectra of BKHS was recorded at 298 K, with a 600 MHz Bruker Avance spectrometer equipped with a 5 mm triple-resonance inverse cryoprobe. Before spectra acquisition, BKHS was dissolved at 2 mg/150 μL of D2O (99.9%) and sonicated for 30 s to remove air bubbles. HSQC spectrum was recorded with sensitivity enhancement and carbon decoupling during acquisition, for 64 scans of 256 increments. The polarization transfer delay was set with a ^{1}JC-H coupling value of 155 Hz. For HSQC spectra, the matrix was zero filled to 2K x 1K prior to Fourier transformation. The relative amount of linkage region and N-acetyl glucosamine, residues which are present at the reducing end of BKHS chains, versus the amount of disaccharides internal to the chain, was used to estimate an average chain length of 16 disaccharides.

Estimation of fragment length distributions in heparinases I and III digests

BKHS (0.6 mg) was digested at 30 ^{o}C for 16 h with *Bacteroides Heparinase* I (5 IU/mg) or III (2 IU/mg). On completion of digestion, the sample was frozen, lyophilized, and separated on a chromatographic system consisting of the Ultimate 3000 with Dual-Ternary Capillary HPLC pumps (Dionex) and two 4.6mm x 300mm, 4μm gel permeation TOSOH TSKgel SuperSW2000 columns placed in series and equilibrated at 25 ^{o}C. 100mM ammonium acetate was used as eluent at the constant flow-rate of 100μl/min delivered by ternary solvent system. The wavelength of the UV detector (Dionex) was set at 232 nm, followed by the Dionex 2mm Anion Self-Regenerating Suppressor 300 (ASRS-300 (2-mm)) using water as regenerant. Electrolysis of water in the ASRS-300 was driven by the Dionex SRS controller using the 100mA current. The flow was post-column controlled by QuickSplit adjustable flow splitters (Analytical Scientific Instrument) to a constant splitting ratio of 1:10. Fragments in eluted peaks were identified by mass spectrometry and relative abundances were estimated by integration of the UV profile at 232 nm (Chromeleon, version 6.80, Dionex^{TM}). UV profiles and integration results are provided in figure S1.

Cleavage yields of heparinases I and III

To determine the specificity of *heparinase* I , BKHS was digested with 5 IU/mg *heparinase* I and the digest was analyzed by ^{1}H-NMR and GPC. *Heparinase* I is known to primarily cleave the $H_{NS6X} - I_{2S}$ linkage present in highly sulfated regions of HS, converting I_{2S} into Δ_{U2S}. The ^{1}H-NMR spectrum of BKHS was recorded at 298 K, with a 600 MHz Bruker Avance spectrometer equipped with a 5 mm triple-resonance inverse cryoprobe. Before spectra acquisition, BKHS was dissolved at 2 mg/150 μL of D2O (99.9%) and sonicated for 30 s to remove air bubbles. ^{1}H-NMR spectrum was acquired with presaturation of the residual water signal, with a recycle delay of 5 s for 64 scans.

The spectrum of BKHS digested with *heparinase* I showed peaks due to Δ_{U2S} and Δ_U, indicating that this enzyme was also able to cut between glucosamine and non-sulfated uronic acid residues (see figure S2). Under the utilized experimental conditions, *heparinase* I converted all the sulfated uronic acids present in BKHS into Δ_{U2S} residues (cleavage yield of

Supplementary Figure S1: GPC UV profiles (at 232 nm) and integration results for BKHS digested with *heparinase* I (left) or *heparinase* III (right). Note that 'Dp2' corresponds to a length of 2 monosaccharides, i.e. to a length of 1 disaccharide.

heparinase I

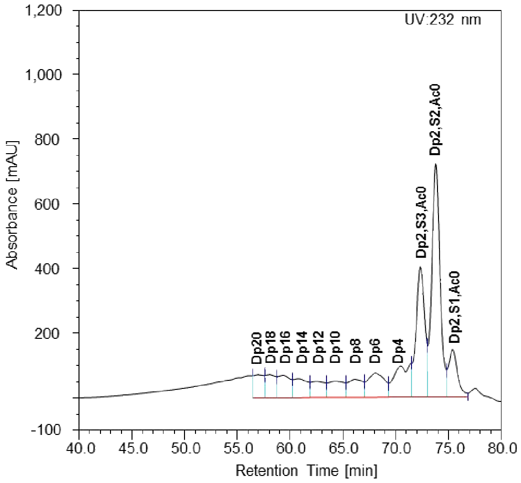

heparinase III

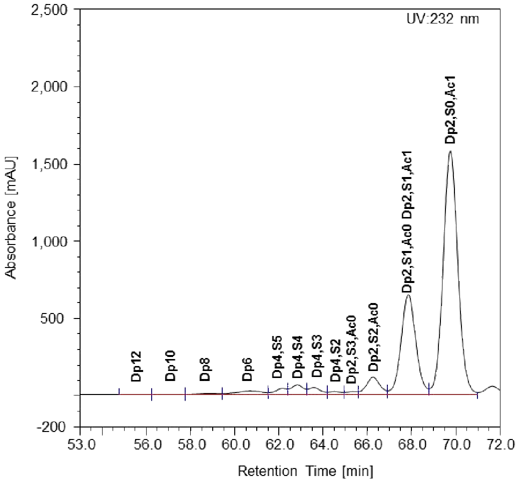

length	proportion
1 (Dp2)	0.4676
2 (Dp4)	0.0711
3 (Dp6)	0.0544
4 (Dp8)	0.0361
5 (Dp10)	0.0351
6 (Dp12)	0.0307
7 (Dp14)	0.0353
8 (Dp16)	0.0380
9 (Dp18)	0.0321
10 (Dp20)	0.0302
$\geq$ 11 (Dp22+)	0.1694

length	proportion
1 (Dp2)	0.9211
2 (Dp4)	0.0545
3 (Dp6)	0.0164
4 (Dp8)	0.0058
5 (Dp10)	0.0018
$\geq$ 6 (Dp12+)	0.0004

100% for disaccharide category S). In addition, it generated an amount of Δ_{U} corresponding to 2.6% of the total uronic acid content, i.e. 3% of the non-sulfated uronic acid content (cleavage yield of 3% for disaccharide category U). Similar results were obtained with analysis of GPC data. The presence of disaccharides of formula Dp2,S1,Ac0 = $\Delta\mathrm{UH}_{\mathrm{NS}}$; Dp2,S1,1Ac = $\Delta\mathrm{U}_{2\mathrm{S}}\mathrm{H}_{\mathrm{NAc}}$ or $\Delta\mathrm{UH}_{\mathrm{NAc6S}}$ provided evidence that *heparinase* I could also cleave non-sulfated residues. The relative amount of these disaccharides was calculated as 2.5% of the total disaccharide content, which corresponds to 2.9% of the non-sulfated disaccharides (cleavage yield of about 3% for disaccharide category U).

To evaluate cleavage yields by *heparinase* III, BKHS was digested with 2 IU/mg of this enzyme. All non-sulfated disaccharides were cleaved (cleavage yield of 100% for disaccharide category U). GPC data indicated that *heparinase* III could also cleave part of the fully sulfated region, generating an amount of disaccharide Dp2,S3,Ac0 = $\Delta\mathrm{U}_{2\mathrm{S}}\mathrm{H}_{\mathrm{NS6S}}$ corresponding to slightly less than 0.5% of the total disaccharides content. Since sulfated disaccharides

Supplementary Figure S2: ^{1}H-NMR spectrum of BKHS digested with *heparinase* I.

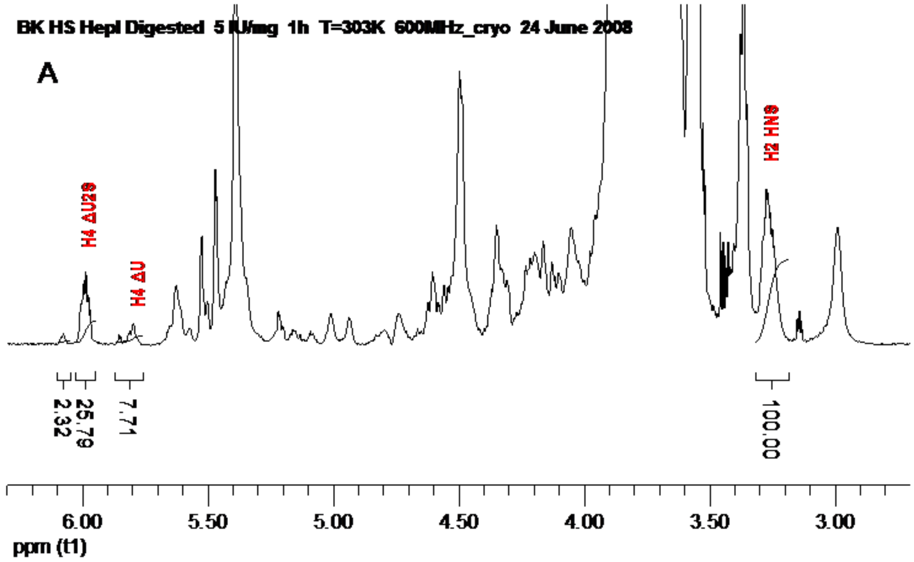

constitute 14% of the total disaccharide content, cleavage yield for disaccharide category S by *heparinase* III was estimated at approximately 3.3%.

2 Parametric models of molecular species abundances

2.1 Notations and calculation methods

Supplementary Table S2: Main symbols utilized for modeling of species abundances.

symbol	meaning
$\mathbf{A}$	matrix (uppercase bold)
$\mathbf{b}$	vector (lowercase bold)
n	length of a BKHS chain
i	position from the non-reducing end ($1 \leq i \leq n$)
d	disaccharide
$\mathcal{D}$	set of all disaccharides
$m = \lvert\mathcal{D}\rvert$	number of disaccharides
d_i	disaccharide at position i
D_i	random variable for disaccharide at position i
$\mathcal{C}_i$	event of cleavage at position i
$\Pr(\mathcal{E})$	probability of event $\mathcal{E}$ under a given random experiment
$\rho(d)$	overall proportion of disaccharide d over positions 2 to n
$\boldsymbol{\rho}$	vector of all disaccharide overall proportions
$s = d_1 \dots d_n$	sequence of a BKHS chain
p_s	relative abundance of sequence/species s among all m^n BKHS sequences
$\mathbf{p}$	vector of relative abundances of all possible BKHS sequences
$\boldsymbol{\gamma}$	disaccharide composition at the non-reducing end
$c_z(d)$	cleavage yield of *heparinase* z for disaccharide d
c	average cleavage yield based on overall composition $\boldsymbol{\rho}$
l	digestion fragment length
L	random variable for fragment length
g	modeled distribution of fragment length
f_z	experimentally measured distribution of fragment length for *heparinase* z
P_{ab}	transition probability from disaccharide a at position i to b at $i+1$
$\mathbf{P}$	$m \times m$ matrix of transition probabilities
Γ_{ij} or $\Gamma_i(d_j)$	proportion of disaccharide d_j at position i
$\boldsymbol{\Gamma}$	$n \times m$ matrix of disaccharide composition

A molecular species is defined as all BKHS chains having a given length n (in disaccharides) and a given S/U disaccharide sequence from non-reducing end to reducing end. The sequence of a species s is symbolized as follows:

$$\text{(non-reducing end)} \quad d_1 d_2 \dots d_i \dots d_n \quad \text{(reducing end)},$$

where d_i stands for disaccharide at position i, with the arbitrary convention of ordering positions from non-reducing end ($i = 1$) to reducing end ($i = n$). Relative abundance of species s is noted p_s and $\mathbf{p}$ stands for the vector of all m^n species abundances, if m is the number of disaccharides.

There exists an obvious relationship between the concept of relative abundance and that of probability. Indeed, consider the following random experiment: "randomly choose one

BKHS molecule inside the mixture". The probability of choosing species s is relative abundance p_s. Likewise, a model of species abundances can be defined as a random experiment under which a chain is built. Such a random experiment does not intend to mimic the actual biosynthesis process, but only to model its output: species abundances. Definition of this random experiment depends on properties which are attributed to a model of species abundances: homogeneity vs. nonhomogeneity and independence vs. correlation.

Formalizing generation of a chain as the output of a random experiment is mathematically useful. Indeed, one can then calculate properties of the mixture as probabilities or expected values under this random experiment. Cleavage of a BKHS chain by a *heparinase* can also be defined as a random experiment: cleavage at position i means cleavage between disaccharides d_{i-1} and d_i

$$d_1 d_2 \ldots d_{i-1} \mid d_i \ldots d_n \quad \text{(cleavage at } i\text{)}.$$

and it happens with probability $c\left(d_i\right)$, which is the *heparinase* cleavage yield for this disaccharide. Then, a random variable L for length of a digestion fragment is defined under the combined random experiments of building a chain and cleaving it. Under this new random experiment, the probability distribution of L yields the distribution of fragment length which is implied by properties of the model and by its parameters.

The above approach is utilized with three models of species abundances: Homogeneity and Independence model (H&I), Homogeneity and Correlation model (H&C) and Nonhomogeneity and Independence model (N&I). For each model, distribution of fragment length after *heparinase* digestion is derived as an explicit function of enzyme cleavage yields, overall disaccharide composition and model parameters. These equations are then utilized to optimize model parameters so as to best fit experimental data of *heparinase* digests while preserving overall disaccharide composition. Table S2 provides a glossary of most symbols utilized for mathematical modeling.

2.2 Homogeneity and independence

2.2.1 Model of species abundances

In this model of species abundances there is one main parameter: the vector $\boldsymbol{\rho}$ of overall relative abundances of disaccharides over positions $i = 2$ to n from the non-reducing end. In order to have a complete formalism, relative abundances of disaccharides at the non-reducing end ($i = 1$) are represented by a vector $\boldsymbol{\gamma}$, even though $\boldsymbol{\gamma}$ is not experimentally measured. Generation of one chain under H&I is defined by the following random experiment.

(**Random experiment R1).**
Randomly choose a disaccharide at the non-reducing end based on vector $\boldsymbol{\gamma}$, and, for each position $i = 2$ to n, randomly choose a disaccharide based on vector $\boldsymbol{\rho}$.

By definition of H&I, the identity of disaccharide at position $i + 1$ does not depend on the identity of disaccharide at position i and the relative abundance of species $s = d_1 \ldots d_n$ is

$$p_s = \gamma\left(d_1\right) \prod_{i=2}^{n} \rho\left(d_i\right). \tag{2.1}$$

One can show that this vector of m^n abundances has components which sum to 1

$$\mathbf{1}^T \mathbf{p} = 1 \tag{2.2}$$

because $\mathbf{1}^T \boldsymbol{\rho} = 1$ and $\mathbf{1}^T \boldsymbol{\gamma} = 1$. This vector also preserves disaccharide proportions

$$\mathbf{A}\mathbf{p} = \boldsymbol{\gamma} \quad \text{and} \quad \mathbf{B}\mathbf{p} = (n-1)\,\boldsymbol{\rho} \tag{2.3}$$

where entry A_{rk} of the $m \times m^n$ matrix $\mathbf{A}$ takes value 1 if species k has disaccharide r at the non-reducing and 0 otherwise, and entry B_{rk} of the $m \times m^n$ matrix $\mathbf{B}$ takes for value the total number of occurrences of disaccharide r in species k at positions other than the non-reducing end. One can show that (2.1) is solution to the optimization problem

$$\begin{array}{ll} \text{maximize w.r.t. } \mathbf{p} & -\sum_s p_s \log p_s \\ \text{subject to} & (2.2) \text{ and } (2.3) \end{array}.$$

This problem is known as maximum-entropy modeling subject to linear equality constraints. In general it has no explicit solution and must be solved numerically via its dual [1,2]. Here, because matrices $\mathbf{A}$ and $\mathbf{B}$ have special structures, it can be shown that (2.1) is solution.

2.2.2 Model of digest

Heparinase digestion of a BKHS chain is modeled with the following random experiment.

(Random experiment R1C).
Generate one BKHS chain via random experiment **R1** *and cleave at each disaccharide d_i for $2 \leq i \leq n$ with probability given by heparinase yield for d_i.*

The event of cleavage at position i is noted $\mathcal{C}_i$. Let $c(d) = \Pr(\mathcal{C}_i \mid D_i = d)$ stand for the probability of cleavage at position i under **R1C** and conditional to residue D_i being disaccharide d. Let c stand for this probability not conditioned to d. Because under **R1** all positions i have independent distributions of disaccharides, the probability of event $\mathcal{C}_i$ is the same at any position i:

$$\begin{aligned} c &= \sum_{d \in \mathcal{D}} \Pr(\mathcal{C}_i \wedge D_i = d) \\ &= \sum_{d \in \mathcal{D}} c(d)\,\rho(d). \end{aligned} \tag{2.4}$$

The distribution of fragment length after digestion is then calculated as a function of c and n. Let L be the random variable "length of one fragment obtained with one run of **R1C**". Let L_i be L conditioned to the fact that the fragment originates from position i. Because fragments originating from the non-reducing end ($i = 1$) are not experimentally detected, L_1 is excluded from calculation. The distribution of L_i is geometric and restricted to $l \leq n - i + 1$:

$$\Pr(L_i = l) = \begin{cases} c\,(1-c)^{l-1} & [\,2 \leq i \leq n - l\,] \\ (1-c)^{l-1} & [\,i = n - l + 1\,] \end{cases} \tag{2.5}$$

Let u_i stand for the proportion of fragments originating from position i. Because all positions $i \geq 2$ have probability c of generating a fragment, u_i is given by

$$u_i = \frac{c}{(n-1)\,c} = \frac{1}{n-1} \quad [\,2 \leq i \leq n\,]\,. \tag{2.6}$$

Combining this with (2.5) yields the distribution g of L for values $l \leq n-2$:

$$\begin{aligned} g\,(l) &= \sum_{i=2}^{n-l+1} \Pr\left(\mathcal{C}_i \wedge (L = l)\right) \\ &= \sum_{i=2}^{n-l+1} u_i \Pr\left(L_i = l\right) \\ &= \frac{(1-c)^{l-1}}{n-1} + \sum_{i=2}^{n-l} \frac{c\,(1-c)^{l-1}}{n-1} \\ &= \frac{(1-c)^{l-1}}{n-1} + \frac{(n-l-1)\,c\,(1-c)^{l-1}}{n-1}. \end{aligned}$$

The case $L = n-1$ is given by (2.5) and distribution of L is summarized with

$$g\,(l) = \frac{1 + (n-l-1)\,c}{n-1}\,(1-c)^{l-1} \quad [\,1 \leq l \leq n-1\,]\,. \tag{2.7}$$

Parameters are experimentally available : $n = 16$ is the average BKHS chain length and c can be computed via Equation (2.4) based on cleavage specificities $c\,(d)$ and overall disaccharide proportions $\rho\,(d)$. In practice, experimental data is limited to small values of l, but the proportion of fragments having length equal to or greater than an upper bound l_m is measured. This proportion of longer fragments is modeled as $\sum_{l=l_m}^{n-1} g\,(l)$.

Finally, it is stressed that the logarithmic transform of $g\,(l)$ is close to a linear function of l:

$$\ln g\,(l) = l \ln(1-c) + \ln\left(1 + (n-l-1)\,c\right) - \ln(n-1) - \ln(1-c)\,.$$

The first term is linear. The second term is non-linear. However, if c is close to zero, the second term behaves like $c\,(n-l-1)$, i.e. another linear function of l. Non-linearity of the second term can be pronounced for c close to 1 and large values of l. But in this case, the coefficient of the first term becomes large an this term dominates the sum.

Consider now the case of a distribution $\mathbf{w}$ of BKHS chain length n with non-zero variance:

$$1 < n_i \leq n \leq n_s, \quad w\,(n) \geq 0, \quad \sum_{n=n_i}^{n_s} w\,(n) = 1.$$

Then, proportion $h\,(l)$ of fragments of length l is a weighted sum of functions $g\,(l,n)$:

$$\begin{aligned} h\,(l) &= \sum_{n=\max n_i, l+1}^{n_s} w\,(n)\,\frac{1 + (n-l-1)\,c}{n-1}\,(1-c)^{l-1}, \\ &= (1-c)^{l-1} \sum_{n=\max n_i, l+1}^{n_s} w\,(n)\,\frac{1 + (n-l-1)\,c}{n-1}. \end{aligned}$$

Therefore one has

$$\ln h(l) = l \ln(1-c) - \ln(1-c) + \ln \sum_{n=\max n_i, l+1}^{n_s} w(n) \frac{1+(n-l-1)c}{n-1},$$

so that the qualitative analysis given in the previous section for $g(l)$ also applies to $h(l)$: model H&I implies a nearly linear function for logarithmic abundance of fragment length.

2.3 Homogeneity and correlation

2.3.1 Model of species abundances

Model H&C allows for overrepresentation of species having long repeats of a given disaccharide category. In H&C, the identity of disaccharide at position $i+1$ depends on the identity of disaccharide at position i but not on the value of i. That is, sequences and their relative abundances are modeled by a homogeneous Markov model [3].

Let m be the number of disaccharides and $\mathbf{P}$ a matrix of transition probabilities; i.e. P_{ab} is the probability of observing disaccharide b on the right of disaccharide a. Call again $\rho(a)$ the overall proportion of disaccharide a over positions $i = 2$ to n. Matrix $\mathbf{P}$ has the following constraints

$$\begin{aligned} & P_{ab} \geq 0 && \text{(nonnegativity)}, \\ & \sum_b P_{ab} = 1 && \text{(stochastic matrix)}, \\ & \rho(b) = \sum_a \rho(a) P_{ab} && \text{(balance equations)}. \end{aligned} \tag{2.8}$$

The last condition combined to composition $\boldsymbol{\rho}$ at $i = 2$ guarantees that sequence abundances preserve disaccharide composition $\boldsymbol{\rho}$.

Given matrix $\mathbf{P}$ of transition probabilities, vector $\boldsymbol{\rho}$ of disaccharide proportions over positions $i = 2$ to n and disaccharide proportions $\boldsymbol{\gamma}$ at the non-reducing end, H&C builds one BKHS chain via the following random experiment.

(Random experiment R2).
Randomly draw disaccharide d_1 at position 1 based on $\boldsymbol{\gamma}$. Randomly draw disaccharide d_2 at position 2 based on $\boldsymbol{\rho}$. For positions $i = 3$ to n, randomly draw d_i based on transition probabilities P_{ad}, where a is the disaccharide at position $i-1$.

Under **R2** the relative abundance of species $s = d_1 \ldots d_n$ is given by:

$$p_s = \gamma(d_1)\,\rho(d_2) \prod_{i=3}^{n} P_{d_{i-1},d_i}. \tag{2.9}$$

Because $\mathbf{P}$ satisfies balance equations, disaccharide composition at any position i between 2 and n is composition $\boldsymbol{\rho}$.

2.3.2 Model of digest

Digestion by a *heparinase* is again modeled by a random experiment.

(Random experiment R2C).
Generate one BKHS chain with random experiment **R2**. *For positions* $i = 2$ *to* n*, randomly cleave this chain on the left of disaccharide* d_i *with probability* $c(d_i)$ *corresponding to the enzyme yield.*

As was obtained for model H&I, under **R2C** the probability of observing cleavage at any position $i \geq 2$ over all chains created by **R2** is

$$c = \sum_{d \in \mathcal{D}} c(d)\, \rho(d)\, .$$

However, with **R2C** cleavages and absences of cleavage along one particular BKHS chain are no longer independent events. This means that one must utilize conditional probabilities. For instance, probability $\Pr(\mathcal{C}_{i+1} \mid \mathcal{C}_i)$ of cleavage at position $i+1$ given cleavage at i is

$$\begin{aligned} c_+^{|+} &= \frac{\Pr(\mathcal{C}_i \wedge \mathcal{C}_{i+1})}{\Pr(\mathcal{C}_i)} \\ &= \frac{1}{c} \sum_{a,b} \rho(a)\, c(a)\, c(b)\, P_{ab}. \end{aligned}$$

Next, distribution of fragment length L is derived via the following ratios:

$$g(l) = \frac{\beta(l)}{\alpha} \quad [1 \leq l \leq n-1],$$
$\beta(l)$: expected number of fragments of length l under **R2C**,
α : expected total number of fragments under **R2C**.

Note that $l = n$ is not considered since only fragments generated by cleavages are experimentally quantified. This implies that the expected number α of fragments is the expected number of cleavages

$$\alpha = (n-1)\, c.$$

To calculate β, four cases are considered separately: $l = 1$, $l = 2$, $3 \leq l \leq n-2$ and $l = n-1$. Let L_i be the random variable "length of a fragment originating from position i in a BKHS chain". Then, under **R2C** one has

$$\Pr(L_i = 1) = \begin{cases} c_+^{|+} & [2 \leq i \leq n-1] \\ 1 & [i = n] \end{cases} .$$

This yields the expected number of fragments of length 1:

$$\begin{aligned} \beta(1) &= \sum_{i=2}^{n} \Pr(\mathcal{C}_i \wedge (L = 1)) \\ &= c\left(1 + (n-2)\, c_+^{|+}\right). \end{aligned}$$

The ratio $\beta(1)/\alpha$ gives the proportion of fragments of length 1:

$$g(1) = \frac{1 + (n-2)\, c_{+}^{|+}}{n-1}.$$

Next, for $l = 2$ and $i \leq n-2$ one has

$$\begin{aligned}
\Pr(\mathcal{C}_i \wedge (L=2)) &= \Pr\left(\mathcal{C}_i \wedge \overline{\mathcal{C}_{i+1}} \wedge \mathbf{C}_{i+2}\right) \\
&= \sum_{u,v,w} \rho(u)\, c(u)\, P_{uv}\, (1 - c(v))\, P_{vw} c(w) \\
&= \sum_{u} \rho(u)\, c(u) \sum_{v} \pi_{uv}^{(1)} \sum_{w} P_{vw} c(w),
\end{aligned}$$

where

$$\pi_{uv}^{(1)} = P_{uv}\, (1 - c(v))$$

is the probability of transitioning from u to v in one step and without cleavage. For $i = n-1$, the probability of cleavage at $i+2$ is 1 so that

$$\Pr(\mathcal{C}_i \wedge (L=2)) = \sum_{u} \rho(u)\, c(u) \sum_{v} \pi_{uv}^{(1)}.$$

Therefore, the expected number of fragments of length 2 is given by

$$\begin{aligned}
\beta(2) &= \sum_{i=2}^{n-1} \Pr(\mathcal{C}_i \wedge (L=2)) \\
&= \sum_{u} \rho(u)\, c(u) \sum_{v} \pi_{uv}^{(1)} \left(1 + (n-3) \sum_{w} P_{vw} c(w)\right).
\end{aligned}$$

The ratio $\beta(2)/\alpha$ yields the expected proportion of fragments of length 2:

$$g(2) = \frac{1}{(n-1)\, c} \sum_{u} \rho(u)\, c(u) \sum_{v} \pi_{uv}^{(1)} \left(1 + (n-3) \sum_{w} P_{vw} c(w)\right).$$

For $3 \leq l \leq n-2$, one obtains $g(l)$ by generalizing probabilities $\pi_{uv}^{(1)}$ to transitions without cleavage over more than one residue. Let $\pi_{uv}^{(2)}$ be the probability of transition from u to v without cleavage and in two steps. This probability is calculated by considering all possible sequences uav:

$$\begin{aligned}
\pi_{uv}^{(2)} &= \sum_{a} P_{ua}\, (1 - c(a))\, P_{av}\, (1 - c(v)) \\
&= \sum_{a} \pi_{ua}^{(1)} \pi_{av}^{(1)}.
\end{aligned}$$

This shows that matrix $\mathbf{\Pi}^{(2)}$ of transition probabilities in two steps is the square of matrix $\mathbf{\Pi}^{(1)}$. Consequently, induction yields

$$\mathbf{\Pi}^{(l+1)} = \mathbf{\Pi}^{(l)} \times \mathbf{\Pi}^{(1)}.$$

This means that computing all matrices $\mathbf{\Pi}$ can be performed in $O\left(nm^3\right)$. After computing the above matrices one obtains the expected number of fragments of length l:

$$\beta\left(l\right)=\sum_{u}\rho\left(u\right)c\left(u\right)\sum_{v}\pi_{uv}^{\left(l-1\right)}\left(1+\left(n-l-1\right)\sum_{w}P_{vw}c\left(w\right)\right).$$

The ratio $\beta\left(l\right)/\alpha$ yields the proportion of fragments of length l

$$g\left(l\right)=\frac{1}{\left(n-1\right)c}\sum_{u}\rho\left(u\right)c\left(u\right)\sum_{v}\pi_{uv}^{\left(l-1\right)}\left(1+\left(n-l-1\right)\sum_{w}P_{vw}c\left(w\right)\right)\quad\left[2\leq l\leq n-2\right].$$

Finally, the longest fragment of length $l=n-1$ corresponds to cleavage at position 2 and absence of cleavage over the remaining $n-2$ bonds:

$$g\left(n-1\right)=\frac{1}{\left(n-1\right)c}\sum_{u}\rho\left(u\right)c\left(u\right)\sum_{v}\pi_{uv}^{\left(n-2\right)}.$$

Because this last expression fits into that for $2\leq l\leq n-2$, distribution g of fragment length under H&C is summarized with

$$\begin{aligned}
&g\left(1\right)=\frac{1+\left(n-2\right)c_{+}^{|+}}{n-1}\quad\text{with}\quad c_{+}^{|+}=\frac{1}{c}\sum_{a,b}\rho\left(a\right)c\left(a\right)c\left(b\right)P_{ab}\\
&g\left(l\right)=\frac{1}{\left(n-1\right)c}\sum_{u}\rho\left(u\right)c\left(u\right)\sum_{v}\pi_{uv}^{\left(l-1\right)}\left(1+\left(n-l-1\right)\sum_{w}P_{vw}c\left(w\right)\right)\quad\left[2\leq l\leq n-1\right]\\
&\quad\text{with}\quad\pi_{uv}^{\left(1\right)}=P_{uv}\left(1-c\left(v\right)\right)\quad\text{and}\quad\mathbf{\Pi}^{\left(l+1\right)}=\mathbf{\Pi}^{\left(l\right)}\times\mathbf{\Pi}^{\left(1\right)}.
\end{aligned}\tag{2.10}$$

This shows that computing matrices $\mathbf{\Pi}$ and then g is only $O\left(nm^3\right)$.

2.3.3 Estimation of model parameters

Let $f_z\left(l\right)$ be an observed distribution of fragment length for *heparinase* z, with the convention that, for an upper bound l_z, $f_z\left(l_z\right)$ is the relative abundance of fragments of length l_z or greater. The same convention is utilized for theoretical distribution g_z, i.e. $g_z\left(l_z\right)$ is actually the sum of $g_z\left(l\right)$ values for $l=l_z$ to $n-1$. Then, solving the constrained optimization problem

$$\begin{aligned}
&\text{minimize w.r.t. }\mathbf{P}\qquad E\left(\mathbf{P}\right)=\sum_{z}\sum_{l=1}^{l_z}\left|f_z\left(l\right)-g_z\left(l,\mathbf{P}\right)\right|,\\
&\text{subject to}\qquad P_{ab}\geq 0,\ \sum_{b}P_{ab}=1,\ \rho\left(b\right)=\sum_{a}\rho\left(a\right)P_{ab},
\end{aligned}\tag{2.11}$$

means finding a homogeneous Markov model that best reproduces the observed distributions f_z of fragment length. Constraints on matrix $\mathbf{P}$ are enforced via projection. Briefly, matrix $\mathbf{P}$ is first represented as a vector

$$\begin{aligned}
\mathbf{p}&=\left(P_{11},P_{12},\ldots,P_{1m},P_{21},\ldots,P_{mm}\right)^T\\
&=\left(p_1,\ldots,p_k,\ldots,p_{m^2}\right)^T.
\end{aligned}$$

Indices i, j and k of variables P_{ij} and p_k are related by

$$\begin{array}{lcl} k & = & j + (i-1)\,m, \\ i\,(k) & = & 1 + (k-1) \div m, \\ j\,(k) & = & k - (i\,(k) - 1)\,m, \end{array}$$

where $\div$ stands for integer division. With this notation, non-negativity, balance equation, and stochastic matrix constraints are represented as linear inequality constraints on vector $\mathbf{p}$ of species abundances:

$$\mathbf{A}\mathbf{p} \le \mathbf{b}.$$

Entries A_{rk} of the $m\,(m+4) \times m^2$ matrix $\mathbf{A}$ and components b_r of vector $\mathbf{b}$ are as follows

row r	A_{rk}	b_r
$[\,1 \le r \le m^2\,]$	$-\delta\,(r,k)$	0
$[\,m^2+1 \le r \le m^2+m\,]$	$\delta\,(j\,(k)\,, r-m^2)\,\rho_{i(k)}$	ρ_{r-m^2}
$[\,m^2+m+1 \le r \le m^2+2m\,]$	$-\delta\,(j\,(k)\,, r-m^2-m)\,\rho_{i(k)}$	$-\rho_{r-m^2-m}$
$[\,m^2+2m+1 \le r \le m^2+3m\,]$	$\delta\,(i\,(k)\,, r-m^2-2m)$	1
$[\,m^2+3m+1 \le r \le m^2+4m\,]$	$-\delta\,(i\,(k)\,, r-m^2-3m)$	-1

with $\delta\,(i,j) = 1$ if $i = j$ and 0 otherwise. Given $\mathbf{A}$, $\mathbf{b}$ and any vector $\mathbf{y}$ of dimension m^2 one can find $\mathbf{p}$ which is closest to $\mathbf{y}$ and satisfies $\mathbf{A}\mathbf{p} \le \mathbf{b}$ by projection:

$$\text{minimize} \quad \|\mathbf{y} - \mathbf{p}\|_2 \quad \text{subject to} \quad \mathbf{A}\mathbf{p} \le \mathbf{b}.$$

This inequality-constrained convex quadratic program is solved via its dual:

$$\text{minimize} \quad \frac{1}{2}\mathbf{z}^T\mathbf{B}\mathbf{z} + \mathbf{t}^T\mathbf{z} \quad \text{subject to} \quad \mathbf{z} \ge \mathbf{0} \quad \text{with} \quad \mathbf{B} = \frac{1}{2}\mathbf{A}\mathbf{A}^T \quad \text{and} \quad \mathbf{t} = \mathbf{b} - \mathbf{A}\mathbf{y}.$$

The dual problem is solved by coordinate descent [4]. Namely, starting from a random point $\mathbf{z} > \mathbf{0}$, the cost is minimized one component z_j at a time according to

$$z_j \leftarrow \max\left(0, z_j - \frac{1}{b_{jj}}\left(t_j + \sum_k b_{jk} z_k\right)\right).$$

A cyclic sequence $j = 1, 2, \ldots, 1, 2, \ldots$ is utilized until the cost does not change significantly. Once solution $\mathbf{z}$ has been computed, solution to the primal problem is given by

$$\mathbf{p} = \mathbf{y} - \frac{1}{2}\mathbf{A}^T\mathbf{z}.$$

This projection technique makes it possible to find approximate solutions to (2.11) with simulated annealing [5].

Due to its stochastic nature, simulated annealing is not guaranteed to yield the same solution when started with different initial conditions. Figure S3 shows that errors of fit E

Supplementary Figure S3: Cumulative distribution of error of fit E for model H&C over 100 runs of parameter fitting via simulated annealing started with different initial conditions.

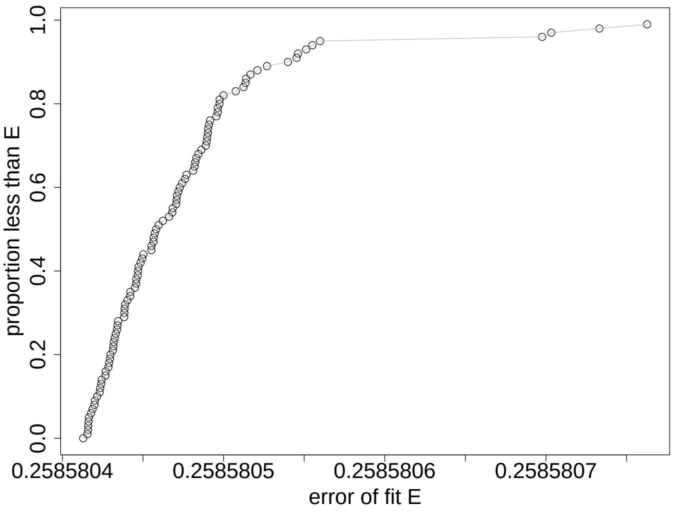

(final values of the objective function in (2.11)) show little variance over 100 runs of simulated annealing started with different initial conditions. It is therefore likely that simulated annealing provides a reasonable approximation of the best possible fit for model H&C.

Lastly for model H&C, figure S4 shows results obtained for different BKHS chain lengths n. While increasing chain length n yields slightly better fit to heparinase digest data, error of fit is still large ($E > 0.2$) even for n as large as 20. These results are in agreement with the hypothesis that model H&C is unable to reproduce heparinase digest data.

Supplementary Figure S4: Errors of fit E for model H&C as a function of BKHS chain length n. Statistics on E are shown over 20 runs of simulated annealing started with different initial conditions.

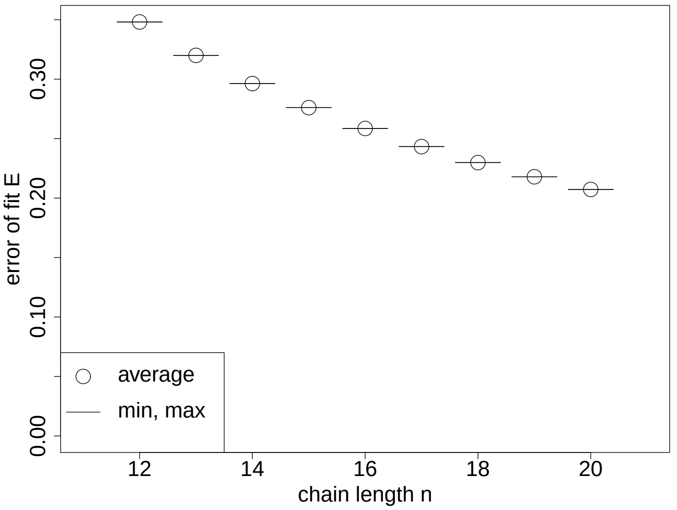

2.4 Nonhomogeneity and independence

2.4.1 Model of species abundances

The previous two models where homogeneous: over all species, disaccharide composition is the same at each position i from the non-reducing end. Nonhomogeneity and Independence model N&I departs from homogeneity. The parameter of N&I is a $n \times m$ matrix:

$$\mathbf{\Gamma} = \begin{bmatrix} \Gamma_{11} & \dots & \Gamma_{1m} \\ \Gamma_{21} & \dots & \Gamma_{2m} \\ \vdots & \ddots & \vdots \\ \Gamma_{n1} & \dots & \Gamma_{nm} \end{bmatrix} \tag{2.12}$$

where Γ_{ij} is the proportion of disaccharide j among all disaccharides at position i from the non-reducing end. To simplify notation, letter d will sometimes be utilized instead of j:

$$\Gamma_{ij} = \Gamma_i\left(d\right) \quad \text{and} \quad \rho_j = \rho\left(d\right),$$

where vector $\boldsymbol{\rho}$ represents overall proportions of disaccharides over positions $i = 2$ to n. Because N&I should preserve $\boldsymbol{\rho}$ and proportions at each position should sum to 1, matrix $\mathbf{\Gamma}$ is subject to the following constraints:

$$\begin{aligned} &\frac{1}{n-1}\sum_{i=2}^{n}\Gamma_{ij} = \rho_j, \\ &\sum_{j}\Gamma_{ij} = 1, \\ &\Gamma_{ij} \geq 0. \end{aligned} \tag{2.13}$$

Given a matrix $\mathbf{\Gamma}$, chains of BKHS are generated as follows.

(**Random experiment R3).**
For each position $i = 1$ to n, randomly choose disaccharide d_i at position i based on vector $\mathbf{\Gamma_{i\cdot}}$ of disaccharide proportions.

In other words, the relative abundance of species $s = d_1 \dots d_n$ is

$$p_s = \prod_{i=1}^{n}\Gamma_i\left(d_i\right). \tag{2.14}$$

These abundances preserve overall composition $\boldsymbol{\rho}$, when matrix $\mathbf{\Gamma}$ satisfies (2.13). Composition $\mathbf{\Gamma_{1\cdot}}$ at the non-reducing end will not intervene in the calculation of fragment length distribution. Like H&I, N&I is an independence model, in the sense that for one chain the likelihood of a residue at position i does not depend on the residue at position $i-1$, but only on $\mathbf{\Gamma_{i\cdot}}$. However, species abundances can be very different with N&I than they are with H&I. Finally, it is mentioned that equation (2.14) corresponds to a maximum-entropy model of species abundances, this model being defined by matrix $\mathbf{\Gamma}$.

2.4.2 Model of digest

As before, digestion of BKHS chains generated in N&I is modeled with a random experiment.

(Random experiment R3C).
Generate one BKHS chain with random experiment **R3**. *For positions $i = 2$ to n, randomly cleave this chain on the left of residue d_i with probability $c(d_i)$ corresponding to the enzyme yield.*

Unlike with models H&I and H&C, with N&I the probability of observing a cleavage depends on position i:

$$c_i = \sum_{d \in \mathcal{D}} c(d)\, \Gamma_i(d) \qquad [\,2 \le i \le n\,]. \tag{2.15}$$

Yet, to simplify notations, it will be useful to utilize again the quantity

$$c = \sum_{d \in \mathcal{D}} c(d)\, \rho(d). \tag{2.16}$$

As was done for H&C, the distribution of fragment length for N&I is calculated by deriving the following ratio:

$$g(l) = \frac{\beta(l)}{\alpha} \quad [\,1 \le l \le n-1\,],$$
$\beta(l)$: expected number of fragments of length l under **R3C**,
α : expected total number of fragments under **R3C**.

Given that fragments originating from position $i = 1$ are not experimentally detected, the expected total number of fragments is the expected number of cleavages:

$$\alpha = \sum_{i=2}^{n} c_i.$$

Combining this with equations (2.13) and (2.16) yields

$$\alpha = (n-1)\, c. \tag{2.17}$$

Next, expected numbers $\beta(l)$ of fragments of length l and proportions $g(l)$ are derived by separately treating cases $l = 1$, $l = 2$, $3 \le l \le n-2$ and $l = n-1$.
For $l = 1$ one has

$$\begin{aligned} g(1) &= \frac{1}{\alpha} \sum_{i=2}^{n} \Pr\left(\mathcal{C}_i \wedge \mathcal{C}_{i+1}\right) \\ &= \frac{c_n}{\alpha} + \frac{1}{\alpha} \sum_{i=2}^{n-1} c_i c_{i+1}. \end{aligned}$$

Next, for $l = 2$, cases $i \le n-2$ and $i = n-1$ are treated separately. For $i \le n-2$ one has

$$\begin{aligned} \Pr\left(\mathcal{C}_i \wedge (L=2)\right) &= \Pr\left(\mathcal{C}_i \wedge \overline{\mathcal{C}}_{i+1} \wedge \mathcal{C}_{i+2}\right) \\ &= \sum_{u,v,w} \Gamma_i(u)\, c(u)\, \Gamma_{i+1}(v)\, (1 - c(v))\, \Gamma_{i+2}(w)\, c(w) \\ &= c_i c_{i+2} \sum_{v} \pi_i^{(1)}(v). \end{aligned}$$

Quantity $\pi_i^{(1)}(v)$ is the probability of transitioning from any disaccharide at position i to v in one step and without cleavage. It is given by

$$\begin{aligned}\pi_i^{(1)}(v) &= \Gamma_{i+1}(v)\,(1-c(v)) \\ &= 1-c_{i+1}.\end{aligned}$$

For $i=n-1$, there is always cleavage at $n+1$ so that

$$\Pr\left(\mathcal{C}_{n-1}\wedge(L=2)\right)=c_{n-1}\,(1-c_n)\,.$$

Therefore, the proportion of fragments of length 2 is

$$g(2)=\frac{c_{n-1}\,(1-c_n)}{\alpha}+\sum_{i=2}^{n-2}\frac{c_i\,(1-c_{i+1})\,c_{i+2}}{\alpha}.$$

Next, the above expression of $g(2)$ is generalized to $3\le l\le n-2$, first for the case $i\le n-l$:

$$\Pr\left(\mathcal{C}_i\wedge(L=l)\right)=c_ic_{i+l}\sum_v\pi_i^{(l-1)}(v)\,,$$

where $\pi^{(l-1)}(v)$ is the probability of transitioning from any disaccharide at position i to disaccharide v in $l-1$ steps and without any cleavage. Consider first that $l=3$:

$$\begin{aligned}\pi_i^{(2)}(v) &= \Gamma_{i+2}(v)\,(1-c(v))\sum_a\Gamma_{i+1}(a)\,(1-c(a)) \\ &= \Gamma_{i+2}(v)\,(1-c(v))\,(1-c_{i+1})\,.\end{aligned}$$

From this one obtains

$$\sum_v\pi_i^{(2)}(v)=(1-c_{i+1})\,(1-c_{i+2})\,.$$

Therefore, induction yields

$$\sum_v\pi_i^{(l-1)}(v)=\prod_{j=i+1}^{i+l-1}(1-c_j)\,,$$

which in turn implies

$$\Pr\left(\mathcal{C}_i\wedge(L=l)\right)=c_ic_{i+l}\prod_{j=i+1}^{i+l-1}(1-c_j)\,.$$

For $i=n-l+1$, given that $c_{i+l}=1$, this simplifies to

$$\Pr\left(\mathcal{C}_{n-l+1}\wedge(L=l)\right)=c_{n-l+1}\prod_{j=n-l+2}^{n}(1-c_j)\,.$$

Combining the last two equations and dividing by the expected total number of fragments α, one obtains the proportion of fragments of length l:

$$\begin{aligned}g(l) &= \frac{1}{\alpha}\sum_{i=2}^{n-l+1}\Pr\left(\mathcal{C}_i\wedge(L=l)\right) \\ &= \frac{c_{n-l+1}}{\alpha}\prod_{j=n-l+2}^{n}(1-c_j)+\sum_{i=2}^{n-l}\frac{c_ic_{i+l}}{\alpha}\prod_{j=i+1}^{i+l-1}(1-c_j)\,.\end{aligned}$$

Lastly, the expected number $\beta(n-1)$ of fragments of length $n-1$ corresponds to probability of cleavage at position 2 and no cleavage over the other positions. Dividing by α yields

$$g(n-1) = \frac{c_2}{\alpha} \prod_{j=3}^{n} (1 - c_j).$$

Distribution of fragment length under model N&I is summarized as follows.

$$\begin{aligned}
&g(1) = \frac{c_n}{\alpha} + \frac{1}{\alpha} \sum_{i=2}^{n-1} c_i c_{i+1} \\
&g(l) = \frac{c_{n-l+1}}{\alpha} \omega(n-l+2, n) + \sum_{i=2}^{n-l} \frac{c_i c_{i+l}}{\alpha} \omega(i+1, i+l-1) \quad [2 \leq l \leq n-2] \\
&g(n-1) = \frac{c_2}{\alpha} \omega(3, n) \\
&\text{with} \\
&\omega(i,j) = \prod_{k=i}^{j} (1 - c_k) \quad [3 \leq i < j \leq n], \\
&\alpha = (n-1)c, \quad c = \sum_b c(b)\rho(b) \quad \text{and} \quad c_i = \sum_b c(b)\Gamma_i(b) \quad [2 \leq i \leq n].
\end{aligned} \tag{2.18}$$

Because quantities $\omega(i,j)$ can be computed in $O(n^2)$, computing all values of $g(l)$ can be performed in $O(n^2)$, after having computed all values of c_i which is only $O(mn)$.

2.4.3 Estimation of model parameters

Let $f_z(l)$ stand again for an experimentally measured distribution of fragment length l after digestion by enzyme z, with the convention that, for some upper bound l_z, $f(l_z)$ is the relative abundance of fragments of length equal to or greater than l_z. Likewise, $g_z(l_z)$ stands for the sum of $g_z(l)$ values for l between l_z and $n-1$. Parameter $\mathbf{\Gamma}$ of model N&I is estimated by solving the following constrained optimization problem

$$\begin{aligned}
&\text{minimize w.r.t. } \mathbf{\Gamma} \qquad E(\mathbf{\Gamma}) = \sum_z \sum_{l=1}^{l_z} |f_z(l) - g_z(l, \mathbf{\Gamma})|, \\
&\text{subject to} \qquad \sum_{i=2}^{n} \Gamma_{ij} = (n-1)\rho_j, \quad \sum_j \Gamma_{ij} = 1 \quad \text{and} \quad \Gamma_{ij} \geq 0.
\end{aligned} \tag{2.19}$$

Approximate solutions to this problem are obtained via simulated annealing [5]. Constraints on $\mathbf{\Gamma}$ are enforced via projection. More precisely, let $\boldsymbol{\gamma}$ be the following vector representation of $\mathbf{\Gamma}$ obtained by stacking its columns:

$$\begin{aligned}
\boldsymbol{\gamma} &= (\Gamma_{11}, \ldots, \Gamma_{n1}, \Gamma_{12}, \ldots, \Gamma_{n2}, \ldots, \Gamma_{nm})^T \\
&= (\gamma_1, \ldots, \gamma_k, \ldots, \gamma_{n \times m})^T.
\end{aligned}$$

That is, if $\div$ represents integer division, indices i, j and k are related by

$$\begin{aligned}
k &= (j-1)n + i, \\
j(k) &= 1 + (k-1) \div n, \\
i(k) &= k - (j(k) - 1)n.
\end{aligned}$$

With these notations, constraints on matrix $\mathbf{\Gamma}$ are represented as linear inequality constraints on vector $\boldsymbol{\gamma}$: $\mathbf{A}\boldsymbol{\gamma} \leq \mathbf{b}$. Entries A_{rk} of the $(nm + 2m + 2n) \times nm$ matrix $\mathbf{A}$ and components b_r of vector $\mathbf{b}$ are given by

row r	A_{rk}	b_r
$[1 \leq r \leq nm]$	$-\delta(r,k)$	0
$[nm+1 \leq r \leq nm+m]$	$\dfrac{\delta(j(k), r-nm)}{n-1}(1-\delta(i(k),1))$	ρ_{r-nm}
$[nm+m+1 \leq r \leq nm+2m]$	$-\dfrac{\delta(j(k), r-nm-m)}{n-1}(1-\delta(i(k),1))$	$-\rho_{r-nm-m}$
$[nm+2m+1 \leq r \leq nm+2m+n]$	$\delta(i(k), r-nm-2m)$	1
$[nm+2m+n+1 \leq r \leq nm+2m+2n]$	$-\delta(i(k), r-nm-2m-n)$	-1

where $\delta(i,j) = 1$ if $i = j$ and 0 otherwise. If a constraint for composition $\boldsymbol{\zeta}$ at the reducing end $(i = n)$ is also included, a $2(m-1) \times nm$ matrix $\mathbf{B}$ is appended to $\mathbf{A}$ and a vector $\mathbf{d}$ is appended to $\mathbf{b}$:

row r	B_{rk}	d_r
$[1 \leq r \leq m-1]$	$\delta(i(k),n)\,\delta(j(k),r)$	ζ_r
$[m \leq r \leq 2m-2]$	$\delta(i(k),n)\,\delta(j(k),r-m+1)$	ζ_{r-m+1}

Supplementary Figure S5: Distributions of error of fit E over 50 runs of simulated annealing for model N&I when not constraining disaccharide composition at the reducing end (dots) and when setting the highest allowed proportion of S at the reducing end (crosses).

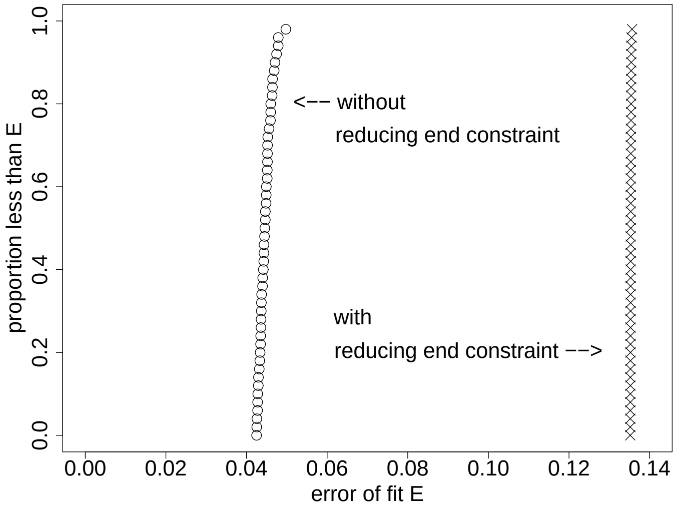

Given $\mathbf{A}$, $\mathbf{b}$ and any vector $\mathbf{y}$ of dimension nm, one can find vector $\boldsymbol{\gamma}$ which is closest to $\mathbf{y}$ and belongs to the polyhedral set defined by $\mathbf{A}\boldsymbol{\gamma} \leq \mathbf{b}$ via the projection technique already described in section 2.3.3. This technique makes it possible to find approximate solutions to (2.19) with simulated annealing.

Simulated annealing is utilized because problem (2.19) might have local minima. Dots in figure S5 show the distribution of final values of E over fifty runs of simulated annealing,

Supplementary Figure S6: Errors of fit E for model N&I as a function of BKHS chain length n. Statistics on E are shown over 10 runs of simulated annealing started with different initial conditions.

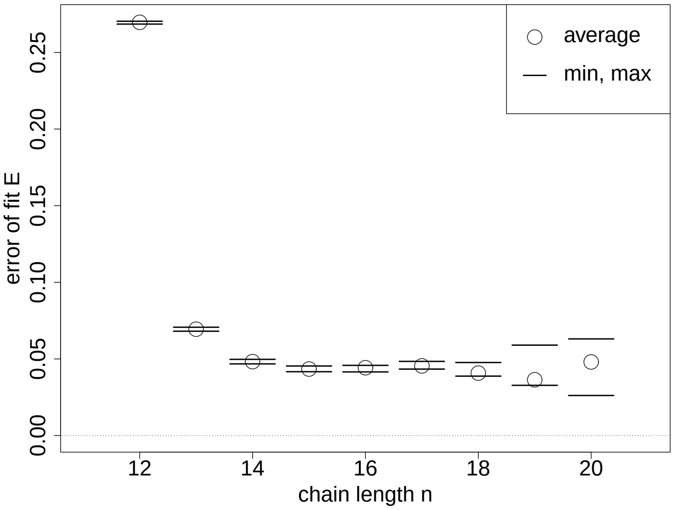

when not constraining proportion of sulfated disaccharides S at the reducing end. Because the coefficient of variation of E between runs is small ($\sim 3.6\%$), it is likely that simulated annealing provides approximate optimal values of $\mathbf{\Gamma}$.

Final values of E are clearly greater than 0. In addition, optimized matrices $\mathbf{\Gamma}$ are all such that proportion of sulfated disaccharides S at the reducing end is large ($\sim 75\%$). Such a high proportion is actually incompatible with experimental data: linear programming shows that the highest possible proportion of S at the reducing end is $\sim$56%. Adding this additional constraint to optimization problem (2.19) yields errors of fit E which are now about three times larger, as shown by crosses in figure S5. These results are in agreement with the hypothesis that model N&I cannot reproduce experimental data of *heparinase* digests.

Lastly, figure S6 shows that lack of fit observed with BKHS chain length $n = 16$ is also obtained for different values of n.

3 Non-parametric modeling

3.1 Constraints on species abundances

Experimental measurements for overall disaccharide composition and distributions of fragment length in *heparinase* digests can be formalized as linear equality constraints on vector $\mathbf{p}$ of individual species abundances: $\mathbf{Ap} = \mathbf{b}$, where $\mathbf{A}$ is a matrix and $\mathbf{b}$ a vector of measurements. For overall proportion of disaccharide j over positions $i = 2$ to n, one has

$$b_j = \rho_j \quad \text{and} \quad A_{js} = \frac{r(s,j)}{n-1}, \tag{3.1}$$

where $r(s,j)$ is the number of occurrences of disaccharide j in the sequence of species s between positions $i = 2$ and n. Call $f_z(l)$ the relative abundance of fragments of length l

after digestion by enzyme z. The corresponding linear constraint is given by

$$b_l = f_z(l) \quad \text{and} \quad A_{ls} = \frac{q(s,l)}{(n-1)c} \quad \text{with} \quad c = \sum_d c_z(d)\,\rho(d) \tag{3.2}$$

and where $q(s,l)$ is the expected number of fragments of length l when randomly cleaving species s based on cleavage specificities $c_z(d)$ for each disaccharide d. Call $d_1 d_2 \ldots d_n$ the sequence of species s. Expressions of $q(s,l)$ are derived separately for $l = n$, $l = n-1$, $l = 1$ and $2 \leq l \leq n-2$.

Case $l = n$ corresponds to no cleavage and the resulting "fragment" (intact chain) cannot be experimentally detected, so that $q(s,n) = 0$.

Case $l = n-1$ corresponds to one cleavage at position 2 and no cleavage at other positions

$$\begin{aligned} q(s,n-1) &= c_z(d_2)\prod_{j=3}^{n}\left(1 - c_z(d_j)\right), \\ &= c_z(d_2)\prod_{j=1}^{n-2}\left(1 - c_z(d_{j+2})\right). \end{aligned}$$

Case $l = 1$ corresponds to either one cleavage at n or two successive cleavages at any position i such that $2 \leq i \leq n-1$:

$$q(s,1) = \sum_{i=2}^{n-1} c_z(d_i)\,c_z(d_{i+1}) + c_z(d_n).$$

Case $2 \leq l \leq n-2$ corresponds to either one cleavage at position $n-l$ followed by no cleavage at higher positions or two cleavages separated by $l-1$ absences of cleavage:

$$q(s,l) = \sum_{i=2}^{n-l-1} c_z(d_i)\,c_z(d_{i+l})\prod_{j=1}^{l-1}\left(1 - c_z(d_{i+j})\right) + c_z(d_{n-l})\prod_{j=n-l+1}^{n}\left(1 - c_z(d_{i+j})\right).$$

Finally, denominator $(n-1)c$ in (3.2) is the expected total number of fragments in the entire mixture. Since fragments are experimentally detected (UV absorbance at 232 nm) only when generated by a cleavage, the expected number of fragments is here the expected number of cleavages.

Specifying disaccharide composition $\boldsymbol{\gamma}$ at any position i from the non-reducing end yields linear constraints $\mathbf{Ap} = \mathbf{b}$ with

$$b_j = \gamma_j \quad \text{and} \quad A_{js} = \delta(s_i, j) \quad \text{with} \quad \delta(x,y) = \begin{cases} 1 & \text{if } x = y \\ 0 & \text{otherwise} \end{cases}. \tag{3.3}$$

That is, $A_{js} = 1$ if species s has disaccharide j at position i and 0 otherwise. Combining this type of constraint over positions $i = 2$ to n and setting $\boldsymbol{\gamma} = \boldsymbol{\rho}$ yields a constraint for homogeneity.

The above constraints correspond to a mixture in which all BKHS chains have same length n. Consider now a vector $\mathbf{n} = (n^-, \ldots, n^+)$ of chain lengths and $\mathbf{w}$ the vector of their relative

abundances (chain length distribution). Models of $\mathbf{w}$ were obtained by first generating a vector $\mathbf{x}$, where $x(n)$ is the integral between n and $n+1$ of a Gaussian density with mean μ and standard deviation σ, and then projecting $\mathbf{x}$ onto the polyhedral set defined by

$$\mathbf{w} \geq \mathbf{0}, \quad \mathbf{1}^T \mathbf{w} = 1 \quad \text{and} \quad \mathbf{n}^T \mathbf{w} = \mu.$$

The projection method is that described in section 2.3.3. Value $\mu = 16$ was chosen, since it is the experimentally determined average chain length in BKHS. Chain length distribution $\mathbf{w}$ translates into a linear equality constraint $\mathbf{A}\mathbf{p} = \mathbf{w}$, where for each species s and each chain length n:

$$A_{n,s} = \delta(n, \text{length}(s)) \quad \text{with} \quad \delta(x,y) = \begin{cases} 1 & \text{if } x = y \\ 0 & \text{otherwise} \end{cases}.$$

Overall disaccharide composition $\boldsymbol{\rho}$ between position $i = 2$ from the non-reducing end and reducing end of chains yields again a constraint $\mathbf{A}\mathbf{p} = \boldsymbol{\rho}$, where entries of $\mathbf{A}$ are given by equation (3.1) after replacing denominator $n-1$ with $\mu - 1$. Likewise, fragment length distribution $\mathbf{f}$ in a *heparinase* digest yields a constraint $\mathbf{A}\mathbf{p} = \mathbf{f}$, where entries of $\mathbf{A}$ are given by equation (3.2) after replacing denominator $(n-1)\,c$ with $(\mu - 1)\,c$.

3.2 Linear programming

All considered linear programs were treated in the form

$$\begin{array}{ll} \text{minimize w.r.t. } \mathbf{p} & \mathbf{c}^T \mathbf{p}, \\ \text{subject to} & \mathbf{A}\mathbf{p} = \mathbf{b} \quad \text{and} \quad \mathbf{p} \geq \mathbf{0} \end{array}$$

and solved by an implementation of the simplex method similar to that described in [6].

3.3 Maximum-entropy modeling

Call $S(\mathbf{p})$ the entropy of a vector $\mathbf{p}$ of species abundances:

$$S(\mathbf{p}) = -\sum_s p_s \ln p_s.$$

Maximum-entropy modeling was performed by solving the problem

$$\begin{array}{ll} \text{minimize w.r.t. } \mathbf{p} & -S(\mathbf{p}), \\ \text{subject to} & \mathbf{A}\mathbf{p} = \mathbf{b} \quad \text{and} \quad \mathbf{1}^T \mathbf{p} = 1, \end{array} \tag{3.4}$$

where $\mathbf{A}\mathbf{p} = \mathbf{b}$ represents experimental constraints of overall disaccharide composition and *heparinase* digests, matrix $\mathbf{A}$ having full rank. The sum-to-1 constraint is singled out for special treatment. Nonnegativity of $\mathbf{p}$ is implied by the objective function. Problem (3.4) is convex and can be solved via its dual [1,2]. Briefly, the Lagrangian function is

$$L(\mathbf{p}, \alpha, \mathbf{x}) = -S(\mathbf{p}) + \alpha\left(\mathbf{1}^T \mathbf{p} - 1\right) + \mathbf{x}^T(\mathbf{A}\mathbf{p} - \mathbf{b}).$$

Utilizing the conjugate function of entropy S one can show that the dual problem is

$$\text{minimize w.r.t. } \alpha, \mathbf{x} \quad \mathbf{x}^T\mathbf{b} + \alpha + e^{-(1+\alpha)} \sum_s \exp\left(-\mathbf{A_{.s}}^T\mathbf{x}\right),$$

where $\mathbf{A_{.s}}$ stands for the sth column of $\mathbf{A}$. Since there are no constraints on the Lagrange multipliers, the optimal α is obtained by setting the derivative to zero and this yields a simplified form of the dual:

$$\begin{array}{ll} \text{minimize w.r.t. } \mathbf{x} & \mathbf{\Gamma}(\mathbf{x}) = \mathbf{x}^T\mathbf{b} + \ln \mathbf{Z} \\ \text{with} & Z = \sum_s \exp\left(-\mathbf{A_{.s}}^T\mathbf{x}\right). \end{array} \tag{3.5}$$

This geometric program in convex form can be numerically solved [2,7] and optimal $\mathbf{x}^*$ yields maximum entropy model $\mathbf{p}^*$ by setting $\partial L/\partial p_s$ to zero for each species s:

$$p_s^* = \exp\left(-\mathbf{A_{.s}}^T\mathbf{x}^*\right)/Z. \tag{3.6}$$

Unconstrained problem (3.5) was solved via Newton's method, which requires computing gradient and Hessian of $\mathbf{\Gamma}$:

$$\frac{\partial \mathbf{\Gamma}}{\partial x_i} = b_i + \frac{1}{Z}\frac{\partial Z}{\partial x_i}, \quad \frac{\partial^2 \mathbf{\Gamma}}{\partial x_i \partial x_j} = \frac{1}{Z}\left(\frac{\partial^2 Z}{\partial x_i \partial x_j} - \frac{1}{Z}\frac{\partial Z}{\partial x_i}\frac{\partial Z}{\partial x_j}\right),$$
$$\frac{\partial Z}{\partial x_i} = -\sum_s A_{is}\exp\left(-\mathbf{A_{.s}}^T\mathbf{x}\right), \quad \frac{\partial^2 Z}{\partial x_i \partial x_j} = \sum_s A_{is}A_{js}\exp\left(-\mathbf{A_{.s}}^T\mathbf{x}\right).$$

The Hessian can become near-singular close to the optimum, therefore a modified Cholesky factorization was utilized for computing the Newton step. Line search was performed according to the Armijo rule.

Supplementary Figure S7: Distributions of relative abundances of molecular species for maximum-entropy models (chain length $n = 16$) with only overall disaccharide composition constraint (model H&I p') and with addition of *heparinase* digest constraints (model p^*).

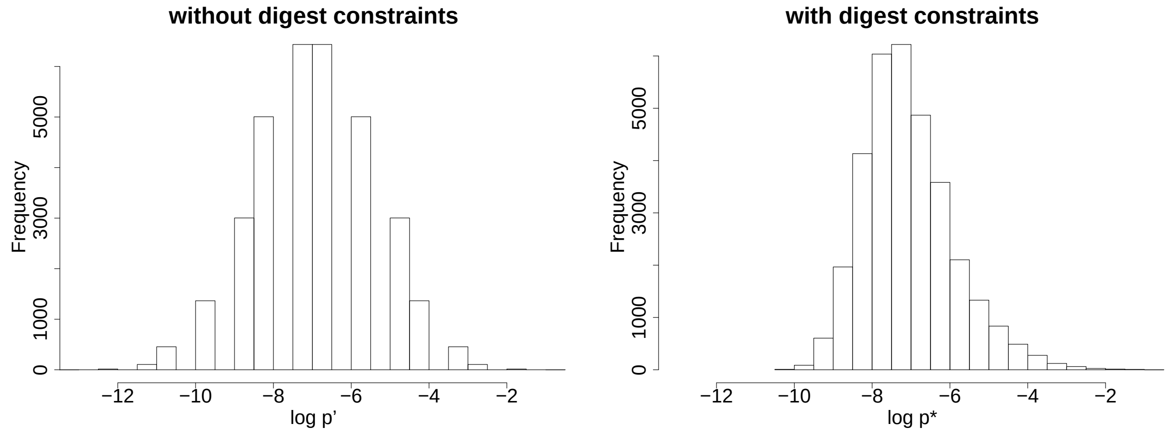

Figure S7 displays distributions of relative abundances of molecular species for two maximum-entropy models when all chains have same length $n = 16$ disaccharides. The first model $\mathbf{p}'$

Supplementary Table S3: Most abundant species under maximum-entropy model $\mathbf{p}^*$ and corresponding abundances $\mathbf{p}'$ without *heparinase* digest constraints (model H&I).

species	p^*	p'	p^*/p'
xUUUUUUUUUUUUUUU	1.2E-01	1.1E-01	1.1
x**S**UUUUUUUUUUUUUU	6.0E-02	1.8E-02	3.4
xUUUUUUUUUUUUUU**S**	4.2E-02	1.8E-02	2.4
xU**S**UUUUUUUUUUUUU	3.9E-02	1.8E-02	2.2
xUU**S**UUUUUUUUUUUU	3.7E-02	1.8E-02	2.1
xUUU**S**UUUUUUUUUUU	3.4E-02	1.8E-02	2.0
x**SS**UUUUUUUUUUUUU	3.3E-02	2.8E-03	11.9
xUUUU**S**UUUUUUUUUU	3.2E-02	1.8E-02	1.8
x**S**UUUUUUUUUUUUU**S**	2.1E-02	2.8E-03	7.7
x**SSS**UUUUUUUUUUUU	1.8E-02	4.3E-04	40.7
xUUUUUUUUUUUUU**SS**	1.6E-02	2.8E-03	5.8
xU**SS**UUUUUUUUUUUU	1.5E-02	2.8E-03	5.4
xUU**SS**UUUUUUUUUUU	1.4E-02	2.8E-03	5.1
xU**S**UUUUUUUUUUUU**S**	1.4E-02	2.8E-03	5.0
xUUUUU**S**UUUUUUUUU	1.3E-02	1.8E-02	0.8
xUUU**SS**UUUUUUUUUU	1.3E-02	2.8E-03	4.8
xUU**S**UUUUUUUUUUU**S**	1.3E-02	2.8E-03	4.7
xUUUUUUU**S**UUUUUUU	1.2E-02	1.8E-02	0.7
xUUU**S**UUUUUUUUUU**S**	1.2E-02	2.8E-03	4.4
xUUUUUU**S**UUUUUUUU	1.2E-02	1.8E-02	0.7
x**SS**UUUUUUUUUUUU**S**	1.2E-02	4.3E-04	26.8
x**SSSS**UUUUUUUUUUU	9.5E-03	6.8E-05	138.6
x**S**UUUUUUUUUUUU**SS**	8.2E-03	4.3E-04	18.8
xUUUUUUUU**S**UUUUUU	7.9E-03	1.8E-02	0.5
xUUUUUUUUUUUU**SSS**	7.4E-03	4.3E-04	17.0
xU**SSS**UUUUUUUUUUU	6.9E-03	4.3E-04	15.8
xUU**SSS**UUUUUUUUUU	6.5E-03	4.3E-04	14.9
x**SSS**UUUUUUUUUUU**S**	6.3E-03	6.8E-05	91.6
xUUUUUUUUUUUUU**S**U	5.7E-03	1.8E-02	0.3
xUUUU**SS**UUUUUUUUU	5.5E-03	2.8E-03	2.0
xU**SS**UUUUUUUUUUU**S**	5.2E-03	4.3E-04	12.0
xU**S**UUUUUUUUUUU**SS**	5.2E-03	4.3E-04	12.0
xUUUU**S**UUUUUUUUU**S**	5.1E-03	2.8E-03	1.8
x**SSSSS**UUUUUUUUUU	5.0E-03	1.1E-05	468.2
xUU**S**UUUUUUUUUU**SS**	4.9E-03	4.3E-04	11.4
xUU**SS**UUUUUUUUUU**S**	4.9E-03	4.3E-04	11.4
xUUUUUU**SS**UUUUUUU	4.9E-03	2.8E-03	1.8
xUUUUUUUUUUUU**S**UU	4.9E-03	1.8E-02	0.3
xUUUUU**SS**UUUUUUUU	4.8E-03	2.8E-03	1.7
xUUUUUU**S**UUUUUUU**S**	4.6E-03	2.8E-03	1.7

is based only on overall disaccharide composition and corresponds to model H&I. Adding constraints for *heparinase* digests (model $\mathbf{p}^*$) yields a distribution with smaller variance.

Differences between models $\mathbf{p}^*$ and $\mathbf{p}'$ are further illustrated with table S3. The table displays the 40 most abundant species under $\mathbf{p}^*$ and their corresponding relative abundances under $\mathbf{p}'$. For both models the most abundant species is xU_{15}, where x stands for the disaccharide at the non-reducing end. Large abundance of xU_{15} is induced by $\rho(U) > 6\rho(S)$. Heparinase digest constraints yield overrepresentation of sequences having more S towards the non-reducing end, e.g. $p^*/p' > 468$ for xS_5U_{10}.

References

1. Boyd S, Vandenberghe L. Convex Optimization. Cambridge University Press; 2004.

2. Agmon N, Alhassid Y, Levine R. An algorithm for finding the distribution of maximal entropy. J Comput Phys. 1979;30:250–258.

3. Brémaud P. Markov Chains, Gibbs Fields, Monte Carlo Simulation, and Queues. Springer; 1999.

4. Bertsekas D. Nonlinear Programming. 2nd ed. Athena Scientific; 1999, 2004.

5. Kirkpatrick S, Gelatt C, Vecchi M. Optimization by Simulated Annealing. Science. 1983;220:671–680.

6. Luenberger D, Ye Y. Linear and Nonlinear Programming. 3rd ed. International Series in Operations Research and Management Science. Springer; 2008.

7. Boyd S, Kim SJ, Vandenberghe L, Hassibi A. A tutorial on geometric programming. Optim Eng. 2007;8:67–127.

8. R Development Core Team. R: A Language and Environment for Statistical Computing. Vienna, Austria; 2008. ISBN 3-900051-07-0, http://www.R-project.org.

4 Source code

Java code which makes it possible to reproduce all computational results presented in this supplementary material and the associated manuscript is listed next. The code was designed to be executed from a directory having two subfolders called `input` and `output`, though this can be changed by editing the code.

4.1 Input files

All utilized data were already presented in this supplementary material. In order to use the code to reproduce results, data should be organized into five ASCII tab-delimited files as follows.

distributions of fragment lengths in *heparinase* digests

file `hepI.f.txt`

length	abundance
1	0.4676
2	0.0711
3	0.0544
4	0.0361
5	0.0351
6	0.0307
7	0.0353
8	0.038
9	0.0321
10	0.0302
11	0.1694

file `hepIII.f.txt`

length	abundance
1	0.9211
2	0.0545
3	0.0164
4	0.0058
5	0.0018
6	0.0004

cleavage yields by *heparinases*

file `US.hepI.txt`

name	specificity
U	0.03
S	1.00

file `US.hepIII.txt`

name	abundance
U	1.000
S	0.033

disaccharide overall abundances, file `US.ab.txt`

name	abundance
U	0.864172835
S	0.135827165

example of N&I model, file `GammaExample.txt`

position	u	s
1	0.4806084931679921	0.5193915068320079
2	0.4239003669429147	0.5760996330570853
3	0.9608720311816147	0.03912796881838522
4	0.9999999989643918	1.0356081894813407E-9
5	0.8960135492035556	0.10398645079644438
6	0.9517357778451813	0.04826422215481873
7	0.9350944994883947	0.06490550051160504
8	0.9358337572852157	0.06416624271478436
9	0.9687032273942001	0.031296772605799876
10	0.9999999989670628	1.0329372357453792E-9
11	0.9985296913265761	0.0014703086734238965
12	0.9994775558514656	5.224441485343068E-4
13	0.9972070500947344	0.0027929499052654974
14	0.9965400888355378	0.003459911164462176
15	0.6493685352919306	0.35063146470806933
16	0.24931639632722233	0.7506836036727775

The last file (`GammaExample.txt`) is utilized for quality check of the code.

4.2 Summary of classes

Supplementary Table S4: Brief description of classes

class	description
`AVSFormCons`	Represents a linear constraint $\mathbf{Ax} = \mathbf{b}$ with $\mathbf{b} \geq \mathbf{0}$ and artificial variables (one for each row of $\mathbf{A}$)
`BBSet`	Set of disaccharides and their overall proportions
`CleavedSequence`	Represents a cleaved BKHS sequence
`CoordDescNNQP`	Non-negative quadratic programming via coordinate descent
`CSpec`	Heparinase cleavage yield/specificity
`GradientWidth`	Estimates bounds on disaccharide proportions along BKHS via linear programming
`HCModel`	Model H&C
`HCModelLIC`	Linear inequality constraints on a H&C model
`HCModelSA`	Optimization of H&C model parameters via simulated annealing
`HIModel`	Model H&I
`HomogeneityFeasibility`	Feasibility problem for homogeneity of disaccharide composition along BKHS chains
`LinEqCons`	Convenience wrapper for linear constraints
`MatrixOp`	Methods for vector and matrix operations
`MaxEntModel`	Maximum entropy model when all BKHS chains have same length
`MaxEntModelW`	Maximum entropy model for a mixture of BKHS chain lengths
`MaxEntOptim`	Estimation of a MaxEnt model via geometric programming
`MixSpecies`	Model of a mixture of BKHS chain lengths, including enumeration of all sequences
`NIModel`	Model N&I
`NIModelLIC`	Linear inequality constraints on model N&I
`NIModelSA`	Optimization of N&I model parameters via simulated annealing
`ProfileFeasibility`	Estimates infeasibility of the full constraint set (overall disaccharide composition and *heparinase* digests) for different values n of BKHS chain length
`ProjOnPolyHSet`	Projection onto a polyhedral set via coordinate descent
`SandVal`	Convenience class to sort pairs of variables
`SFormCons`	Represents a linear constraint $\mathbf{Ax} = \mathbf{b}$ with $\mathbf{b} \geq \mathbf{0}$
`Simplex`	Simplex method for linear programming (phase II)
`SimplexPhaseI`	Simplex method for linear programming (phase I)
`Species`	Enumeration of BKHS sequences for chains of fixed length
`Tableau`	Tableau for the simplex method
`Utils`	General utilities

Table S4 provide a brief description for each one of the 29 classes and the code is listed on the next pages.

4.3 Code

```
import java.util.Random;
/**
 * creates an affine constraint in standard form (Ax = b, with rank(A) < dim(b)
 * and b >= 0)and with artificial variables
 * (for phase I of the simplex)
 */
public class AVSFormCons {
  /**
   * affine constraint in standard form, before adding artificial variables
   */
  SFormCons sfc = null;
  /**
   * number of variables, after adding artificial ones
   */
  int n = 0;
  /**
   * number of equality constraints
   */
  int m = 0;
  /**
   * matrix for the constraints, after adding artificial variables
   */
  double[][] A = null;
  /**
   * vector of constraints, in standard form
   */
  double[] b = null;
  /**
   * creates an affine constraint in standard canonical form (with added artificial
   * variables) for phase I of the simplex: [I A] x = b, with b >= 0
   * @param mA constraint matrix
   * @param mb constraint values
   */
  public AVSFormCons(double[][] mA, double[] mb){
    sfc = new SFormCons(mA, mb);
    m = sfc.m;
    n = sfc.m+sfc.n;
    b = new double[m];
    A = new double[m][n];
    for (int i = 0; i < m; i++){
      b[i] = sfc.b[i];
      A[i][i] = 1.;
    }
    for (int i = 0; i < m; i++){
      for (int j = 0; j < sfc.n; j++){
        A[i][j+m] = sfc.A[i][j];
      }
    }
  }
  /**
   * prints out the constraint
   */
```

```
  void print(){
    for (int i = 0; i < m; i++){
      for (int j = 0; j < n; j++){
        System.out.print(A[i][j]+"\t");
      }
      System.out.println(b[i]);
    }
  }
  /**
   * for testing
   * @param args
   */
  public static void main(String[] args){
    int M = 10;
    int N = 15;
    Random rand = new Random();
    double[][] mA = new double[M][N];
    double[] mb = new double[M];
    double[] x = new double[N];
    for (int j = 0; j < N; j++)
      x[j] = rand.nextDouble();
    for (int i = 0; i < M; i++){
      mb[i] = 0.;
      for (int j = 0; j < N; j++){
        mA[i][j] = (0.5-rand.nextDouble());
        mb[i] += (mA[i][j]*x[j]);
      }
    }
    new AVSFormCons(mA, mb);
  }
}
```

```
import java.io.BufferedReader;
import java.io.FileReader;
import java.util.Hashtable;
import java.util.StringTokenizer;
import java.util.Vector;

/**
 * Set of building blocks (e.g. S and U), including names and relative abundances
 */
public class BBSet {
  /**
   * number of building blocks
   */
  int m = 0;
  /**
   * building block labels, lower case
   */
  String[] name = null;
  /**
   * building block relative abundances, they must sum to 1
   */
  double[] rho = null;
  /**
   * maps name (lower case) to index in this.name
   */
  Hashtable<String,Integer> name2i = null;
  /**
   * creates a set of building blocks (names and relative abundances) from a file
   * @param file ascii tab-delimited with one header row: name \t abundance
   */
  public BBSet(String file){
    Vector<String> v = new Vector<String>();
    try{
      FileReader fr = new FileReader(file);
      BufferedReader br = new BufferedReader(fr);
      String line = br.readLine();
      while((line = br.readLine()) != null){
        StringTokenizer st = new StringTokenizer(line, "\t");
        if (st.countTokens() == 2){
          v.add(line);
        }
      }
      br.close();
      fr.close();
    }
    catch(Exception ex){
      ex.printStackTrace();
    }
    m = v.size();
    name = new String[m];
    rho = new double[m];
    name2i = new Hashtable<String,Integer>();
    for (int i = 0; i < m; i++){
```

```
      String s = v.elementAt(i);
      StringTokenizer st = new StringTokenizer(s, "\t");
      name[i] = new String(st.nextToken().trim().toLowerCase());
      name2i.put(name[i], new Integer(i));
      Double D = new Double(st.nextToken().trim());
      rho[i] = D.doubleValue();
    }
    double sum = 0.;
    for (int i = 0; i < m; i++){
      sum += rho[i];
    }
    for (int i = 0; i < m; i++){
      rho[i] /= sum;
    }
  }
  /**
   * for testing
   * @param args
   */
  public static void main(String[] args){
    String file = "input\\US.ab.txt";
    BBSet bs = new BBSet(file);
    for (int i = 0; i < bs.m; i++){
      System.out.println(bs.name[i]+"\t"+bs.rho[i]);
    }
  }
}
```

```
/**
 * Represents a chain that was cut (cleaved). This includes chain sequence,
 * the cut positions and methods to return the fragments. This class is
 * utilized for numerical check (cleavage simulations) of derived equations
 * for distributions of fragment lengths
 */
public class CleavedSequence {
  /**
   * Sequence of the original chain.
   */
  int[] oriseq = null;
  /**
   * Positions of the cuts. By convention, cuts happen on the left side
   * and for a chain of length n there is always a cut at 0 and at n
   */
  int[] cutPos = null;
  /**
   * Creates a new cut sequence of a chain.
   * @param seq chain sequence
   * @param cuts Positions of the cut
   */
  public CleavedSequence(int[] seq, int[] cuts){
    oriseq = seq;
    cutPos = cuts;
  }
  /**
   * Returns the fragments as an array of sequences,
   * each sequence being represented by int[].
   * This method returns fragments in the same order as getOrigins.
   * @return fragments as an int[][]
   */
  int[][] getFragments(){
    int[][] res = new int[cutPos.length-1][];
    for (int i = 0; i < cutPos.length-1; i++){
      int l = cutPos[i+1]-cutPos[i];
      res[i] = new int[l];
      int pos = -1;
      for (int j = cutPos[i]; j < cutPos[i+1]; j++){
        pos++;
        res[i][pos] = oriseq[j];
      }
    }
    return res;
  }
  /**
   * for testing
   * @param args
   */
  public static void main(String[] args){
    int[] seq = new int[10];
    for (int i = 0; i < 10; i++)
      seq[i] = i+1;
    int[] cuts = new int[10];
```

```
    cuts[0] = 0;
    cuts[1] = 1;
    cuts[2] = 2;
    cuts[3] = 3;
    cuts[4] = 4;
    cuts[5] = 5;
    cuts[6] = 6;
    cuts[7] = 7;
    cuts[8] = 8;
    cuts[9] = 10;
    CleavedSequence cs = new CleavedSequence(seq, cuts);
    int[][] test = cs.getFragments();
    for (int i = 0; i < cs.oriseq.length; i++)
      System.out.print(cs.oriseq[i]);
    System.out.println("");
    for (int i = 0; i < test.length; i++){
      for (int j = 0; j < test[i].length; j++)
        System.out.print(test[i][j]);
      System.out.println("");
    }
  }
}
```

```
/**
 * solution to nonnegative quadratic programming:
 * minimize (1/2)x^TQx + c^Tx subject to x >= 0
 * via coordinate descent. Note that Q must be symmetric
 * and positive definite
 */
public class CoordDescNNQP {
  /**
   * dimension of x (Q is n*n)
   */
  int n = 0;
  /**
   * quadratic coefficients in the cost (1/2)x^TQx + c^Tx
   */
  double[][] Q = null;
  /**
   * linear coefficients in the cost (1/2)x^TQx + c^Tx
   */
  double[] c = null;
  /**
   * point being iterated
   */
  double[] x = null;
  /**
   * keeps track of the number of iterations (1 iter == change of
   * all components x_1, x_2, ..., x_n)
   */
  public int iter = 0;
  /**
   * maximum number of iterations (1e6); if it goes above,
   * matrix Q is likely ill-conditioned
   */
  int maxIter = 1000000;
  /**
   * final value of the objective function (1/2)x^TQx + c^Tx
   */
  double finalCost = 0.;
  /**
   * solution
   */
  public double[] optimum = null;
  /**
   * used to stop iterations
   */
  double eps = 1e-10;
  /**
   * certificate
   */
  boolean success = false;
  /**
   * Solves: minimize (1/2)x^TQx + c^Tx subject to x >= 0.
   * Solution x is in this.optimum
   * @param Q coefficients of the quadratic terms in the cost function
```

```
 * @param c coefficients of the linear terms in the cost function
 * @param y starting point (any random point in the positive orthant)
 */
public CoordDescNNQP(double[][] Q, double[] c, double[] y){
  // initializations
  n = Q.length;
  this.Q = Q;
  this.c = c;
  x = new double[n];
  for (int j = 0; j < n; j++){
    x[j] = y[j];
  }
  double alpha = 1.;
  iter = 0;
  double cost = getCost(x);
  boolean b = true;
  // coordinate descent method
  while(b){
    iter++;
    alpha = 0.;
    for (int j = 0; j < n; j++){
      double t = c[j];
      for (int k = 0; k < n; k++){
        t += (Q[j][k]*x[k]);
      }
      t /= Q[j][j];
      t *= -1.;
      t += x[j];
      if (t >= 0.){
        alpha += ((x[j]-t)*(x[j]-t));
        x[j] = t;
      }
      else{
        alpha += (x[j]*x[j]);
        x[j] = 0.;
      }
    }
    alpha = Math.sqrt(alpha);
    alpha /= (double) x.length;
    cost = getCost(x);
    if (alpha < eps){
      b = false;
      success = true;
    }
    if (b){
      if (iter > maxIter){
        success = false;
        b = false;
      }
    }
  }
  // solution and final cost
  optimum = new double[n];
  for (int j = 0; j < n; j++){
    optimum[j] = x[j];
```

```
    }
    finalCost = cost;
  }
  /**
   * computes (1/2)z^TQz + c^Tz
   * @param z
   * @return  (1/2)z^TQz + c^Tz
   */
  double getCost(double[] z){
    double res = 0.;
    res = MatrixOp.geInnerProd(MatrixOp.multMatVec(Q, z), z);
    res /= 2.;
    res += MatrixOp.geInnerProd(c, z);
    return res;
  }
  /**
   * returns z^Tz
   * @param z vector
   * @return z^Tz
   */
  double getNorm2(double[] z){
    double res = 0.;
    for (int j = 0; j < z.length; j++){
      res += (z[j]*z[j]);
    }
    return res;
  }
}
```

```java
import java.io.BufferedReader;
import java.io.FileReader;
import java.util.StringTokenizer;
import java.util.Vector;
/**
 * cleavage specificities, based on a BBSet object (set of building blocks)
 * and a file giving specificity for each building block name
 */
public class CSpec {
  /**
   * number of building blocks
   */
  int m = 0;
  /**
   * cleavage specificities
   */
  double[] c = null;
  /**
   * Creates cleavage specificities, based on a BBSet object (set of building blocks) and
   * a file giving specificity for each building block name
   * @param file ascii tab-delimited file with one header row: name \t specificity
   * @param bbset set of building blocks
   */
  public CSpec(String file, BBSet bbset){
    Vector<String> v = new Vector<String>();
    try{
      FileReader fr = new FileReader(file);
      BufferedReader br = new BufferedReader(fr);
      String line = br.readLine();
      while((line = br.readLine()) != null){
        StringTokenizer st = new StringTokenizer(line, "\t");
        if (st.countTokens() == 2){
          v.add(line);
        }
      }
      br.close();
      fr.close();
    }
    catch(Exception ex){
      ex.printStackTrace();
    }
    m = v.size();
    c = new double[m];
    for (int i = 0; i < m; i++){
      String s = v.elementAt(i);
      StringTokenizer st = new StringTokenizer(s, "\t");
      String name = st.nextToken().trim().toLowerCase();
      Integer I = bbset.name2i.get(name);
      Double D = new Double(st.nextToken().trim());
      c[I.intValue()] = D.doubleValue();
    }
  }
  /**
```

```
   * for testing
   * @param args
   */
  public static void main(String[] args){
    String inDir = "input\\";
    String file = inDir+"US.ab.txt";
    BBSet bbs = new BBSet(file);
    file = inDir+"US.hepI.txt";
    CSpec cs = new CSpec(file, bbs);
    for (int i = 0; i < cs.m; i++){
      System.out.println(cs.c[i]);
    }
    file = inDir+"US.hepIII.txt";
    cs = new CSpec(file, bbs);
    for (int i = 0; i < cs.m; i++){
      System.out.println(cs.c[i]);
    }
  }
}
```

```
import java.util.Vector;
/**
 * utilizes linear programming to estimate bounds of S/U proportions at each position
 * in chains; two different constructors: one for all BKHS chains having same length
 * and one for a mixture of chain lengths
 */
public class GradientWidth {
  /**
   * enumeration of all possible sequences, one chain length only
   */
  Species sp = null;
  /**
   * matrix in constraint Ap = b
   */
  double[][] A = null;
  /**
   * vector in constraint Ap = b
   */
  double[] b = null;
  /**
   * convenience wrapper for the constraints
   */
  LinEqCons lec = null;
  /**
   * phase I of the simplex
   */
  SimplexPhaseI sp1 = null;
  /**
   * output of phase I
   */
  double infeasibility = 0.;
  /**
   * disaccharide labels
   */
  String[] lab = null;
  /**
   * enumeration of all sequences, mixture of chain lengths
   */
  MixSpecies msp = null;
  /**
   * utilizes linear programming to estimate bounds of S/U proportions at each position;
   * constructor for a mixture of BKHS chain length (average mu and spread sigma),
   * Note: the second constructor requires large memory (e.g. -Xmx8000M)
   * @param lb disaccharide labels
   * @param lmi smallest chain length
   * @param lmx largest chain length
   * @param sig spread of chain length distribution
   * @param mu average chain length
   * @param inDir input directory (ends with "\\")
   */
  public GradientWidth(String[] lb, int lmi, int lmx, double sig, double mu, String inDir){
    this.lab = lb;
    BBSet bbs = new BBSet(inDir+"US.ab.txt");
```

```
    msp = new MixSpecies(lmi, lmx, sig, mu);
    String[] csFile = new String[2];
    String[] fragFile = new String[2];
    csFile[0] = new String(inDir+"US.hepI.txt");
    csFile[1] = new String(inDir+"US.hepIII.txt");
    fragFile[0] = new String(inDir+"hepI.f.txt");
    fragFile[1] = new String(inDir+"hepIII.f.txt");
    lec = msp.getCompleteLEC(bbs, csFile, fragFile);
    A = lec.A;
    b = lec.b;
    sp1 = new SimplexPhaseI(A, b);
    infeasibility = sp1.finalCost;
    System.err.println(infeasibility);
  }
  /**
   * utilizes linear programming to estimate bounds of S/U proportions at each position;
   * constructor for the case of all BKHS chains having same length n
   * @param m number of disaccharides
   * @param n BKHS chain length
   * @param lab disaccharide labels
   * @param inDir input directory (ends with "\\")
   */
  public GradientWidth(int m, int n, String[] lab, String inDir){
    this.lab = lab;
    sp = new Species(m, n);
    BBSet bbs = new BBSet(inDir+"US.ab.txt");
    String[] csFile = new String[2];
    String[] fragFile = new String[2];
    csFile[0] = new String(inDir+"US.hepI.txt");
    csFile[1] = new String(inDir+"US.hepIII.txt");
    fragFile[0] = new String(inDir+"hepI.f.txt");
    fragFile[1] = new String(inDir+"hepIII.f.txt");
    lec = sp.getCompleteLEC(bbs, csFile, fragFile);
    A = lec.A;
    b = lec.b;
    sp1 = new SimplexPhaseI(A, b);
    infeasibility = sp1.finalCost;
  }
  /**
   * linear cost coefficients for presence of bb at position pos,
   * when chains are aligned by the NRE; mixture of chain lengths
   * @param pos position in chains (from NRE)
   * @param bb disaccharide
   * @return linear cost coefficients for presence of bb at position pos,
   * when chains are aligned by the NRE
   */
  double[] getCostCoeffNRE(int pos, int bb){

    double[] res = new double[msp.N];
    for (int s = 0; s < msp.N; s++){
      if (pos < msp.seq[s].length){
        if (msp.seq[s][pos] == bb){
          res[s] = 1.;
        }
      }
```

```
  }
  return res;
}
/**
 * linear cost coefficients for presence of bb at position pos,
 * when chains are aligned by the RE; mixture of chain lengths
 * @param pos position from RE
 * @param bb disaccharide
 * @return linear cost coefficients for presence of bb at position pos,
 * when chains are aligned by the RE
 */
double[] getCostCoeffRE(int pos, int bb){
  double[] res = new double[msp.N];
  for (int s = 0; s < msp.N; s++){
    if (pos < msp.seq[s].length){
      if (msp.seq[s][msp.seq[s].length-1-pos] == bb){
        res[s] = 1.;
      }
    }
  }
  return res;
}
/**
 * linear cost coefficients for bb at position pos
 * when all BKHS have same length
 * @param pos position from NRE
 * @param bb disaccharide
 * @return linear cost coefficients for bb at position pos
 * when all BKHS have same length
 */
double[] getCostCoeff(int pos, int bb){
  double[] res = new double[sp.N];
  for (int s = 0; s < sp.N; s++){
    if (sp.seq[s][pos] == bb){
      res[s] = 1.;
    }
  }
  return res;
}
/**
 * lower bound for proportion of bb at position pos
 * when chains are aligned by NRE; mixture of chain lengths
 * @param pos position from the NRE
 * @param bb disaccharide
 * @return lower bound for proportion of bb at position pos
 * when chains are aligned by NRE; mixture of chain lengths
 */
double getLowerNRE(int pos, int bb){
  double[] c = getCostCoeffNRE(pos, bb);
  Simplex sp = new Simplex(sp1, c);
  double res = sp.finalCost;
  if (res > 1.){
    res = 1.;
  }
  if (res < 0.){
```

```
    res = 0.;
  }
  return res;
}
/**
 * lower bound for proportion of bb at position pos
 * when chains are aligned by RE; mixture of chain lengths
 * @param pos position from RE
 * @param bb disaccharide
 * @return lower bound for proportion of bb at position pos
 * when chains are aligned by RE; mixture of chain lengths
 */
double getLowerRE(int pos, int bb){
  double[] c = getCostCoeffRE(pos, bb);
  Simplex sp = new Simplex(sp1, c);
  double res = sp.finalCost;
  if (res > 1.){
    res = 1.;
  }
  if (res < 0.){
    res = 0.;
  }
  return res;
}
/**
 * upper bound for proportion of bb at position pos
 * when chains are aligned by NRE; mixture of chain lengths
 * @param pos position from NRE
 * @param bb disaccharide
 * @return upper bound for proportion of bb at position pos
 * when chains are aligned by NRE; mixture of chain lengths
 */
double getUpperNRE(int pos, int bb){
  double[] c = getCostCoeffNRE(pos, bb);
  for (int i = 0; i < c.length; i++){
    c[i] *= -1.;
  }
  Simplex sp = new Simplex(sp1, c);
  double res = -sp.finalCost;
  if (res > 1.){
    res = 1.;
  }
  if (res < 0.){
    res = 0.;
  }
  return res;
}
/**
 * upper bound for proportion of bb at position pos
 * when chains are aligned by RE; mixture of chain lengths
 * @param pos position from RE
 * @param bb disaccharide
 * @return upper bound for proportion of bb at position pos
 * when chains are aligned by RE; mixture of chain lengths
 */
```

```
double getUpperRE(int pos, int bb){
  double[] c = getCostCoeffRE(pos, bb);
  for (int i = 0; i < c.length; i++){
    c[i] *= -1.;
  }
  Simplex sp = new Simplex(sp1, c);
  double res = -sp.finalCost;
  if (res > 1.){
    res = 1.;
  }
  if (res < 0.){
    res = 0.;
  }
  return res;
}
/**
 * lower bound for proportion of bb at position pos
 * when all BKHS chains have same length
 * @param pos position from the NRE
 * @param bb disaccharide
 * @return lower bound for proportion of bb at position pos
 * when all BKHS chains have same length
 */
double getLower(int pos, int bb){
  double[] c = getCostCoeff(pos, bb);
  Simplex sp = new Simplex(sp1, c);
  double res = sp.finalCost;
  if (res > 1.){
    res = 1.;
  }
  if (res < 0.){
    res = 0.;
  }
  return res;
}
/**
 * upper bound for proportion of bb at position pos
 * when all BKHS chains have same length
 * @param pos position from NRE
 * @param bb disaccharide
 * @return upper bound for proportion of bb at position pos
 * when all BKHS chains have same length
 */
double getUpper(int pos, int bb){
  double[] c = getCostCoeff(pos, bb);
  for (int i = 0; i < c.length; i++){
    c[i] *= -1.;
  }
  Simplex sp = new Simplex(sp1, c);
  double res = -sp.finalCost;
  if (res > 1.){
    res = 1.;
  }
  if (res < 0.){
    res = 0.;
```

```
    }
    return res;
  }
  /**
   * saves bounds for S/U proportions at each position
   * when all BKHS chains have same length
   * @param file output file
   */
  void saveBounds(String file){
    Vector<String> v = new Vector<String>();
    String s = "pos";
    for (int i = 0; i < sp.m; i++){
      s += "\tlower"+lab[i]+"\tupper"+lab[i];
    }
    v.add(s);
    for (int pos = 0; pos < sp.n; pos++){
      //System.err.println("n = "+sp.n+", pos = "+(pos+1));
      Integer I = new Integer(pos+1);
      s = I.toString();
      for (int j = 0; j < sp.m; j++){
        Double D = new Double(getLower(pos,j));
        s += "\t"+D.doubleValue();
        D = new Double(getUpper(pos,j));
        s += "\t"+D.doubleValue();
      }
      v.add(s);
      System.err.println(s);
    }
    Utils.saveFile(v, file);
  }
  /**
   * saves bounds of S/U proportions at each position from the NRE
   * for a mixture of BKHS chain lengths
   * @param file output file
   */
  void saveBoundsMixSpNRE(String file){
    Vector<String> v = new Vector<String>();
    String s = "posNRE";
    for (int i = 0; i < msp.m; i++){
      s += "\tlower"+lab[i]+"\tupper"+lab[i];
    }
    v.add(s);
    for (int pos = 0; pos < msp.lmax; pos++){
      Integer I = new Integer(pos+1);
      s = I.toString();
      for (int j = 0; j < msp.m; j++){
        Double D = new Double(getLowerNRE(pos,j)/msp.lengthAtLeast[pos]);
        s += "\t"+D.doubleValue();
        D = new Double(getUpperNRE(pos,j)/msp.lengthAtLeast[pos]);
        s += "\t"+D.doubleValue();
      }
      v.add(s);
      System.err.println(s);
    }
    Utils.saveFile(v, file);
```

```
}
/**
 * saves bounds of S/U proportions at each position from the RE
 * for a mixture of BKHS chain lengths
 * @param file output file
 */
void saveBoundsMixSpRE(String file){
  Vector<String> v = new Vector<String>();
  String s = "posRE";
  for (int i = 0; i < msp.m; i++){
    s += "\tlower"+lab[i]+"\tupper"+lab[i];
  }
  v.add(s);
  for (int pos = 0; pos < msp.lmax; pos++){
    Integer I = new Integer(pos+1);
    s = I.toString();
    for (int j = 0; j < msp.m; j++){
      Double D = new Double(getLowerRE(pos,j)/msp.lengthAtLeast[pos]);
      s += "\t"+D.doubleValue();
      D = new Double(getUpperRE(pos,j)/msp.lengthAtLeast[pos]);
      s += "\t"+D.doubleValue();
    }
    v.add(s);
    System.err.println(s);
  }
  Utils.saveFile(v, file);
}
/**
 * makes and saves S/U bounds at each position
 * when all BKHS chains have same length
 * @param inDir input directory (ends with "\\")
 * @param outDir output directory (ends with "\\")
 */
public static void bounds(String inDir, String outDir){
  String[] lab = new String[2];
  lab[0] = new String("U");
  lab[1] = new String("S");
  GradientWidth gw = new GradientWidth(2,16,lab, inDir);
  gw.saveBounds(outDir+"GradientWidth.res");
}
/**
 * for supplementary material; bounds for different values of chain length n
 * @param inDir input directory (ends with "\\")
 * @param outDir output directory (ends with "\\")
 */
public static void boundsAndN(String inDir, String outDir){
  String[] lab = new String[2];
  lab[0] = new String("U");
  lab[1] = new String("S");
  for (int n = 13; n <= 18; n++){
    Integer I = new Integer(n);
    GradientWidth gw = new GradientWidth(2,n,lab, inDir);
    gw.saveBounds(outDir+"GradientWidth.N"+I.toString()+".res");
  }
}
```

```
  /**
   * bounds of S/U proportions at each position for mixture of BKHS chain lengths
   */
  public static void defaultMixSpec(String inDir, String outDir){
    String[] lab = new String[2];
    lab[0] = new String("U");
    lab[1] = new String("S");
    GradientWidth gw = new GradientWidth(lab, 10, 20, 3.5, 16., inDir);
    gw.saveBoundsMixSpNRE(outDir+"BoundsMixSpNRE.res");
    gw.saveBoundsMixSpRE(outDir+"BoundsMixSpRE.res");
  }
  /**
   *
   * @param args
   */
  public static void main(String[] args){

    String inDir = "input\\";
    String outDir = "output\\";
    bounds(inDir, outDir);
    boundsAndN(inDir, outDir);
    defaultMixSpec(inDir, outDir);
  }
}
```

```
import java.util.Random;
import java.util.Vector;
/**
 * Homogeneous Markov model of species (chain sequence) abundances (model H&C);
 * field this.g contains the expected fragment length distribution after digestion
 * by one heparinase
 */
public class HCModel {
  /**
   * number of building blocks
   */
  int m = 0;
  /**
   * chain length
   */
  int n = 0;
  /**
   * matrix of transition probabilities
   */
  double[][] P = null;
  /**
   * cumulative P: pF[i][j] = sum_{k=0}^j P[i][k], utilized for random drawing
   */
  double[][] pF = null;
  /**
   * powers of P
   */
  double[][][] pi = null;
  /**
   * overall cleavage probability
   */
  double c = 0.;
  /**
   * set of building blocks
   */
  BBSet bbs = null;
  /**
   * cleavage specificities/yields
   */
  CSpec cs = null;
  /**
   * fragment length distribution
   */
  double[] g = null;
  /**
   * fragment length distribution with cumulative abundance for length >= lm
   */
  double[] h = null;
  /**
   * maximum fragment length in experimental data
   */
  int lm = 0;
  /**
```

```
 * cumulative version of overall building-block composition in this.bbs.rho
 */
double[] rhoF = null;
/**
 * Homogeneous Markov model of species (chain sequence) abundances (model H&C);
 * field this.g contains the expected fragment length distribution after digestion
 * by one heparinase
 * @param n BKHS chain length
 * @param bbs disaccharides and their overall proportions
 * @param cs cleavage specificities/yields for one heparinase
 * @param tp initial matrix of transition probabilities (random or already optimized)
 * which is then projected to satisfy constraints: output in this.P
 * @param lm maximum fragment length
 */
public HCModel(int n, BBSet bbs, CSpec cs, double[][] tp, int lm){
  this.n = n;
  this.bbs = bbs;
  m = bbs.m;
  this.cs = cs;
  this.lm = lm;
  P = new double[m][m];
  pF = new double[m][m];
  for (int i = 0; i < m; i++){
    double s = 0.;
    for (int j = 0; j < m; j++){
      s += tp[i][j];
    }
    for (int j = 0; j < m; j++){ // projection is not always perfect due to numerical accurarcy
      P[i][j] = tp[i][j]/s;
    }
    pF[i][0] = P[i][0];
    for (int j = 1; j < m; j++){
      pF[i][j] = pF[i][j-1]+P[i][j];
    }
  }
  rhoF = new double[m];
  rhoF[0] = bbs.rho[0];
  for (int i = 1; i < m; i++){
    rhoF[i] = rhoF[i-1]+bbs.rho[i];
  }
  makeC();
  makeG();
  makeH();
}
/**
 * computes expected distribution of fragment length (this.h) cumulative for length >= lm
 */
void makeH(){
  h = new double[n];
  for (int i = 0; i < lm; i++){
    h[i] = g[i];
  }
  for (int i = lm; i < n-1; i++){
    h[lm-1] += g[i];
  }
```

```
}
/**
 * computes expected distribution of fragment length (this.g)
 */
void makeG(){
  pi = new double[n][m][m];
  for (int i = 0; i < m; i++){
    for (int j = 0; j < m; j++){
      pi[0][i][j] = P[i][j]*(1.-cs.c[j]);
    }
  }
  for (int i = 1; i < n; i++){
    pi[i] = MatrixOp.multMat(pi[i-1], pi[0]);
  }
  g = new double[n];
  // ll = 1
  g[0] = 0.;
  for (int i = 0; i < m; i++){
    for (int j = 0; j < m; j++){
      g[0] += bbs.rho[i]*cs.c[i]*cs.c[j]*P[i][j];
    }
  }
  g[0] /= c;
  g[0] *= (double) (n-2);
  g[0] += 1.;
  g[0] /= (double) (n-1);
  // 2 <= ll <= n-1
  for (int l = 1; l < n-1; l++){
    int ll = l+1;
    g[l] = 0.;
    for (int u = 0; u < m; u++){
      double tu = 0.;
      for (int v = 0; v < m; v++){
        double tv = 0.;
        for (int w = 0; w < m; w++){
          tv += P[v][w]*cs.c[w];
        }
        tv *= (double) (n-ll-1);
        tv += 1.;
        tv *= pi[l-1][u][v];
        tu += tv;
      }
      g[l] += bbs.rho[u]*cs.c[u]*tu;
    }
    g[l] /= (double) (n-1);
    g[l] /= c;
  }
}
/**
 * computes parameter c of H&C
 */
void makeC(){
  c = 0.;
  for (int i = 0; i < m; i++){
    c += bbs.rho[i]*cs.c[i];
```

```
  }
}
/**
 * generates a random BKHS sequence based on this.P
 * @param rand
 * @return a random BKHS sequence based on this.P
 */
int[] getSequence(Random rand){
  int[] res = new int[n];
  res[0] = Utils.getRandIndexInF(rhoF, rand);
  for (int i = 1; i < n; i++){
    res[i] = Utils.getRandIndexInF(pF[res[i-1]], rand);
  }
  return res;
}
/**
 * generates a random set of cleavage positions in BKHS sequence seq based
 * on heparinase cleavage specificities
 * @param seq BKHS sequence
 * @param rand
 * @return a random set of cleavage positions in BKHS sequence seq based
 * on heparinase cleavage specificities
 */
int[] getCuts(int[] seq, Random rand){
  Vector<Integer> cuts = new Vector<Integer>();
  for (int i = 1; i < n; i++){
    double d = rand.nextDouble();
    if (d < cs.c[seq[i]]){
      cuts.add(new Integer(i));
    }
  }
  cuts.add(new Integer(n));
  int[] res = new int[cuts.size()];
  for (int i = 0; i < cuts.size(); i++){
    Integer I = cuts.elementAt(i);
    res[i] = I.intValue();
  }
  return res;
}
/**
 * generates a random CutSequence (generates a BKHS chain and randomly cleaves
 * it based on cleavage specificities); see method getFragments() of class
 * CutSequence to access resulting fragments
 * @param rand
 * @return a random CutSequence (generates a BKHS chain and randomly cleaves
 * it based on cleavage specificities)
 */
CleavedSequence getCutSequence(Random rand){
  int[] seq = null;
  int[] cuts = new int[1];
  while(cuts.length < 2){
    seq = getSequence(rand);
    cuts = getCuts(seq, rand);
  }
  return new CleavedSequence(seq, cuts);
```

```
}
/**
 * numerical check for this.g
 * @param rand
 */
void checkGL(Random rand){
  int nsim = 10000000;
  int totfrag = 0;
  double[] f = new double[n];
  for (int sim = 0; sim < nsim; sim++){
    CleavedSequence cseq = getCutSequence(rand);
    int[][] frag = cseq.getFragments();
    totfrag += frag.length;
    for (int i = 0; i < frag.length; i++){
      f[frag[i].length-1] += 1.;
    }
  }
  for (int i = 0; i < n; i++){
    System.out.println((i+1)+"\t"+g[i]+"\t"+(f[i]/(double) totfrag));
  }
}
/**
 * returns a vector version of matrix P
 * @param P
 * @return a vector version of matrix P
 */
public static double[] toVector(double[][] P){
  double[] res = new double[P.length*P.length];
  int k = -1;
  for (int i = 0; i < P.length; i++){
    for (int j = 0; j < P.length; j++){
      k++;
      res[k] = P[i][j];
    }
  }
  return res;
}
/**
 * returns a matrix version of vector p
 * @param m number of rows/columns
 * @param p vector
 * @return a m by m matrix version of vector p
 */
public static double[][] toMatrix(int m, double[] p){
  double[][] res = new double[m][m];
  int k =-1;
  for (int i = 0; i < m; i++){
    for (int j = 0; j < m; j++){
      k++;
      res[i][j] = p[k];
    }
  }
  return res;
}
/**
```

```
   * for testing
   * @param args
   */
  public static void main(String[] args){
    String inDir = "input\\";
    String file = inDir+"US.ab.txt";
    BBSet bbs = new BBSet(file);
    file = inDir+"US.hepI.txt";
    CSpec cs = new CSpec(file, bbs);
    int m = cs.m;
    int n = 16;
    double[][] P = new double[m][m];
    Random rand = new Random();
    for (int i = 0; i < m; i++){
      for (int j = 0; j < m; j++){
        P[i][j] = rand.nextDouble();
      }
    }
    HCModelLIC mlic = new HCModelLIC(bbs);
    ProjOnPolyHSet proj = new ProjOnPolyHSet(toVector(P), mlic.A, mlic.b, rand);
    P = toMatrix(m, proj.optimum);
    int lm = 11;
    HCModel mm = new HCModel(n, bbs, cs, P, lm);
    mm.checkGL(rand);
  }
}
```

```
import java.util.Random;
/**
 * linear inequality constraints on a H&C model (homogeneous Markov model):
 * nonnegativity, balance equation and stochastic matrix
 */
public class HCModelLIC {
  /**
   * matrix in Ax <= b
   */
  double[][] A = null;
  /**
   * vector in Ax <= b
   */
  double[] b = null;
  /**
   * number of building blocks
   */
  int m = 0;
  /**
   * linear inequality constraints on a H&C model (homogeneous Markov model):
   * nonnegativity, balance equation and stochastic matrix
   * @param n chain length
   * @param bbs set of building blocks
   */
  public HCModelLIC(BBSet bbs){
    m = bbs.m;
    A = new double[m*(m+4)][m*m];
    b = new double[m*(m+4)];
    // nonnegativity
    for (int rr = 1; rr <= m*m; rr++){
      int r = rr-1;
      A[r][r] = -1.;
      b[r] = 0.;
    }
    // balance equations: <= rho
    for (int rr = m*m+1; rr <= m*m+m; rr++){
      int r = rr-1;
      b[r] = bbs.rho[r-m*m];
      for (int kk = 1; kk <= m*m; kk++){
        int k = kk-1;
        if (getJJ(kk) == rr-m*m){
          A[r][k] = bbs.rho[getII(kk)-1];
        }
      }
    }
    // balance equations: <= -rho
    for (int rr = m*m+m+1; rr <= m*m+2*m; rr++){
      int r = rr-1;
      b[r] = -bbs.rho[r-m*m-m];
      for (int kk = 1; kk <= m*m; kk++){
        int k = kk-1;
        if (getJJ(kk) == rr-m*m-m){
          A[r][k] = -bbs.rho[getII(kk)-1];
```

```
      }
    }
  }
  // stochastic matrix: <= 1
  for (int rr = m*m+2*m+1; rr <= m*m+3*m; rr++){
    int r = rr-1;
    b[r] = 1.;
    for (int kk = 1; kk <= m*m; kk++){
      int k = kk-1;
      if (getII(kk) == rr-m*m-2*m){
        A[r][k] = 1.;
      }
    }
  }
  // stochastic matrix: <= -1
  for (int rr = m*m+3*m+1; rr <= m*m+4*m; rr++){
    int r = rr-1;
    b[r] = -1.;
    for (int kk = 1; kk <= m*m; kk++){
      int k = kk-1;
      if (getII(kk) == rr-m*m-3*m){
        A[r][k] = -1.;
      }
    }
  }
}
/**
 * index in the vector representation of matrix P for entry Pij
 * @param ii row index (1 to m)
 * @param jj column index (1 to m)
 * @return index kk (1 to m^2) in the vector representation of matrix
 * P for entry Pij
 */
int getKK(int ii, int jj){
  int res = jj+(ii-1)*m;
  return res;
}
/**
 * row index (1 to m) in matrix P for component p_kk of its vector representation
 * @param kk index of Pij in its vector representation p_kk (1 to m^2)
 * @return row index (1 to m) in matrix P for component p_kk of its vector
 * representation
 */
int getII(int kk){
  int res = 1+(kk-1)/m;
  return res;
}
/**
 * column index (1 to m) in matrix P for component p_kk of its vector representation
 * @param kk index of Pij in its vector representation p_kk (1 to m^2)
 * @return column index (1 to m) in matrix P for component p_kk of its vector
 * representation
 */
int getJJ(int kk){
  int res = kk - (getII(kk)-1)*m;
```

```
    return res;
  }
  /**
   * for testing
   * @param args
   */
  public static void main(String[] args){
    String inDir = "input\\";
    String file = inDir+"US.ab.txt";
    BBSet bbs = new BBSet(file);
    HCModelLIC mlic = new HCModelLIC(bbs);
    Random rand = new Random();
    double[][] P = new double[bbs.m][bbs.m];
    for (int i = 0; i < bbs.m; i++){
      for (int j = 0; j < bbs.m; j++){
        P[i][j] = rand.nextDouble();
      }
    }
    double[] p = HCModel.toVector(P);
    ProjOnPolyHSet proj = new ProjOnPolyHSet(p, mlic.A, mlic.b, rand);
    P = HCModel.toMatrix(bbs.m, proj.optimum);
    MatrixOp.printMat(P);
    System.out.println("stochastic matrix");
    for (int i = 0; i < bbs.m; i++){
      double t = 0.;
      for (int j = 0; j < bbs.m; j++){
        t += P[i][j];
      }
      System.out.println(t);
    }
    System.out.println("balance equations");
    for (int i = 0; i < bbs.m; i++){
      double t = 0.;
      for (int j = 0; j < bbs.m; j++){
        t += bbs.rho[j]*P[j][i];
      }
      System.out.println(t+"\t"+bbs.rho[i]);
    }
  }
}
```

```
import java.io.BufferedReader;
import java.io.BufferedWriter;
import java.io.FileReader;
import java.io.FileWriter;
import java.util.Random;
import java.util.StringTokenizer;
import java.util.Vector;
/**
 * Optimization of a homogeneous Markov model (model H&C) via simulated
 * annealing to fit two heparinase digest constraint sets
 */
public class HCModelSA {
  /**
   * Markov models (e.g. one for hepI and one for hepIII)
   */
  HCModel[] mm = null;
  /**
   * f[i][] is the distribution of fragment length (experimental data)
   * for digest model i
   */
  double[][] f = null;
  /**
   * for perturbations
   */
  Random rand = null;
  /**
   * chain length
   */
  int n = 0;
  /**
   * number of building blocks
   */
  int m = 0;
  /**
   * number of digests
   */
  int nd = 0;
  /**
   * enzyme specificities
   */
  CSpec[] cs = null;
  /**
   * set of building blocks
   */
  BBSet bbs = null;
  /**
   * Markov model parameters
   */
  double[][] P = null;
  /**
   * best encountered model
   */
  double[][] bestP = null;
```

```
/**
 * buffer for this.P
 */
double[][] buffP = null;
/**
 * linear inequality constraints (inequality version of nonnegativity,
 * sum to 1 and balance equations)
 */
HCModelLIC mlic = null;
/**
 * number of perturbations at each temperature
 */
int nPert = 200;
/**
 * annealing schedule (temperature multiplied by alpha when decreased)
 */
double alpha = 0.999;
/**
 * best objective function value
 */
double bestE = 0.;
/**
 * Optimization of a homogeneous Markov model (model H&C) via simulated annealing
 * to fit two heparinase digest constraint sets
 * @param n BKHS chain length
 * @param bbs disaccharides and their overall proportions
 * @param specFile cleavage specificities
 * @param consFile experimental constraints (files with experimentally measured
 * distributions of fragment length)
 * @param rand
 */
public HCModelSA(int n, BBSet bbs, String[] specFile, String[] consFile,
    String[] outFile, String modFile, Random rand){
  this.bbs = bbs;
  m = bbs.m;
  this.n = n;
  this.rand = rand;
  nd = specFile.length;
  cs = new CSpec[nd];
  for (int i = 0; i < nd; i++){
    cs[i] = new CSpec(specFile[i], bbs);
  }
  loadConstraints(consFile);
  initModel();
  double t = initT();
  int nup = 1;
  double E = bestE;
  while(nup > 0){
    t *= alpha;
    System.err.println(t+"\t"+E+"\t"+bestE+"\t"+nup);
    nup = 0;
    for (int i = 0; i < nPert; i++){
      perturb();
      double E2 = getE();
      boolean accept = false;
```

```
      if (E2 < E){
        accept = true;
      }
      else{
        double p = Math.exp(-Math.abs(E2-E)/t);
        if (rand.nextDouble() < p){
          accept = true;
          nup++;
        }
      }
      if (accept){
        E = E2;
        if (E < bestE){
          bestE = E;
          saveP();
        }
      }
      else{
        restore();
      }
    }
  }
  for (int i = 0; i < m; i++){
    for (int j = 0; j < m; j++){
      P[i][j] = bestP[i][j];
    }
  }
  mm = new HCModel[nd];
  for (int i = 0; i < nd; i++){
    mm[i] = new HCModel(n, bbs, cs[i], P, f[i].length);
    saveFit(outFile[i], i);
  }
  saveMM(modFile);
}
/**
 * saves the optimized transition probability matrix
 * @param file output file for matrix of transition probabilities
 */
void saveMM(String file){
  try{
    FileWriter fw = new FileWriter(file);
    BufferedWriter bw = new BufferedWriter(fw);
    bw.write("prev\tnext\tp");
    bw.newLine();
    for (int i = 0; i < m; i++){
      for (int j = 0; j < m; j++){
        Double D = new Double(P[i][j]);
        bw.write(bbs.name[i]+"\t"+bbs.name[j]+"\t"+D.toString());
        bw.newLine();
      }
    }
    bw.close();
    fw.close();
  }
  catch(Exception ex){
```

```
      ex.printStackTrace();
    }
  }
  /**
   * saves experimental digest data and modeled digest data
   * @param file
   * @param i digest number (heparinase number)
   */
  void saveFit(String file, int i){
    try{
      FileWriter fw = new FileWriter(file);
      BufferedWriter bw = new BufferedWriter(fw);
      bw.write("l\tfexp\thmod");
      bw.newLine();
      for (int l = 0; l < mm[i].lm; l++){
        Integer I = new Integer(l+1);
        Double F = new Double(f[i][l]);
        Double H = new Double(mm[i].h[l]);
        bw.write(I.toString()+"\t"+F.toString()+"\t"+H.toString());
        bw.newLine();
      }
      bw.close();
      fw.close();
    }
    catch(Exception ex){
      ex.printStackTrace();
    }
  }
  /**
   * buffers encountered optimal P
   */
  void saveP(){

    bestP = new double[m][m];
    for (int i = 0; i < m; i++){
      for (int j = 0; j < m; j++){
        bestP[i][j] = P[i][j];
      }
    }
  }
  /**
   * estimation of initial temperature (pr(upward jump) = 0.6)
   * @return estimation of initial temperature (pr(upward jump) = 0.6)
   */
  double initT(){
    double res = 0.;
    bestE = getE();
    saveP();
    double dE = 0.;
    for (int i = 0; i < nPert; i++){
      perturb();
      dE += Math.abs(bestE-getE());
      restore();
    }
    dE /= (double) nPert;
```

```
  res = -dE/Math.log(0.6);
  return res;
}
/**
 * perturbation of transition probability matrix P (random perturbation of one
 * entry followed by projection) after buffering the current matrix in case
 * the perturbation is not accepted
 */
void perturb(){
  for (int i = 0; i < m; i++){
    for (int j = 0; j < m; j++){
      buffP[i][j] = P[i][j];
    }
  }
  P[rand.nextInt(m)][rand.nextInt(m)] = rand.nextDouble();
  ProjOnPolyHSet proj = new ProjOnPolyHSet(HCModel.toVector(P), mlic.A, mlic.b, rand);
  P = HCModel.toMatrix(m, proj.optimum);
  mm = new HCModel[nd];
  for (int i = 0; i < nd; i++){
    mm[i] = new HCModel(n, bbs, cs[i], P, f[i].length);
  }
}
/**
 * restoration of previous P when a perturbation was not accepted
 */
void restore(){
  for (int i = 0; i < m; i++){
    for (int j = 0; j < m; j++){
      P[i][j] = buffP[i][j];
    }
  }
  mm = new HCModel[nd];
  for (int i = 0; i < nd; i++){
    mm[i] = new HCModel(n, bbs, cs[i], P, f[i].length);
  }
}
/**
 * initialization of global variables and random initialization of this.P
 */
void initModel(){
  P = new double[m][m];
  buffP = new double[m][m];
  mlic = new HCModelLIC(bbs);
  for (int i = 0; i < m; i++){
    for (int j = 0; j < m; j++){
      P[i][j] = rand.nextDouble();
    }
  }
  ProjOnPolyHSet proj = new ProjOnPolyHSet(HCModel.toVector(P), mlic.A, mlic.b, rand);
  P = HCModel.toMatrix(m, proj.optimum);
  mm = new HCModel[nd];
  for (int i = 0; i < nd; i++){
    mm[i] = new HCModel(n, bbs, cs[i], P, f[i].length);
  }
  for (int i = 0; i < m; i++){
```

```
    for (int j = 0; j < m; j++){
      buffP[i][j] = P[i][j];
    }
  }
}
/**
 * objective function (L1 distance between modeled and experimental fragment
 * length distributions)
 * @return objective function (L1 distance between modeled and experimental
 * fragment length distributions)
 */
double getE(){
  double res = 0.;
  for (int i = 0; i < nd; i++){
    double d = 0.;
    for (int j = 0; j < mm[i].lm; j++){
      d += Math.abs(mm[i].h[j]-f[i][j]);
    }
    res += d;
  }
  return res;
}
/**
 * reads experimental fragment length distributions from two files
 * @param file digest constraint files
 */
void loadConstraints(String[] file){
  f = new double[nd][];
  for (int i = 0; i < nd; i++){
    Vector<String> v = new Vector<String>();
    try{
      FileReader fr = new FileReader(file[i]);
      BufferedReader br = new BufferedReader(fr);
      String line = br.readLine();
      while((line = br.readLine()) != null){
        StringTokenizer st = new StringTokenizer(line, "\t");
        if (st.countTokens() == 2){
          v.add(line);
        }
      }
      br.close();
      fr.close();
    }
    catch(Exception ex){
      ex.printStackTrace();
    }
    f[i] = new double[v.size()];
    for (int j = 0; j < v.size(); j++){
      String line = v.elementAt(j);
      StringTokenizer st = new StringTokenizer(line, "\t");
      Integer L = new Integer(st.nextToken());
      Double D = new Double(st.nextToken());
      f[i][L.intValue()-1] = D.doubleValue();
    }
  }
```

```
}
/**
 * For supplementary material
 * @param inDir input directory (ends with"\\")
 * @param outDir output directory (ends with"\\")
 */
public static void varyingN(String inDir, String outDir){
  Random rand = new Random(1);
  int ns = 20;
  Vector<String> v = new Vector<String>();
  v.add("N\tminE\tavE\tmaxE");
  BBSet bbs = new BBSet(inDir+"US.ab.txt");
  for (int n = 12; n <= 20; n++){
    Integer I = new Integer(n);
    double avE = 0.;
    double minE = 10.;
    double maxE = 0.;
    for (int s = 1; s <= ns; s++){
      Integer S = new Integer(s);
      String pref = "N"+I.toString()+"s"+S.toString();
      String[] specFile = new String[2];
      specFile[0] = inDir+"US.hepI.txt";
      specFile[1] = inDir+"US.hepIII.txt";
      String[] consFile = new String[2];
      consFile[0] = inDir+"hepI.f.txt";
      consFile[1] = inDir+"hepIII.f.txt";
      String[] outFile = new String[2];
      outFile[0] = outDir+"MM."+pref+".hepI.fit.res";
      outFile[1] = outDir+"MM."+pref+".hepIII.fit.res";
      String modFile = outDir+"MM."+pref+".P.res";
      HCModelSA sa = new HCModelSA(n, bbs, specFile, consFile, outFile, modFile, rand);
      avE += sa.bestE;
      if (sa.bestE < minE){
        minE = sa.bestE;
      }
      if (sa.bestE > maxE){
        maxE = sa.bestE;
      }
    }
    avE /= (double) ns;
    String t = I.toString();
    Double D = new Double(minE);
    t += "\t"+D.toString();
    D = new Double(avE);
    t += "\t"+D.toString();
    D = new Double(maxE);
    t += "\t"+D.toString();
    v.add(t);
  }
  Utils.saveFile(v, outDir+"HCMandN.res");
}
/**
 * generates data used for figure of H&C fit
 * @param inDir input directory (ends with "\\")
 * @param outDir output directory (ends with "\\")
```

```
   */
  public static void defaultN(String inDir, String outDir){
    Random rand = new Random(1);
    BBSet bbs = new BBSet(inDir+"US.ab.txt");
    int n = 16;
    for (int s = 1; s <= 100; s++){
      Integer S = new Integer(s);
      String pref = "s"+S.toString();
      String[] specFile = new String[2];
      specFile[0] = inDir+"US.hepI.txt";
      specFile[1] = inDir+"US.hepIII.txt";
      String[] consFile = new String[2];
      consFile[0] = inDir+"hepI.f.txt";
      consFile[1] = inDir+"hepIII.f.txt";
      String[] outFile = new String[2];
      outFile[0] = outDir+"HC."+pref+".hepI.fit.res";
      outFile[1] = outDir+"HC."+pref+".hepIII.fit.res";
      String modFile = outDir+"MM."+pref+".P.res";
      new HCModelSA(n, bbs, specFile, consFile, outFile, modFile, rand);
    }
  }
  /**
   *
   * @param args
   */
  public static void main(String[] args){

    String inDir = "input\\";
    String outDir = "output\\";
    defaultN(inDir, outDir);
    varyingN(inDir, outDir);
  }
}
```

```
import java.io.BufferedWriter;
import java.io.FileWriter;
import java.util.Random;
import java.util.Vector;
/**
 * homogeneity and independence: species abundances are defined by
 * overall disaccharide composition
 */
public class HIModel {
  /**
   * number of building blocks
   */
  int m = 0;
  /**
   * chain length
   */
  int n = 0;
  /**
   * overall cleavage probability
   */
  double c = 0;
  /**
   * set of building blocks
   */
  BBSet bbs = null;
  /**
   * cleavage specificities
   */
  CSpec cs = null;
  /**
   * distribution of fragment length
   */
  double[] g = null;
  /**
   * distribution of fragment length with upper bound lm
   */
  double[] h = null;
  /**
   * upper bound for fragment length
   */
  int lm = 0;
  /**
   * cumulative version of overall building-block proportions,
   * utilized for MC simulation
   */
  double[] rhoF = null;
  /**
   * Computes heparinase fragment length distribution under H&I (this.g)
   * @param n BKHS chain length
   * @param bbs set of building blocks (disaccharides and their overall proportions)
   * @param cs cleavage specificity/yield (one heparinase only)
   * @param lm maximum fragment length (cumulative for h(l) when l+1 >= lm)
   */
```

```
public HIModel(int n, BBSet bbs, CSpec cs, int lm){
  this.lm = lm;
  this.bbs = bbs;
  this.cs = cs;
  m = bbs.m;
  rhoF = new double[m];
  rhoF[0] = bbs.rho[0];
  for (int i = 1; i < m; i++){
    rhoF[i] = rhoF[i-1]+bbs.rho[i];
  }
  this.n = n;
  makeC();
  makeG();
  makeH();
}
/**
 * numerical check via simulations
 * @param rand
 * @param file output file
 */
void checkGL(Random rand, String file){
  int nsim = 10000000;
  int totfrag = 0;
  double[] g = new double[n];
  for (int sim = 0; sim < nsim; sim++){
    CleavedSequence cseq = getCleavedSequence(rand);
    int[][] frag = cseq.getFragments();
    totfrag += frag.length;
    for (int i = 0; i < frag.length; i++){
      g[frag[i].length-1] += 1.;
    }
  }
  for (int i = 0; i < g.length; i++){
    g[i] /= (double) totfrag;
  }
  double[] h = new double[n];
  for (int i = 0; i < lm; i++){
    h[i] = g[i];
  }
  for (int i = lm; i < n; i++){
    h[lm-1] += g[i];
  }
  Vector<String> v = new Vector<String>();
  v.add("l\tgl\tsimgl\thl\tsimhl");
  for (int i = 0; i < n; i++){
    Integer L = new Integer(i+1);
    String outS = L.toString();
    Double D = new Double(this.g[i]);
    outS += "\t"+D.toString();
    D = new Double(g[i]);
    outS += "\t"+D.toString();
    D = new Double(this.h[i]);
    outS += "\t"+D.toString();
    D = new Double(h[i]);
    outS += "\t"+D.toString();
```

```
    v.add(outS);
  }
  Utils.saveFile(v, file);
}
/**
 * returns a random chain sequence before cleavage
 * @param rand
 * @return a random chain sequence before cleavage
 */
int[] getSequence(Random rand){

  int[] res = new int[n];
  for (int i = 0; i < n; i++){
    res[i] = Utils.getRandIndexInF(rhoF, rand);
  }
  return res;
}
/**
 * generates a random set of cleavage positions in sequence seq based
 * on cleavage specificities (always a cut at position n)
 * @param seq BKHS sequence
 * @param rand
 * @return a random set of cleavage positions in sequence seq based on
 * cleavage specificities
 */
int[] getCuts(int[] seq, Random rand){
  Vector<Integer> cuts = new Vector<Integer>();
  for (int i = 1; i < n; i++){
    double d = rand.nextDouble();
    if (d < cs.c[seq[i]]){
      cuts.add(new Integer(i));
    }
  }
  cuts.add(new Integer(n));
  int[] res = new int[cuts.size()];
  for (int i = 0; i < cuts.size(); i++){
    Integer I = cuts.elementAt(i);
    res[i] = I.intValue();
  }
  return res;
}
/**
 * generates a random CleavedSequence (generates a BKHS chain and randomly
 * cleaves it based on cleavage specificities);  see methods getFragments()
 * of class CleavedSequence to access resulting fragments
 * @param rand
 * @return a random CleavedSequence (generates a BKHS chain and randomly cleaves
 * it based on cleavage specificities)
 */
CleavedSequence getCleavedSequence(Random rand){
  int[] seq = null;
  int[] cuts = new int[1];
  while(cuts.length < 2){ // only fragments obtained by cleavage are visible
    seq = getSequence(rand);
    cuts = getCuts(seq, rand);
```

```
  }
  return new CleavedSequence(seq, cuts);
}
/**
 * version this.h of this.g (fragment length distribution):
 *  abundances for ll >= lm are summed
 */
void makeH(){
  h = new double[n];
  for (int l = 0; l < lm; l++){
    h[l] = g[l];
  }
  for (int l = lm; l < n-1; l++){
    h[lm-1] += g[l];
  }
}
/**
 * distribution of fragment length (this.g) expected under H&I
 */
void makeG(){
  g = new double[n];
  for (int ll = 1; ll <= n-1; ll++){
    int l = ll-1;
    g[l] = 1.+c*(double)(n-ll-1);
    g[l] /= (double)(n-1);
    g[l] *= Math.pow((1.-c), (double) l);
  }
}
/**
 * computes parameter this.c used to compute this.g
 */
void makeC(){
  c = 0.;
  for (int i = 0; i < m; i++){
    c += bbs.rho[i]*cs.c[i];
  }
}
/**
 * saves this.h
 * @param file output file
 */
void saveH(String file){
  try{
    FileWriter fw = new FileWriter(file);
    BufferedWriter bw = new BufferedWriter(fw);
    bw.write("l\th");
    bw.newLine();
    for (int i = 0; i < lm; i++){
      Integer L = new Integer(i+1);
      Double D = new Double(h[i]);
      bw.write(L.toString()+"\t"+D.toString());
      bw.newLine();
    }
    bw.close();
    fw.close();
```

```
    }
    catch(Exception ex){
      ex.printStackTrace();
    }
  }
  /**
   * saves fragment length distribution expected under H&I for hepI
   * and performs numerical check
   * @param inDir input directory (ends with "\\")
   * @param outDir output directory (ends with "\\")
   */
  public static void hepI(String inDir, String outDir){
    Random rand = new Random();
    String file = inDir+"US.ab.txt";
    BBSet bbs = new BBSet(file);
    file = inDir+"US.hepI.txt";
    CSpec cs = new CSpec(file, bbs);
    int n = 16;
    HIModel pm = new HIModel(n, bbs, cs, 11);
    pm.checkGL(rand, outDir+"HIM.hepIgl.check.res");
    pm.saveH(outDir+"HIM.hepIhl.res");
  }
  /**
   * saves fragment length distribution expected under H&I for hepIII
   * and performs numerical check
   * @param inDir input directory (ends with "\\")
   * @param outDir output directory (ends with "\\")
   */
  public static void hepIII(String inDir, String outDir){
    Random rand = new Random();
    String file = inDir+"US.ab.txt";
    BBSet bbs = new BBSet(file);
    file = inDir+"US.hepIII.txt";
    CSpec cs = new CSpec(file, bbs);
    int n = 16;
    HIModel pm = new HIModel(n, bbs, cs, 6);
    pm.checkGL(rand, outDir+"HIM.hepIIIgl.check.res");
    pm.saveH(outDir+"HIM.hepIIIhl.res");
  }
  /**
   *
   * @param args
   */
  public static void main(String[] args){
    String inDir = "input\\";
    String outDir = "output\\";
    hepI(inDir, outDir);
    hepIII(inDir, outDir);
  }
}
```

```
import java.util.Vector;
/**
 * Feasibility problem (phase I via simplex) to test for the possibility
 * of homogeneous disaccharide composition along BKHS chains when combined
 * with heparinase digest constraints
 */
public class HomogeneityFeasibility {
  /**
   * enumeration of BKHS chain sequences
   */
  Species sp = null;
  /**
   * matrix in constraing Ap = b
   */
  double[][] A = null;
  /**
   * vector in constraint Ap = b
   */
  double[] b = null;
  /**
   * convenience wrapper for constraint Ap = b
   */
  LinEqCons lec = null;
  /**
   * solution to feasibility problem (phase I) via linear programming
   * (simplex method)
   */
  SimplexPhaseI sp1 = null;
  /**
   * output of the feasibility problem: close to 0 if homogeneity is feasible
   */
  double infeasibility = 0.;
  /**
   * disaccharide labels
   */
  String[] lab = null;
  /**
   * Feasibility problem (phase I via simplex) to test for the possibility
   * of homogeneous disaccharide composition along BKHS chains when combined with
   * heparinase digest constraints
   * @param m number of disaccharides
   * @param n BKHD chain length
   * @param lab disaccharide labels
   * @param inDir input directory (ends with "\\")
   */
  public HomogeneityFeasibility(int m, int n, String[] lab, String inDir){
    this.lab = lab;
    sp = new Species(m, n);
    BBSet bbs = new BBSet(inDir+"US.ab.txt");
    String[] csFile = new String[2];
    String[] fragFile = new String[2];
    csFile[0] = new String(inDir+"US.hepI.txt");
    csFile[1] = new String(inDir+"US.hepIII.txt");
```

```
    fragFile[0] = new String(inDir+"hepI.f.txt");
    fragFile[1] = new String(inDir+"hepIII.f.txt");
    Vector<LinEqCons> v = new Vector<LinEqCons>();
    v.add(sp.getNormLEC());
    v.add(LinEqCons.removeLastRow(sp.getFragLEC(Species.loadFragAbund(fragFile[0]),
        new CSpec(csFile[0], bbs), bbs)));
    v.add(LinEqCons.removeLastRow(sp.getFragLEC(Species.loadFragAbund(fragFile[1]),
        new CSpec(csFile[1], bbs), bbs)));
    v.add(sp.getHomogeneityLEC(bbs));
    lec = new LinEqCons(v);
    A = lec.A;
    b = lec.b;
    sp1 = new SimplexPhaseI(A, b);
    infeasibility = sp1.finalCost;
    System.err.println(n+"\t"+infeasibility);
  }
  /**
   * computes feasibility of combining homogeneity and heparinase digest constraints
   * for BKHS chain lengths between 5 and 20
   * @param inDir input directory (ends with "\\")
   * @param outDir output directory (ends with "\\")
   */
  public static void makeProfile(String inDir, String outDir){
    String[] lab = new String[2];
    lab[0] = new String("U");
    lab[1] = new String("S");
    Vector<String> v = new Vector<String>();
    v.add("n\tinfeasibility");
    for (int n = 5; n <= 20; n++){
      HomogeneityFeasibility hf = new HomogeneityFeasibility(2,n,lab,inDir);
      Integer N = new Integer(n);
      Double D = new Double(hf.infeasibility);
      v.add(N.toString()+"\t"+D.toString());
    }
    Utils.saveFile(v, outDir+"HomogeneityFeasibility.res");
  }
  /**
   *
   * @param args
   */
  public static void main(String[] args){
    String inDir = "input\\";
    String outDir = "output\\";
    makeProfile(inDir, outDir);
  }
}
```

```
import java.util.Vector;
/**
 * Class that represents linear equality/inequality constraints: Ax = b or Ax <= b,
 * where A is an m by n matrix and b an m vector. The main utility of this class
 * is merging of linear constraints
 */
public class LinEqCons {
  /**
   * number of rows
   */
  int m = 0;
  /**
   * number of columns
   */
  int n = 0;
  /**
   * matrix in Ax = b
   */
  public double[][] A = null;
  /**
   * vector in Ax = b
   */
  public double[] b = null;
  /**
   * label for each row (type of constraint)
   */
  public String[] type = null;
  /**
   * represents a linear equality constraint: Ax = b
   * @param A matrix in Ax = b
   * @param b vector in Ax = b
   * @param label row labels
   */
  public LinEqCons(double[][] A, double[] b, String[] label){
    m = A.length;
    n = A[0].length;
    this.A = new double[m][n];
    this.b = new double[m];
    type = new String[m];
    for (int i = 0; i < m; i++){
      this.b[i] = b[i];
      type[i] = new String(label[i]);
      for (int j = 0; j < n; j++){
        this.A[i][j] = A[i][j];
      }
    }
  }
  /**
   * Merges several linear constraints (array) into one
   * @param lec array of linear constraints
   */
  public LinEqCons(LinEqCons[] lec){
    m = 0;
```

```
  n = lec[0].n;
  for (int k = 0; k < lec.length; k++){
    m += lec[k].m;
  }
  A = new double[m][n];
  b = new double[m];
  type = new String[m];
  int i = -1;
  for (int k = 0; k < lec.length; k++){
    for (int l = 0; l < lec[k].m; l++){
      i++;
      b[i] = lec[k].b[l];
      type[i] = new String(lec[k].type[l]);
      for (int j = 0; j < lec[k].n; j++){
        A[i][j] = lec[k].A[l][j];
      }
    }
  }
}
/**
 * Merges several linear constraints (Vector<LinEqCons>) into one
 * @param lec Vector containing linear constraints
 */
public LinEqCons(Vector<LinEqCons> lec){
  m = 0;
  LinEqCons lc = lec.elementAt(0);
  n = lc.n;
  for (int k = 0; k < lec.size(); k++){
    lc = lec.elementAt(k);
    m += lc.m;
  }
  A = new double[m][n];
  b = new double[m];
  type = new String[m];
  int i = -1;
  for (int k = 0; k < lec.size(); k++){
    lc = lec.elementAt(k);
    for (int l = 0; l < lc.m; l++){
      i++;
      b[i] = lc.b[l];
      type[i] = new String(lc.type[l]);
      for (int j = 0; j < lc.n; j++){
        A[i][j] = lc.A[l][j];
      }
    }
  }
}
/**
 * Returns a String representation of this LinEqCons:
 * type \t b \t A[][0] \t A[][1] \t ... \t A[][n-1]
 * @return a String representation of this LinEqCons
 */
public String getStringRep(){
  String res = "";
  for (int i = 0; i < m; i++){
```

```
    res += type[i];
    Double D = new Double(b[i]);
    res += "\t" + D.toString();
    for (int j = 0; j < n; j++){
      D = new Double(A[i][j]);
      res += "\t"+D.toString();
    }
    res += "\n";
  }
  return res;
}
/**
 * transforms Ax = b into Ax <= b and -Ax <= -b
 * @param lec set of equality constraints
 * @return inequality version of Ax = b (Ax <= b and -Ax <= -b)
 */
public static LinEqCons equalitiesToInequalities(LinEqCons lec){
  int n = lec.n;
  int m = 2*lec.m;
  double[][] A = new double[m][n];
  double[] b = new double[m];
  String[] tp = new String[m];
  int row = -1;
  for (int i = 0; i < lec.m; i++){
    row++;
    b[row] = lec.b[i];
    tp[row] = new String(lec.type[i]);
    for (int j = 0; j < n; j++){
      A[row][j] = lec.A[i][j];
    }
  }
  for (int i = 0; i < lec.m; i++){
    row++;
    b[row] = -lec.b[i];
    tp[row] = new String(lec.type[i]);
    for (int j = 0; j < n; j++){
      A[row][j] = -lec.A[i][j];
    }
  }
  return new LinEqCons(A, b, tp);
}
/**
 * removes one row (constraint) in lec
 * @param lec linear constraint
 * @return lec minus its last row (constraint)
 */
public static LinEqCons removeLastRow(LinEqCons lec){
  int m = lec.m-1;
  int n = lec.n;
  double[][] A = new double[m][n];
  double[] b = new double[m];
  String[] lab = new String[m];
  for (int i = 0; i < m; i++){
    b[i] = lec.b[i];
    lab[i] = new String(lec.type[i]);
```

```
      for (int j = 0; j < n; j++){
        A[i][j] = lec.A[i][j];
      }
    }
    return new LinEqCons(A, b, lab);
  }
}
```

```
/**
 * This class contains a few static methods for matrix and vector operations.
 */
public class MatrixOp {
  /**
   * Prints out a matrix
   * @param mat matrix
   */
  public static void printMat(double[][] mat){
        String s = null;
        Double D = null;
        System.out.println("*************");
        for (int i = 0; i < mat.length; i++){
            s = "";
            for (int j = 0; j < mat[0].length; j++){
                D = new Double(mat[i][j]);
                s += D.toString();
                if (j != mat[0].length-1)
                    s += "\t";
            }
            System.out.println(s);
        }
        System.out.println("*************");
    }
  /**
   * Prints out a matrix, with "." for 0 and "x" for entries not 0
   * @param mat matrix
   * @param eps definition of 0
   */
  public static void printMatS(double[][] mat, double eps){
        String s = null;
        System.out.println("*************");
        for (int i = 0; i < mat.length; i++){
            s = "";
            for (int j = 0; j < mat[0].length; j++){
              String e = "x";
              if (Math.abs(mat[i][j]) <= eps){
                e = ".";
              }
                s += e;
                if (j != mat[0].length-1)
                    s += "\t";
            }
            System.out.println(s);
        }
        System.out.println("*************");
    }
  /**
   * Returns a String representation of a matrix
   * @param mat matrix
   * @return a String representation of matrix 'mat'
   */
  public static String getMatStringRep(double[][] mat){
```

```
      Double D = null;
      String res = "*************\n";
      for (int i = 0; i < mat.length; i++){
          for (int j = 0; j < mat[0].length; j++){
              D = new Double(mat[i][j]);
              res += D.toString();
              if (j != mat[0].length-1)
                  res += "\t";
          }
          res += "\n";
      }
      res += "*************\n";
      return res;
  }
/**
 * Returns a String representation of a vector
 * @param x vector
 * @return String representation of vector 'x'
 */
public static String getVecStringRep(double[] x){
  String res = "*************\n";
  for (int i = 0; i < x.length; i++){
    Double D = new Double(x[i]);
      res += D.toString()+"\n";
  }
  res += "*************\n";
  return res;
}
/**
 * Prints out a vector
 * @param x vector
 */
public static void printVec(double[] x){
  System.out.println("*************");
  for (int i = 0; i < x.length; i++){
      System.out.println(x[i]);
  }
  System.out.println("*************");
}
/**
 * Returns the transpose of a matrix
 * @param a matrix
 * @return transpose of a
 */
public static double[][] transpose(double[][] a){
  double[][] at = new double[a[0].length][a.length];
  for (int i = 0; i < a.length; i++){
      for (int j = 0; j < a[i].length; j++){
        at[j][i] = a[i][j];
      }
  }
  return at;
}
/**
 * Returns the L2 norm square of a vector
```

```
 * @param x vector
 * @return L2 norm square of vector x
 */
public static double getL2Norm(double[] x){
  double res = 0.;
  for (int i = 0; i < x.length; i++){
    res += (x[i]*x[i]);
  }
  return res;
}
/**
 * Returns c = ab, where a is a matrix and b a vector
 * @param a matrix
 * @param b vector
 * @return product ab
 */
public static double[] multMatVec(double[][] a, double[] b){
  double[] c = new double[a.length];
  for (int i = 0; i < c.length; i++){
      c[i] = 0.;
      for (int j = 0; j < a[i].length; j++){
        c[i] += (a[i][j]*b[j]);
      }
  }
  return c;
}
/**
 * Returns the product of two matrices
 * @param a matrix
 * @param b matrix
 * @return c = ab
 */
public static double[][] multMat(double[][] a, double[][] b){
  double[][] c = new double[a.length][b[0].length];
  for (int i = 0; i < c.length; i++){
            for (int j = 0; j < c[0].length; j++){
                c[i][j] = 0.;
                for (int k = 0; k < a[0].length; k++)
                    c[i][j] += (a[i][k]*b[k][j]);
            }
        }
  return c;
}
/**
 * Performs the Cholesky factorization of a symmetric and positive
 * definite matrix A: A = LL^T, where L is lower triangular with positive
 * diagonal elements. This method returns L
 * @param A input matrix (symmetric and positive definite)
 * @return Cholesky factor L of A = LL^T
 */
public static double[][] cholesky(double[][] A){
  int m = A.length;
  double[][] l = new double[m][m];
  for (int k = 0; k < m; k++){
      l[k][k] = A[k][k];
```

```
      for (int j = 0; j < k; j++){
        l[k][k] -= (l[k][j]*l[k][j]);
      }
      l[k][k] = Math.sqrt(l[k][k]);
      for (int i = k+1; i < m; i++){
        l[i][k] = A[i][k];
        for (int j = 0; j < k; j++){
          l[i][k] -= (l[i][j]*l[k][j]);
        }
        l[i][k] /= l[k][k];
      }
  }
  return l;
}
/**
 * Solves the linear system ax = b, where a is lower triangular
 * @param a matrix
 * @param b vector
 * @return solution x to ax = b
 */
public static double[] forwardElimination(double[][] a, double[] b){
  int m = b.length;
  double[] x = new double[m];
  for (int i = 0; i < m; i++){
      x[i] = b[i];
      for (int j = 0; j < i; j++){
        x[i] -= (a[i][j]*x[j]);
      }
      x[i] /= a[i][i];
  }
  return x;
}
/**
 * Solves the system ax = b, where a is upper triangular
 * @param a matrix
 * @param b vector
 * @return solution x to ax = b
 */
public static double[] backwardSubstitution(double[][] a, double[] b){
  int m = b.length;
  double[] x = new double[m];
  for (int i = m-1; i >= 0; i--){
      x[i] = b[i];
      for (int j = i+1; j < m; j++){
        x[i] -= (a[i][j]*x[j]);
      }
      x[i] /= a[i][i];
  }
  return x;
}
/**
 * Solves the linear system ax = b, where matrix a is symmetric and
 * positive definite
 * @param a matrix
 * @param b vector
```

```
   * @return solution x to ax = b
   */
  public static double[] choleskySolve(double[][] a, double[] b){
    double[][] l = cholesky(a);
    double[][] lt = transpose(l);
    double[] y = forwardElimination(l, b);
    double[] x = backwardSubstitution(lt, y);
    return x;
  }
  /**
   * Computes the inner product between two vectors
   * @param x vector of dim n
   * @param y vector of dim n
   * @return sum_{i=1}^n x_iy_i
   */
  public static double geInnerProd(double[] x, double[] y){
    double res = 0.;
    for (int i = 0; i < x.length; i++){
      res += (x[i]*y[i]);
    }
    return res;
  }
}
```

```
import java.io.BufferedWriter;
import java.io.FileWriter;
import java.util.Arrays;
import java.util.Enumeration;
import java.util.Hashtable;
import java.util.StringTokenizer;
import java.util.Vector;
/**
 * represents a MaxEnt model of BKHS (profiles of composition and correlation)
 * when all chains have same length
 */
public class MaxEntModel {
  /**
   * solution (species abundances p) via geometric programming
   */
  MaxEntOptim opt = null;
  /**
   * chain length
   */
  int n = 0;
  /**
   * number of disaccharides
   */
  int m = 0;
  /**
   * composition profile
   */
  double[][] gamma = null;
  /**
   * transition probabilities averaged over all positions, P[u][s]: from u to s
   */
  double[][] P = null;
  /**
   * transition probabilities, pt[i][u][s]: from u at position i to s at i+1
   */
  double[][][] pt = null;
  /**
   * disaccharides and overall proportions
   */
  BBSet bbs = null;
  /**
   * sequences of species
   */
  Species species = null;
  /**
   * numerical check
   */
  double sump = 0.;
  /**
   * prefix for output files
   */
  String outPref = null;
  /**
```

```
 * represents a MaxEnt model of BKHS (profiles of composition and correlation)
 * when all chains have same length
 * @param op MaxEnt model of individual species abundances
 * @param n BKHS chain length
 * @param bbs disaccharides
 * @param outF prefix for output files
 */
public MaxEntModel(MaxEntOptim op, int n, BBSet bbs, String outF){
  outPref = new String(outF);
  opt = op;
  this.n = n;
  this.bbs = bbs;
  m = bbs.m;
  species = new Species(m,n);
  sump = 0.;
  for (int s = 0; s < opt.n; s++){
    sump += opt.p[s];
  }
  try{
    FileWriter fw = new FileWriter(outPref+".pind.res");
    BufferedWriter bw = new BufferedWriter(fw);
    for (int i = 0; i < species.N; i++){
      Double D = new Double(opt.p[i]);
      String s = D.toString();
      for (int j = 0; j < species.seq[i].length; j++){
        Integer J = new Integer(species.seq[i][j]);
        s += "\t"+J.toString();
      }
      bw.write(s);
      bw.newLine();
    }
    bw.close();
    fw.close();
  }
  catch(Exception ex){
    ex.printStackTrace();
  }
  makeGamma();
  makePT();
  makeP();
}
/**
 * estimates transition probabilities averaged over all positions
 */
void makeP(){
  P = new double[m][m];
  for (int i = 0; i < m; i++){
    for (int j = 0; j < m; j++){
      P[i][j] = 0.;
      for (int pos = 1; pos < n; pos++){
        P[i][j] += pt[pos][i][j];
      }
      P[i][j] /= (double) (n-1);
    }
  }
```

```
    Vector<String> v = new Vector<String>();
    String s = "prev\tnext\tp";
    v.add(s);
    for (int i = 0; i < m; i++){
      for (int j = 0; j < m; j++){
        Double D = new Double(P[i][j]);
        s = bbs.name[i]+"\t"+bbs.name[j]+"\t"+D.toString();
        v.add(s);
      }
    }
    Utils.saveFile(v, outPref+".P.res");
  }
  /**
   * estimates transition probabilities at each position
   */
  void makePT(){
    pt = new double[n][m][m];
    for (int s = 0; s < species.N; s++){
      for (int i = 0; i < m; i++){
        pt[0][i][species.seq[s][0]] += opt.p[s];
      }
    }
    for (int i = 0; i < m; i++){
      double sum = 0.;
      for (int j = 0; j < m; j++){
        sum += pt[0][i][j];
      }
      for (int j = 0; j < m; j++){
        pt[0][i][j] /= sum;
      }
    }
    for (int pos = 1; pos < n; pos++){
      for (int s = 0; s < species.N; s++){
        pt[pos][species.seq[s][pos-1]][species.seq[s][pos]] += opt.p[s];
      }
      for (int i = 0; i < m; i++){
        double sum = 0.;
        for (int j = 0; j < m; j++){
          sum += pt[pos][i][j];
        }
        for (int j = 0; j < m; j++){
          pt[pos][i][j] /= sum;
        }
      }
    }
    Vector<String> v = new Vector<String>();
    String s = "pos";
    for (int i = 0; i < m; i++){
      for (int j = 0; j < m; j++){
        s += "\tp"+bbs.name[i]+bbs.name[j];
      }
    }
    v.add(s);
    for (int pos = 0; pos < n; pos++){
      Integer I = new Integer(pos+1);
```

```
    s = I.toString();
    for (int i = 0; i < m; i++){
      for (int j = 0; j < m; j++){
        Double D = new Double(pt[pos][i][j]);
        s += "\t"+D.toString();
      }
    }
    v.add(s);
  }
  Utils.saveFile(v, outPref+".pt.res");
}
/**
 * estimates profile of S/U composition
 */
void makeGamma(){
  gamma = new double[n][m];
  for (int s = 0; s < opt.n; s++){
    for (int i = 0; i < n; i++){
      gamma[i][species.seq[s][i]] += opt.p[s];
    }
  }
  Vector<String> v = new Vector<String>();
  String s = "pos";
  for (int i = 0; i < m; i++){
    s += "\t"+bbs.name[i];
  }
  v.add(s);
  for (int pos = 0; pos < n; pos++){
    Integer I = new Integer(pos+1);
    s = I.toString();
    for (int i = 0; i < m; i++){
      Double D = new Double(gamma[pos][i]);
      s += "\t"+D.toString();
    }
    v.add(s);
  }
  Utils.saveFile(v, outPref+".gamma.res");
}
/**
 * saves all individual species abundances
 * @param file output file
 */
void saveSpeciesAbundance(String file){
  Vector<String> v = new Vector<String>();
  v.add("species\tp");
  for (int i = 0; i < species.seq.length; i++){
    String s = "";
    for (int j = 0; j < species.seq[i].length; j++){
      s += bbs.name[species.seq[i][j]];
    }
    Double D = new Double(opt.p[i]);
    v.add(s+"\t"+D.toString());
  }
  Utils.saveFile(v, file);
}
```

```
/**
 * default linear equality constraint = composition-1 + 2(digest-1)
 * @return default linear equality constraint = composition-1 + 2(digest-1)
 */
public static LinEqCons getDefaultLEC(String inDir){
  Species sp = new Species(2, 16);
  BBSet bbs = new BBSet(inDir+"US.ab.txt");
  Vector<LinEqCons> v = new Vector<LinEqCons>();
  v.add(LinEqCons.removeLastRow(sp.getCompLEC(bbs.rho)));
  v.add(LinEqCons.removeLastRow(sp.getFragLEC(Species.loadFragAbund(inDir+"hepI.f.txt"),
      new CSpec(inDir+"US.hepI.txt", bbs), bbs)));
  v.add(LinEqCons.removeLastRow(sp.getFragLEC(Species.loadFragAbund(inDir+"hepIII.f.txt"),
      new CSpec(inDir+"US.hepIII.txt", bbs), bbs)));
  return new LinEqCons(v);
}
/**
 * MaxEnt models for chain length = 13, 14, 15, 16, 17 or 18
 * @param inDir input directory (ends with "\\")
 * @param outDir output directory (ends with "\\")
 */
public static void defaultModelAndN(String inDir, String outDir){
  BBSet bbs = new BBSet(inDir+"US.ab.txt");
  for (int n = 13; n <= 18; n++){
    Integer I = new Integer(n);
    Species sp = new Species(2, n);
    Vector<LinEqCons> v = new Vector<LinEqCons>();
    v.add(LinEqCons.removeLastRow(sp.getCompLEC(bbs.rho)));
    v.add(LinEqCons.removeLastRow(sp.getFragLEC(Species.loadFragAbund(inDir+"hepI.f.txt"),
        new CSpec(inDir+"US.hepI.txt", bbs), bbs)));
    v.add(LinEqCons.removeLastRow(sp.getFragLEC(Species.loadFragAbund(inDir+"hepIII.f.txt"),
        new CSpec(inDir+"US.hepIII.txt", bbs), bbs)));
    LinEqCons lec = new LinEqCons(v);
    MaxEntOptim opt = new MaxEntOptim(lec.A, lec.b);
    new MaxEntModel(opt, n, bbs, outDir+"MEMN"+I.toString());
  }
}
/**
 * saves maxent S/U composition at each position when all chains have same length n = 16
 * @param inDir input directory (ends with "\\")
 * @param outDir output directory (ends with "\\")
 */
public static void defaultModel(String inDir, String outDir){
  BBSet bbs = new BBSet(inDir+"US.ab.txt");
  LinEqCons lec = getDefaultLEC(inDir);
  MaxEntOptim opt = new MaxEntOptim(lec.A, lec.b);
  new MaxEntModel(opt, 16, bbs, outDir+"MEM");
}
/**
 * saves individual species abundances when all chains have same length n = 16
 * @param inDir input directory (ends with "\\")
 * @param outDir output directory (ends with "\\")
 */
public static void defaultModelSpecies(String inDir, String outDir){
  BBSet bbs = new BBSet(inDir+"US.ab.txt");
  LinEqCons lec = getDefaultLEC(inDir);
```

```
    MaxEntOptim opt = new MaxEntOptim(lec.A, lec.b);
    MaxEntModel mem = new MaxEntModel(opt, 16, bbs, outDir+"MEMspec");
    mem.saveSpeciesAbundance(outDir+"MEMspec.species.res");
  }
  /**
   * saves species abundances after removing the non-reducing end
   * @param inDir input directory (ends with "\\")
   * @param outDir output directory (ends with "\\")
   */
  public static void length15Abundances(String inDir, String outDir){
    Vector<String> v = Utils.loadFileNoheader(outDir+"MEMspec.species.res");
    Hashtable<String,Double> s2p = new Hashtable<String,Double>();
    for (int i = 0; i < v.size(); i++){
      StringTokenizer st = new StringTokenizer(v.elementAt(i), "\t");
      String s = st.nextToken().trim();
      s = s.substring(1, s.length());
      Double D = new Double(st.nextToken());
      Double p = s2p.get(s);
      if (p == null){
        p = new Double(0.);
      }
      p = new Double(p.doubleValue()+D.doubleValue());
      s2p.put(s, p);
    }
    SandVal[] sv = new SandVal[s2p.size()];
    Enumeration<String> en = s2p.keys();
    int c = -1;
    while(en.hasMoreElements()){
      c++;
      String s = en.nextElement();
      Double p = s2p.get(s);
      sv[c] = new SandVal(s, p.doubleValue(), "d");
    }
    Arrays.sort(sv, sv[0]);
    BBSet bbs = new BBSet(inDir+"US.ab.txt");
    v = new Vector<String>();
    v.add("species\tp\tp2");
    for (int i = 0; i < sv.length; i++){
      Double D = new Double(sv[i].val);
      String seq = sv[i].s;
      Double p2 = getP2(seq, bbs);
      String seq2 = "x";
      for (int j = 0; j < seq.length(); j++){
        String t = seq.substring(j, j+1);
        if (t.equals("s")){
          seq2 += "{\\bf S}";
        }
        else{
          seq2 += "U";
        }
      }
      v.add(seq2+"\t"+D.toString()+"\t"+p2.toString());
    }
    Utils.saveFile(v, outDir+"MEMspec.species.L15.res");
  }
```

```
  /**
   * relative abundance of a species (seq) under the MaxEnt model defined by only
   * overall disaccharide composition constraint (model H&I)
   * @param seq species sequence
   * @param bbs disaccharide overall abundances
   * @return relative abundance of a species (seq) under the MaxEnt model defined
   * by only overall disaccharide composition constraint (model H&I)
   */
  public static double getP2(String seq, BBSet bbs){
    double res = 1.;
    for (int i = 0; i < seq.length(); i++){
      String s = seq.substring(i, i+1);
      Integer I = bbs.name2i.get(s);
      res *= bbs.rho[I.intValue()];
    }
    return res;
  }
  /**
   *
   * @param args
   */
  public static void main(String[] args){
    String inDir = "input\\";
    String outDir = "output\\";
    defaultModel(inDir, outDir);
    defaultModelAndN(inDir, outDir);
    defaultModelSpecies(inDir, outDir);
    length15Abundances(inDir,outDir);
  }
}
```

```
import java.io.BufferedReader;
import java.io.BufferedWriter;
import java.io.FileReader;
import java.io.FileWriter;
import java.util.StringTokenizer;
import java.util.Vector;
/**
 * MaxEnt model of profiles of S/U composition and transition probabilities
 * along BKHS chains when BKHS is represented with a mixture of chain lengths
 */
public class MaxEntModelW {
  /**
   * solution (individual species abundances p) via geometric programming
   */
  MaxEntOptim opt = null;
  /**
   * number of disaccharides
   */
  int m = 2;
  /**
   * transition probabilities averaged over all positions, P[u][s]: from u to s
   */
  double[][] P = null;
  /**
   * disaccharides and overall proportions
   */
  BBSet bbs = null;
  /**
   * mixture model of chain lengths and sequence enumeration
   */
  MixSpecies msp = null;
  /**
   * prefix for output files
   */
  String outPref = null;
  /**
   * MaxEnt model of profiles of S/U composition and transition probabilities
   * along BKHS chains when BKHS is represented with a mixture of chain lengths;
   * saves MaxEnt individual species abundances (*.pind.res file)
   * @param outF prefix for output files
   * @param lmin smallest chain length
   * @param lmax largest chain length
   * @param sigma spread of chain lengths
   * @param mu average chain length
   * @param inDir input directory
   */
  public MaxEntModelW(String outF, int lmin, int lmax, double sigma, double mu, String inDir){
    outPref = new String(outF);
    LinEqCons lec = getWLEC(lmin, lmax, sigma, mu, inDir);
    opt = new MaxEntOptim(lec.A, lec.b);
    try{
      FileWriter fw = new FileWriter(outPref+".pind.res");
      BufferedWriter bw = new BufferedWriter(fw);
```

```
    for (int i = 0; i < msp.N; i++){
      Double D = new Double(opt.p[i]);
      String s = D.toString();
      for (int j = 0; j < msp.seq[i].length; j++){
        Integer J = new Integer(msp.seq[i][j]);
        s += "\t"+J.toString();
      }
      bw.write(s);
      bw.newLine();
    }
    bw.close();
    fw.close();
  }
  catch(Exception ex){
    ex.printStackTrace();
  }
}
/**
 * default linear equality constraint with chain length distribution
 *  = composition-1 + 2(digest-1) + chain length distribution -1
 * @param lmin smallest chain length
 * @param lmax largest chain length
 * @param sigma chain length spread
 * @param mu average chain length
 * @param inDir input directory
 * @return default linear equality constraint with chain length distribution
 */
LinEqCons getWLEC(int lmin, int lmax, double sigma, double mu, String inDir){
  msp = new MixSpecies(lmin, lmax, sigma, mu);
  BBSet bbs = new BBSet(inDir+"US.ab.txt");
  Vector<LinEqCons> v = new Vector<LinEqCons>();
  v.add(LinEqCons.removeLastRow(msp.getRhoLEC(bbs)));
  v.add(LinEqCons.removeLastRow(msp.getFragLEC(Species.loadFragAbund(inDir+"hepI.f.txt"),
      new CSpec(inDir+"US.hepI.txt", bbs), bbs)));
  v.add(LinEqCons.removeLastRow(msp.getFragLEC(Species.loadFragAbund(inDir+"hepIII.f.txt"),
      new CSpec(inDir+"US.hepIII.txt", bbs), bbs)));
  v.add(LinEqCons.removeLastRow(msp.getWLEC()));
  return new LinEqCons(v);
}
/**
 * reads a file containing MaxEnt abundances of individual species
 * (pref.pind.res) and save files containing:
 * NRE and RE profiles of disaccharide composition (pref.prof.res)
 * disaccharide composition as a function of chain length (pref.rhol.res)
 * profile of transition probabilities when aligning by NRE (pref.ptnre.res)
 * profile of transition probabilities when aligning by RE (pref.ptre.res)
 * @param inDir input directory (ends with "\\")
 * @param pref prefix for output files
 */
public static void makeProf(String inDir, String pref){
  double[] p = null;
  int[][] seq = null;
  int N = 0;
  try{
    FileReader fr = new FileReader(pref+".pind.res");
```

```
  BufferedReader br = new BufferedReader(fr);
  while(br.readLine() != null){
    N++;
  }
  br.close();
  fr.close();
}
catch(Exception ex){
  ex.printStackTrace();
}
int lmax = 0;
p = new double[N];
seq = new int[N][];
try{
  FileReader fr = new FileReader(pref+".pind.res");
  BufferedReader br = new BufferedReader(fr);
  String line = null;
  int i = -1;
  while((line = br.readLine()) != null){
    i++;
    StringTokenizer st = new StringTokenizer(line, "\t");
    int n = st.countTokens()-1;
    if (n > lmax){
      lmax = n;
    }
    seq[i] = new int[n];
    Double D = new Double(st.nextToken());
    p[i] = D.doubleValue();
    int j = -1;
    while(st.hasMoreTokens()){
      j++;
      Integer I = new Integer(st.nextToken());
      seq[i][j] = I.intValue();
    }
  }
  br.close();
  fr.close();
}
catch(Exception ex){
  ex.printStackTrace();
}
int lmin = lmax;
int m = 2;
String[] lab = new String[m];
lab[0] = "U";
lab[1] = "S";
double[] lengthAtLeast = new double[lmax];
double[][] nreProf = new double[lmax][m];
for (int s = 0; s < N; s++){
  for (int i = 0; i < seq[s].length; i++){
    nreProf[i][seq[s][i]] += p[s];
    lengthAtLeast[i] += p[s];
  }
  if (seq[s].length < lmin){
    lmin = seq[s].length;
```

```
    }
  }
  for (int i = 0; i < lmax; i++){
    for (int j = 0; j < m; j++){
      nreProf[i][j] /= lengthAtLeast[i];
    }
  }
  double[][] reProf = new double[lmax][m];
  for (int s = 0; s < N; s++){
    for (int i = 0; i < seq[s].length; i++){
      reProf[i][seq[s][seq[s].length-1-i]] += p[s];
    }
  }
  for (int i = 0; i < lmax; i++){
    for (int j = 0; j < m; j++){
      reProf[i][j] /= lengthAtLeast[i];
    }
  }
  Vector<String> v = new Vector<String>();
  String sr = "nrePos";
  for (int i = 0; i < m; i++){
    sr += "\tpnre"+lab[i];
  }
  sr += "\trePos";
  for (int i = 0; i < m; i++){
    sr += "\tpre"+lab[i];
  }
  v.add(sr);
  for (int i = 0; i < lmax; i++){
    Integer nrepos = new Integer(i+1);
    Integer repos = new Integer(lmax-i);
    sr = nrepos.toString();
    for (int j = 0; j < m; j++){
      Double D = new Double(nreProf[i][j]);
      sr += "\t"+D.toString();
    }
    sr += "\t"+repos.toString();
    for (int j = 0; j < m; j++){
      Double D = new Double(reProf[i][j]);
      sr += "\t"+D.toString();
    }
    v.add(sr);
  }
  Utils.saveFile(v, pref+".prof.res");
  int[] len = new int[lmax-lmin+1];
  for (int i = 0; i < len.length; i++){
    len[i] = lmin+i;
  }
  double[] w = new double[len.length];
  for (int s = 0; s < N; s++){
    int l = seq[s].length-lmin;
    w[l] += p[s];
  }
  double[][] lenRho = new double[len.length][m];
  for (int s = 0; s < N; s++){
```

```
  for (int i = 1; i < seq[s].length; i++){
    int l = seq[s].length-lmin;
    lenRho[l][seq[s][i]] += p[s];
  }
}
for (int l = 0; l < len.length; l++){
  for (int j = 0; j < m; j++){
    lenRho[l][j] /= (double) (len[l]-1);
    lenRho[l][j] /= w[l];
  }
}
v = new Vector<String>();
sr = "l\tw\trhoU\trhoS";
v.add(sr);
for (int l = 0; l < w.length; l++){
  Integer L = new Integer(len[l]);
  sr = L.toString();
  Double D = new Double(w[l]);
  sr += "\t"+D.toString();
  D = new Double(lenRho[l][0]);
  sr += "\t"+D.toString();
  D = new Double(lenRho[l][1]);
  sr += "\t"+D.toString();
  v.add(sr);
}
Utils.saveFile(v, pref+".rhol.res");
BBSet bbs = new BBSet(inDir+"US.ab.txt");
double[][][] ptnre = new double[lmax][m][m];
for (int s = 0; s < N; s++){
  for (int i = 0; i < m; i++){
    ptnre[0][i][seq[s][0]] += p[s];
  }
}
for (int i = 0; i < m; i++){
  double sum = 0.;
  for (int j = 0; j < m; j++){
    sum += ptnre[0][i][j];
  }
  for (int j = 0; j < m; j++){
    ptnre[0][i][j] /= sum;
  }
}
for (int pos = 1; pos < lmax; pos++){
  for (int s = 0; s < N; s++){
    if (seq[s].length > pos){
      ptnre[pos][seq[s][pos-1]][seq[s][pos]] += p[s];
    }
  }
  for (int i = 0; i < m; i++){
    double sum = 0.;
    for (int j = 0; j < m; j++){
      sum += ptnre[pos][i][j];
    }
    for (int j = 0; j < m; j++){
      ptnre[pos][i][j] /= sum;
```

```
      }
    }
  }
  v = new Vector<String>();
  sr = "pos";
  for (int i = 0; i < m; i++){
    for (int j = 0; j < m; j++){
      sr += "\tp"+bbs.name[i]+bbs.name[j];
    }
  }
  v.add(sr);
  for (int pos = 0; pos < lmax; pos++){
    Integer I = new Integer(pos+1);
    sr = I.toString();
    for (int i = 0; i < m; i++){
      for (int j = 0; j < m; j++){
        Double D = new Double(ptnre[pos][i][j]);
        sr += "\t"+D.toString();
      }
    }
    v.add(sr);
  }
  Utils.saveFile(v, pref+".ptnre.res");
  double[][][] ptre = new double[lmax][m][m];
  for (int s = 0; s < N; s++){
    int pos = seq[s].length - lmax;
    if (pos >= 0){
      for (int i = 0; i < m; i++){
        ptre[0][i][seq[s][pos]] += p[s];
      }
    }
  }
  for (int i = 0; i < m; i++){
    double sum = 0.;
    for (int j = 0; j < m; j++){
      sum += ptre[0][i][j];
    }
    for (int j = 0; j < m; j++){
      ptre[0][i][j] /= sum;
    }
  }
  for (int pos = 1; pos < lmax; pos++){
    for (int s = 0; s < N; s++){
      int pos2 = seq[s].length-lmax+pos;
      if (pos2 >= 1){
        ptnre[pos][seq[s][pos2-1]][seq[s][pos2]] += p[s];
      }
    }
    for (int i = 0; i < m; i++){
      double sum = 0.;
      for (int j = 0; j < m; j++){
        sum += ptnre[pos][i][j];
      }
      for (int j = 0; j < m; j++){
        ptnre[pos][i][j] /= sum;
```

```
      }
    }
  }
  v = new Vector<String>();
  sr = "pos";
  for (int i = 0; i < m; i++){
    for (int j = 0; j < m; j++){
      sr += "\tp"+bbs.name[i]+bbs.name[j];
    }
  }
  v.add(sr);
  for (int pos = 0; pos < lmax; pos++){
    Integer I = new Integer(pos+1);
    sr = I.toString();
    for (int i = 0; i < m; i++){
      for (int j = 0; j < m; j++){
        Double D = new Double(ptnre[pos][i][j]);
        sr += "\t"+D.toString();
      }
    }
    v.add(sr);
  }
  Utils.saveFile(v, pref+".ptre.res");
}
/**
 * makes and saves individual species abundances for sigma = 3.5
 * @param inDir input directory (ends with "\\")
 * @param outDir output directory (ends with "\\")
 */
public static void makeSig35(String inDir, String outDir){
  new MaxEntModelW(outDir+"MEMW", 10, 20, 3.5, 16., inDir);
}
/**
 * makes and saves individual species abundances for sigma = 1.5
 * @param inDir input directory (ends with "\\")
 * @param outDir output directory (ends with "\\")
 */
public static void makeSig15(String inDir, String outDir){
  new MaxEntModelW(outDir+"MEMWSig1.5", 10, 20, 1.5, 16., inDir);
}
/**
 * saves distributions of chain lengths, composition as a function of chain length
 * and profiles (composition and transition probabilities)
 */
public static void glAndRholAndProfExamples(String inDir, String outDir){
  makeProf(inDir, outDir+"MEMW");
  makeProf(inDir, outDir+"MEMWSig1.5");
}
/**
 *
 * @param args
 */
public static void main(String[] args){

  String inDir = "input\\";
```

```
    String outDir = "output\\";
    makeSig35(inDir, outDir);
    makeSig15(inDir, outDir);
    glAndRholAndProfExamples(inDir, outDir);
  }
}
```

```
import java.util.Random;
import java.util.Vector;
/**
 * Geometric programming for solving a MaxEnt problem subject to
 * linear equality constraints (in the probability simplex)
 */
public class MaxEntOptim {
  /**
   * used for seeding multipliers
   */
  Random rand = null;
  /**
   * matrix in constraints Ax = b
   */
  double[][] A = null;
  /**
   * vector in constraints Ax = b
   */
  double[] b = null;
  /**
   * number of constraints
   */
  int m = 0;
  /**
   * number of variables in primal MaxEnt problem
   */
  int n = 0;
  /**
   * Lagrange multipliers, solution to the dual
   */
  double[] x = null;
  /**
   * Hessian of partition function Z
   */
  double[][] HZ = null;
  /**
   * Hessian of Legendre potential Gamma
   */
  double[][] HG = null;
  /**
   * gradient of partition function Z
   */
  double[] gradZ = null;
  /**
   * gradient of Legendre potential Gamma
   */
  double[] gradG = null;
  /**
   * partition function
   */
  double Z = 0.;
  /**
   * Legendre potential
```

```
 */
double gamma = 0.;
/**
 * descent direction
 */
double[] delta = null;
/**
 * solution to the primal
 */
double[] p = null;
/**
 * entropy
 */
double S = 0.;
/**
 * Geometric programming for solving a MaxEnt problem subject to linear
 * equality constraints (in the probability simplex)
 * @param A matrix in constraint Ap = b
 * @param b vector in constraint Ap = b
 */
public MaxEntOptim(double[][] A, double[] b){
  this.A = A;
  this.b = b;
  m = A.length;
  n = A[0].length;
  rand = new Random(2);
  x = new double[m];
  gradZ = new double[m];
  gradG = new double[m];
  HZ = new double[m][m];
  HG = new double[m][m];
  p = new double[n];
  for (int i = 0; i < m; i++){
    x[i] = 1.+rand.nextDouble();
  }
  double lambda = 1.;
  System.err.println("Potential\tNewtonStep\tEntropy\tInfeasibility");
  while(lambda > 1e-9){
    makeDerivatives();
    makeDelta();
    double alpha = getAlpha();
    for (int i = 0; i < m; i++){
      x[i] += alpha*delta[i];
    }
    lambda = getLambda();
    makeP();
    System.err.println(getGamma(x)+"\t"+lambda+"\t"+S+"\t"+getFeasibility());
  }
}
/**
 * checks feasibility of p
 * @return feasibility of p
 */
double getFeasibility(){
  double[] t = MatrixOp.multMatVec(A, p);
```

```
    double res = 0.;
    for (int i = 0; i < m; i++){
      res += Math.abs(t[i]-b[i]);
    }
    return res;
  }
  /**
   * computes primal solution and entropy
   */
  void makeP(){
    S = 0.;
    double z = getZ(x);
    for (int s = 0; s < n; s++){
      p[s] = Math.exp(-getDotProd(s,x))/z;
      S -= p[s]*Math.log(p[s]);
    }
  }
  /**
   * Newton step
   * @return Newton step
   */
  double getLambda(){
    double res = MatrixOp.geInnerProd(delta, gradG);
    return Math.abs(res);
  }
  /**
   * line search parameter (Armijo rule)
   * @return line search parameter (Armijo rule)
   */
  double getAlpha(){
    double s = 0.999;
    double beta = 0.5;
    double sg = 0.001;
    double alpha = s;
    double c = -MatrixOp.geInnerProd(gradG, gradG);
    double f = getGamma(x);
    double f2 = getGamma(x, alpha);
    int iter = 0;
    while(f-f2 < -sg*alpha*c && alpha > 1e-12){
      alpha *= beta;
      f2 = getGamma(x, alpha);
      iter++;
      if (iter > 1000){
        System.err.println("iter");
        iter = 0;
      }
    }
    return alpha;
  }
  /**
   * computes the descent direction
   */
  void makeDelta(){
    double[] t = new double[m];
    for (int i = 0; i < m; i++){
```

```
    t[i] = -gradG[i];
  }
  delta = MatrixOp.choleskySolve(HG, t);
}
/**
 * convenience method for computing ((As)^T)y
 * @param s column index
 * @param y vector of dim m
 * @return ((As)^T)y
 */
double getDotProd(int s, double[] y){
  double res = 0.;
  for (int i = 0; i < m; i++){
    res += A[i][s]*y[i];
  }
  return res;
}
/**
 * computes gradient and Hessian
 */
void makeDerivatives(){
  makeZ();
  makeGamma();
  makeGradZ();
  makeGradG();
  makeHZ();
  makeHG();
}
/**
 * Hessian of gamma
 */
void makeHG(){
  for (int i = 0; i < m; i++){
    for (int j = 0; j < m; j++){
      HG[i][j] = (HZ[i][j]-gradZ[i]*gradZ[j]/Z)/Z;
    }
  }
  double[][] L = MatrixOp.cholesky(HG);
  double min = Math.abs(L[0][0]);
  double max = Math.abs(L[0][0]);
  double smin = L[0][0];
  for (int i = 1; i < L.length; i++){
    double d = Math.abs(L[i][i]);
    if (d < min){
      min = d;
    }
    if (d > max){
      max = d;
    }
    if (L[i][i] < smin){
      smin = L[i][i];
    }
  }
  System.out.println(min+"\t"+smin);
  if (min < 0.0001){ // approximate detection of ill-conditioned problems
```

```
    for (int i = 0; i < m; i++){
      HG[i][i] += (min*min);
    }
  }
}
/**
 * Hessian of Z
 */
void makeHZ(){
  for (int i = 0; i < m; i++){
    for (int j = 0; j < m; j++){
      HZ[i][j] = 0.;
      for (int s = 0; s < n; s++){
        HZ[i][j] += A[i][s]*A[j][s]*Math.exp(-getDotProd(s,x));
      }
    }
  }
}
/**
 * partition function Z
 * @param y multipliers
 * @return partition function Z
 */
double getZ(double[] y){
  double res = 0.;
  for (int s = 0; s < n; s++){
    double t = getDotProd(s,y);
    res += Math.exp(-t);
  }
  return res;
}
/**
 * Legendre potential Gamma
 * @param y multipliers
 * @param alpha line search parameter
 * @return Legendre potential Gamma
 */
double getGamma(double[] y, double alpha){
  double[] t = new double[m];
  for (int i = 0; i < m; i++){
    t[i] = y[i]+alpha*delta[i];
  }
  return getGamma(t);
}
/**
 * Legendre potential Gamma
 * @param y multipliers
 * @return Legendre potential Gamma
 */
double getGamma(double[] y){
  double res = Math.log(getZ(y));
  for (int i = 0; i < m; i++){
    res += y[i]*b[i];
  }
  return res;
```

```
  }
  /**
   * partition function Z
   */
  void makeZ(){
    Z = getZ(x);
  }
  /**
   * Legendre potential Gamma
   */
  void makeGamma(){
    gamma = getGamma(x);
  }
  /**
   * gradient of Gamma
   */
  void makeGradG(){
    for (int i = 0; i < m; i++){
      gradG[i] = b[i]+gradZ[i]/Z;
    }
  }
  /**
   * gradient of Z
   */
  void makeGradZ(){
    for (int i = 0; i < m; i++){
      gradZ[i] = 0.;
      for (int s = 0; s < n; s++){
        double t = getDotProd(s,x);
        gradZ[i] -= A[i][s]*Math.exp(-t);
      }
    }
  }
  /**
   * default linear equality constraint = composition-1 + 2(digest-1)
   * @return default linear equality constraint = composition-1 + 2(digest-1)
   */
  public static LinEqCons getDefaultLEC(String inDir){
    Species sp = new Species(2, 16);
    BBSet bbs = new BBSet(inDir+"US.ab.txt");
    Vector<LinEqCons> v = new Vector<LinEqCons>();
    v.add(LinEqCons.removeLastRow(sp.getCompLEC(bbs.rho)));
    v.add(LinEqCons.removeLastRow(sp.getFragLEC(Species.loadFragAbund(inDir+"hepI.f.txt"),
        new CSpec(inDir+"US.hepI.txt", bbs), bbs)));
    v.add(LinEqCons.removeLastRow(sp.getFragLEC(Species.loadFragAbund(inDir+"hepIII.f.txt"),
        new CSpec(inDir+"US.hepIII.txt", bbs), bbs)));
    return new LinEqCons(v);
  }
}
```

```
import java.util.Random;
import java.util.Vector;
/**
 * species (enumeration of sequences) with specified chain length distribution;
 * the model of chain length distribution is obtained by projection
 */
public class MixSpecies {
  /**
   * number of disaccharides
   */
  int m = 2;
  /**
   * smallest chain length
   */
  int lmin = 0;
  /**
   * longest chain length
   */
  int lmax = 0;
  /**
   * number of chain lengths
   */
  int nc = 0;
  /**
   * chain lengths
   */
  int[] len = null;
  /**
   * chain length distribution
   */
  double[] w = null;
  /**
   * number of species
   */
  int N = 0;
  /**
   * sequences: seq[i] = sequence # i
   */
  int[][] seq = null;
  /**
   * based on this.w, lengthAtLeast[l] is the proportion of chains having
   * length at least l+1
   */
  double[] lengthAtLeast = null;
  /**
   * mixture model of BKHS chain lengths; sequence enumeration
   * @param lmin smallest chain length
   * @param lmax largest chain length
   * @param sigma spread of chain lengths
   * @param mu average chain length
   */
  public MixSpecies(int lmin, int lmax, double sigma, double mu){
    this.lmin = lmin;
```

```
    this.lmax = lmax;
    nc = lmax-lmin+1;
    w = getW(lmin, lmax, sigma, mu);
    lengthAtLeast = new double[lmax];
    for (int l = 0; l < lmax; l++){
      int j = l+1-lmin;
      if (j < 0){
        j = 0;
      }
      for (int i = j; i < w.length; i++){
        lengthAtLeast[l] += w[i];
      }
    }
    len = new int[nc];
    for (int i = 0; i < nc; i++){
      len[i] = lmin+i;
    }
    N = 0;
    for (int l = 0; l < nc; l++){
      N += (int) Math.pow((double) m, (double) len[l]);
    }
    seq = new int[N][];
    int s = -1;
    for (int l = 0; l < nc; l++){
      int max = (int) Math.pow((double) m, (double) len[l]);
      for (int i = 0; i < max; i++){
        s++;
        seq[s] = new int[len[l]];
      }
    }
    s = -1;
    for (int l = 0; l < nc; l++){
      Species sp = new Species(m, len[l]);
      for (int i = 0; i < sp.N; i++){
        s++;
        for (int j = 0; j < sp.n; j++){
          seq[s][j] = sp.seq[i][j];
        }
      }
    }
    MatrixOp.printVec(w);
  }
  /**
   * combines constraints on vector of species abundances: normalization, composition
   * and fragment length distributions (fragFile[]) after digestion (csFile[])
   * @param bbs disaccharide set, with composition in bbs.rho
   * @param csFile files containing cleavage specificities
   * @param fragFile files containing distributions of fragment lengths
   * @return combined constraints on vector of species abundances: normalization,
   * composition and fragment length distributions (fragFile[]) after digestion (csFile[])
   */
  LinEqCons getCompleteLEC(BBSet bbs, String[] csFile, String[] fragFile){
    Vector<LinEqCons> v = new Vector<LinEqCons>();
    v.add(getNormLEC());
    v.add(LinEqCons.removeLastRow(getRhoLEC(bbs)));
```

```
  for (int i = 0; i < csFile.length; i++){
    CSpec cs = new CSpec(csFile[i], bbs);
    double[] f = Species.loadFragAbund(fragFile[i]);
    v.add(LinEqCons.removeLastRow(getFragLEC(f, cs, bbs)));
  }
  v.add(LinEqCons.removeLastRow(getWLEC()));
  return new LinEqCons(v);
}
/**
 * constraint for homogeneity of composition with chain length combined to
 * digest constraints
 * @param bbs disaccharide set
 * @param csFile files containing cleavage specificities
 * @param fragFile files containing distributions of fragment lengths
 * @return constraint for homogeneity of composition with chain length
 * combined to digest constraints
 */
LinEqCons getRhoLHomFeasLEC(BBSet bbs, String[] csFile, String[] fragFile){
  Vector<LinEqCons> v = new Vector<LinEqCons>();
  v.add(getNormLEC());
  for (int i = 0; i < csFile.length; i++){
    CSpec cs = new CSpec(csFile[i], bbs);
    double[] f = Species.loadFragAbund(fragFile[i]);
    v.add(LinEqCons.removeLastRow(getFragLEC(f, cs, bbs)));
  }
  v.add(LinEqCons.removeLastRow(getWLEC()));
  v.add(getRhoLHomLEC(bbs));
  return new LinEqCons(v);
}
/**
 * constraint for homogeneity of composition with chain length
 * @param bbs disaccharide set
 * @return constraint for homogeneity of composition with chain length
 */
LinEqCons getRhoLHomLEC(BBSet bbs){
  double[][] A = new double[nc][N];
  double[] b = new double[nc];
  String[] tp = new String[nc];
  for (int i = 0; i < nc; i++){
    b[i] = bbs.rho[1]*w[i];
    tp[i] = new String("rhol");
    for (int s = 0; s < N; s++){
      if (seq[s].length == len[i]){
        for (int k = 1; k < seq[s].length; k++){
          if (seq[s][k] == 1){
            A[i][s] += 1.;
          }
        }
        A[i][s] /= (double) (len[i]-1);
      }
    }
  }
  return new LinEqCons(A, b, tp);
}
/**
```

```
 * linear equality constraint on species abundances for chain length distribution
 * @return linear equality constraint on species abundances for
 * chain length distribution
 */
LinEqCons getWLEC(){
  double[][] A = new double[nc][N];
  double[] b = new double[nc];
  String[] tp = new String[nc];
  for (int i = 0; i < nc; i++){
    b[i] = w[i];
    tp[i] = new String("W");
    for (int s = 0; s < N; s++){
      if (seq[s].length == len[i]){
        A[i][s] = 1.;
      }
    }
  }
  return new LinEqCons(A, b, tp);
}
/**
 * sum-to-1 constraint on vector of species abundances
 * @return sum-to-1 constraint on vector of species abundances
 */
LinEqCons getNormLEC(){
  double[][] A = new double[1][N];
  double[] b = new double[1];
  String[] lab = new String[1];
  b[0] = 1.;
  lab[0] = new String("norm");
  for (int i = 0; i < N; i++){
    A[0][i] = 1.;
  }
  return new LinEqCons(A, b, lab);
}
/**
 * linear equality constraint on the vector of species abundances for distribution
 * f of fragment length after digestion by enzyme (cleavage specificities in cs.c)
 * @param f distribution of fragment length
 * @param cs cleavage specificities
 * @param bbs disaccharide set (composition in bbs.rho)
 * @return linear equality constraint on the vector of species abundances for
 * distribution f of fragment length after digestion by enzyme
 * (cleavage specificities in cs.c)
 */
LinEqCons getFragLEC(double[] f, CSpec cs, BBSet bbs){
  double[][] A = new double[f.length][N];
  double[] b = new double[f.length];
  String[] lab = new String[f.length];

  for (int i = 0; i < b.length; i++){
    b[i] = f[i];
    lab[i] = new String("frag");
  }
  int lm = f.length;
  for (int l = 0; l < lm-1; l++){
```

```
    for (int s = 0; s < N; s++){
      A[l][s] = getFragContrib(s, l+1, cs);
    }
  }
  for (int s = 0; s < N; s++){
    A[lm-1][s] = 0.;
    for (int l = lm-1; l < seq[s].length-1; l++){
      A[lm-1][s] += getFragContrib(s, l+1, cs);
    }
  }
  double denom = 0.;
  for (int i = 0; i < bbs.m; i++){
    denom += bbs.rho[i]*cs.c[i];
  }
  double avL = 0.;
  for (int l = 0; l < nc; l++){
    avL += w[l]*(double)len[l];
  }
  denom *= (avL-1.);
  for (int i = 0; i < A.length; i++){
    for (int j = 0; j < A[i].length; j++){
      A[i][j] /= denom;
    }
  }
  return new LinEqCons(A, b, lab);
}
/**
 * contribution of species s to expected number of fragments of length
 * ll after digestion by enzyme with cleavage specificities given by cs.c
 * @param s species index
 * @param ll fragment length
 * @param cs cleavage specificities
 * @return contribution of species s to expected number of fragments of length
 * ll after digestion by enzyme with cleavage specificities given by cs.c
 */
double getFragContrib(int s, int ll, CSpec cs){
  int n = seq[s].length;
  double res = 0.;
  if (ll > n){
    return res;
  }
  for (int i = 1; i < n-ll; i++){
    double t = cs.c[seq[s][i]];
    for (int j = 1; j <= ll-1; j++){
      t *= (1.-cs.c[seq[s][i+j]]);
    }
    t *= cs.c[seq[s][i+ll]];
    res += t;
  }
  double t = cs.c[seq[s][n-ll]];
  for (int j = n-ll+1; j < n; j++){
    t *= (1.-cs.c[seq[s][j]]);
  }
  res += t;
  return res;
```

```
}
/**
 * linear equality constraint on species abundances for overall disaccharide
 * composition between positions 2 and n
 * @param bbs set of disaccharides with composition in bbs.rho
 * @return linear equality constraint on species abundances for overall
 * disaccharide composition between positions 2 and n
 */
LinEqCons getRhoLEC(BBSet bbs){
  double[][] A = new double[m][N];
  double[] b = new double[m];
  String[] tp = new String[m];
  double avL = 0.;
  for (int l = 0; l < nc; l++){
    avL += w[l]*(double) len[l];
  }
  for (int j = 0; j < m; j++){
    b[j] = bbs.rho[j];
    tp[j] = new String("rho");
    for (int s = 0; s < N; s++){
      for (int k = 1; k < seq[s].length; k++){
        if (seq[s][k] == j){
          A[j][s] += 1.;
        }
      }
      A[j][s] /= (avL-1.);
    }
  }

  return new LinEqCons(A, b, tp);
}
/**
 * testing that homogeneity of composition across chain lengths is
 * feasible after incorporating digest constraints;
 * Note: requires large memory (-Xmx8000M)
 * @param inDir input directory (ends with "\\")
 */
public static void testRhoLHomFeas(String inDir){
  int lmin = 10;
  int lmax = 20;
  double sigma = 3.5;
  double mu = 16.;
  BBSet bbs = new BBSet(inDir+"US.ab.txt");
  MixSpecies msp = new MixSpecies(lmin, lmax, sigma, mu);
  String[] csFile = new String[2];
  String[] fragFile = new String[2];
  csFile[0] = new String(inDir+"US.hepI.txt");
  csFile[1] = new String(inDir+"US.hepIII.txt");
  fragFile[0] = new String(inDir+"hepI.f.txt");
  fragFile[1] = new String(inDir+"hepIII.f.txt");
  LinEqCons lec = msp.getRhoLHomFeasLEC(bbs, csFile, fragFile);
  SimplexPhaseI sp1 = new SimplexPhaseI(lec.A, lec.b);
  System.err.println(sp1.finalCost);
}
/**
```

```
 * computes chain length distribution. First a Gaussian (mu, sigma) restricted
 * between lmin and lmax is utilized as approximation. Then, projection on the
 * probability simplex with constraint to preserve mu is performed.
 * @param lmin smallest chain length
 * @param lmax longest chain length
 * @param sigma standard deviation of Gaussian approximation
 * @param mu desired average chain length
 * @return chain length distribution: between lmin and lmax with mean mu
 */
public static double[] getW(int lmin, int lmax, double sigma, double mu){
  int nc = lmax-lmin+1;
  double[] res = new double[nc];
  double[] t = new double[nc];
  for (int l = lmin; l <= lmax; l++){
    int i = l-lmin;
    t[i] = 0.;
    double dx = 0.001;
    double x = (double) l;
    while(x < (double) (l+1)){
      x += dx;
      double fx = Math.exp(-0.5*(x-mu)*(x-mu)/(sigma*sigma))
          /(sigma*Math.sqrt(2.*Math.PI));
      t[i] += fx*dx;
    }
  }
  double sum = 0.;
  for (int i = 0; i < nc; i++){
    sum += t[i];
  }
  for (int i = 0; i < nc; i++){
    res[i] = t[i]/sum;
  }
  double[][] A = new double[nc+4][nc];
  double[] b = new double[nc+4];
  int r = -1;
  for (int i = 0; i < nc; i++){
    b[i] = 0.;
    r++;
    A[r][i] = -1.;
  }
  r++;
  b[r] = 1.;
  for (int i = 0; i < nc; i++){
    A[r][i] = 1.;
  }
  r++;
  b[r] = -1.;
  for (int i = 0; i < nc; i++){
    A[r][i] = -1.;
  }
  r++;
  b[r] = mu;
  for (int i = 0; i < nc; i++){
    A[r][i] = (double)(lmin+i);
  }
```

```
    r++;
    b[r] = -mu;
    for (int i = 0; i < nc; i++){
      A[r][i] = -(double)(lmin+i);
    }
    ProjOnPolyHSet proj = new ProjOnPolyHSet(res, A, b, new Random());
    sum = 0.;
    for (int i = 0; i < res.length; i++){
      res[i] = proj.optimum[i];
      if (res[i] < 0.){
        res[i] = 0.;
      }
      sum += res[i];
    }
    double av = 0.;
    for (int i = 0; i < res.length; i++){
      res[i] /= sum;
      av += res[i]*(double)(lmin+i);
    }
    System.err.println(av);
    return res;
  }
  /**
   *
   * @param args
   */
  public static void main(String[] args){
    String inDir = "input\\";
    testRhoLHomFeas(inDir);
  }
}
```

```
import java.util.Random;
import java.util.Vector;
/**
 * represents a nonhomogeneity and independence (N&I) model:
 * different building block composition at each position in a chain
 * and independence between positions; field this.g contains the expected
 * heparinase fragment length distribution under model N&I
 */
public class NIModel {
  /**
   * length of a chain
   */
  int n = 0;
  /**
   * number of building block types
   */
  int m = 0;
  /**
   * gamma[i][j] is the proportion of building block j at position i from
   * the non-reducing end
   */
  double[][] gamma = null;
  /**
   * cumulative version of gamma: gamF[i][j] = sum_{k=0}^j gamma[i][k]
   */
  double[][] gamF = null;
  /**
   * overall cleavage probability
   */
  double c = 0;
  /**
   * see equation of g(l) in paper
   */
  double alpha = 0.;
  /**
   * cleavage probability at position i
   */
  double[] ci = null;
  /**
   * set of disaccharides and their overall proportions
   */
  BBSet bbs = null;
  /**
   * cleavage specificities/yields for one heparinase
   */
  CSpec cs = null;
  /**
   * expected distribution of fragment length in on heparinase digest
   */
  double[] g = null;
  /**
   * same as this.g but cumulative for length ll >= lm
   */
```

```
double[] h = null;
/**
 * maximum measured fragment length
 */
int lm = 0;
/**
 * represents a nonhomogeneity and independence (N&I) model:
 * different building block composition at each position in a chain
 * and independence between positions
 * @param bbs disaccharides and their overall proportions
 * @param cs cleavage specificities/yields for one heparinase
 * @param gam initial (random or optimized) matrix Gamma of disaccharide
 * proportions along chains;
 * this initial matrix is then projected to satisfy constraints
 * @param lm maximum measured fragment length
 */
public NIModel(BBSet bbs, CSpec cs, double[][] gam, int lm){
  this.lm = lm;
  this.bbs = bbs;
  this.cs = cs;
  m = bbs.m;
  n = gam.length;
  gamma = new double[n][m];
  for (int i = 0; i < n; i++){
    for (int j = 0; j < m; j++){
      gamma[i][j] = gam[i][j];
      // projection is not always perfect due to numerical accuracy
      if (gamma[i][j] > 1.){
        gamma[i][j] = 1.;
      }
      if (gamma[i][j] < 0.){
        gamma[i][j] = 0.;
      }
    }
  }
  gamF = new double[n][m];
  for (int i = 0; i < n; i++){
    gamF[i][0] = gamma[i][0];
    for (int j = 1; j < m; j++){
      gamF[i][j] = gamF[i][j-1]+gamma[i][j];
    }
  }
  makeC();
  makeCi();
  makeG();
  makeH();
}
/**
 * this.h: fragment length distribution expected under N&I
 * and cumulative for length ll >= lm
 */
void makeH(){
  h = new double[lm];
  int max = lm;
  if (max > n-1){
```

```
    max = n-1;
  }
  for (int i = 0; i < max; i++){
    h[i] = g[i];
  }
  for (int i = lm; i < n; i++){
    h[lm-1] += g[i];
  }
}
/**
 * generates one random BKHS sequence based on this.gamma
 * @param rand
 * @return one random BKHS sequence based on this.gamma
 */
int[] getSequence(Random rand){
  int[] res = new int[n];
  for (int i = 0; i < n; i++){
    res[i] = Utils.getRandIndexInF(gamF[i], rand);
  }
  return res;
}
/**
 * generate a random set of cleavage positions in seq based on
 * heparinase cleavage specificities/yields
 * @param seq BKHS sequence
 * @param rand
 * @return a random set of cleavage positions in seq based on
 * heparinase cleavage specificities/yields
 */
int[] getCleavages(int[] seq, Random rand){
  Vector<Integer> cuts = new Vector<Integer>();
  for (int i = 1; i < n; i++){
    double d = rand.nextDouble();
    if (d < cs.c[seq[i]]){
      cuts.add(new Integer(i));
    }
  }
  cuts.add(new Integer(n));
  int[] res = new int[cuts.size()];
  for (int i = 0; i < cuts.size(); i++){
    Integer I = cuts.elementAt(i);
    res[i] = I.intValue();
  }
  return res;
}
/**
 * generates one BKHS sequence and cleaves;
 * see getFragments() of CleavedSequence to obtain fragments
 * @param rand
 * @return one cleaved BKHS chain
 */
CleavedSequence getCleavageSequence(Random rand){
  int[] seq = null;
  int[] cuts = new int[1];
  while(cuts.length < 2){
```

```
    seq = getSequence(rand);
    cuts = getCleavages(seq, rand);
  }
  return new CleavedSequence(seq, cuts);
}
/**
 * distribution of fragment length after heparinase digestion
 * and expected under N&I
 */
void makeG(){
  g = new double[n];
  double[][] omega = new double[n][n];
  for (int i = 0; i < n; i++){
    omega[i][i] = 1.-ci[i];
    for (int j = i+1; j < n; j++){
      omega[i][j] = omega[i][j-1]*(1.-ci[j]);
    }
  }
  alpha = 0.;
  for (int i = 1; i < n; i++){
    alpha += ci[i];
  }
  // ll = 1
  g[0] = ci[n-1];
  for (int i = 1; i < n-1; i++){
    g[0] += ci[i]*ci[i+1];
  }
  g[0] /= alpha;
  // 2 <= ll <= n-2
  for (int ll = 2; ll <= n-2; ll++){
    int l = ll-1;
    g[l] = ci[n-ll]*omega[n-ll+1][n-1];
    for (int i = 1; i < n-ll; i++){
      g[l] += ci[i]*ci[i+ll]*omega[i+1][i+ll-1];
    }
    g[l] /= alpha;
  }
  // ll = n-1
  g[n-2] = ci[1]*omega[2][n-1];
  g[n-2] /= alpha;
}
/**
 * this.ci: cleavage probabilities at each position i
 * (average quantity in the mixture, defined by this.gamma and this.cs)
 */
void makeCi(){
  ci = new double[n];
  for (int i = 1; i < n; i++){
    ci[i] = 0.;
    for (int j = 0; j < m; j++){
      ci[i] += gamma[i][j]*cs.c[j];
    }
  }
}
/**
```

```
 * overall cleavage probability c
 */
void makeC(){
  c = 0.;
  for (int i = 0; i < m; i++){
    c += bbs.rho[i]*cs.c[i];
  }
}
/**
 * numerical check for this.g
 * @param rand
 */
void checkGL(Random rand){
  int nsim = 10000000;
  int totfrag = 0;
  double[] f = new double[n];
  for (int sim = 0; sim < nsim; sim++){
    CleavedSequence cseq = getCleavageSequence(rand);
    int[][] frag = cseq.getFragments();
    totfrag += frag.length;
    for (int i = 0; i < frag.length; i++){
      f[frag[i].length-1] += 1.;
    }
  }
  for (int i = 0; i < n; i++){
    double d = 0.;
    if (i < lm){
      d = h[i];
    }
    System.out.println((i+1)+"\t"+g[i]+"\t"+
    (f[i]/(double) totfrag)+"\t"+f[i]+"\t"+d);
  }
}
/**
 * turns a gamma matrix (n by m) in vector form
 * @param G gamma matrix (n by m)
 * @return a vector representing matrix G
 */
public static double[] toVector(double[][] G){
  double[] res = new double[G.length*G[0].length];
  int k = -1;
  for (int j = 0; j < G[0].length; j++){
    for (int i = 0; i < G.length; i++){
      k++;
      res[k] = G[i][j];
    }
  }
  return res;
}
/**
 * turns a vector into a gamma matrix
 * @param g vector of dimension (m*nn)
 * @param nn BKHS chain length
 * @return a matrix representing a gamma vector
 */
```

```
  public static double[][] toMatrix(double[] g, int nn){
    int mm = g.length/nn;
    double[][] res = new double[nn][mm];
    int k = -1;
    for (int i = 0; i < mm; i++){
      for (int j = 0; j < nn; j++){
        k++;
        res[j][i] = g[k];
      }
    }
    return res;
  }
}
```

```java
import java.util.Random;
/**
 * linear inequality constraints on a N&I model: nonnegativity, sum to 1 at each position
 * and overall disaccharide abundances
 */
public class NIModelLIC {
  /**
   * matrix in Ax <= b
   */
  double[][] A = null;
  /**
   * vector in Ax <= b
   */
  double[] b = null;
  /**
   * chain length
   */
  int n = 0;
  /**
   * number of building blocks
   */
  int m = 0;
  /**
   * linear inequality constraints on a N&I model:
   * Ax <= b including nonnegativity, sum to 1 at each position and
   * overall disaccharide abundances
   * @param n chain length in PModel
   * @param bbs set of disaccharides
   * @param pos positions with composition fixed by zeta
   * @param zeta composition at positions in pos
   */
  public NIModelLIC(int n, BBSet bbs, double[][] zeta, int[] pos){
    this.n = n;
    m = bbs.m;
    A = new double[m*n+2*m+2*n+2*(pos.length*(m-1))][n*m];
    b = new double[m*n+2*m+2*n+2*(pos.length*(m-1))];

    for (int rr = 1; rr <= n*m; rr++){
      int r = rr-1;
      b[r] = 0.;
      A[r][r] = -1.;
    }
    for (int rr = n*m+1; rr <= n*m+m; rr++){
      int r = rr-1;
      b[r] = bbs.rho[r-n*m]*(double) (n-1);
      for (int kk = 1; kk <= n*m; kk++){
        int k = kk-1;
        if (getJJ(kk) == rr-n*m && getII(kk) != 1){
          A[r][k] = 1.;
        }
      }
    }
    for (int rr = n*m+m+1; rr <= n*m+2*m; rr++){
```

```
    int r = rr-1;
    b[r] = -bbs.rho[r-n*m-m]*(double) (n-1);
    for (int kk = 1; kk <= n*m; kk++){
      int k = kk-1;
      if (getJJ(kk) == rr-n*m-m && getII(kk) != 1){
        A[r][k] = -1.;
      }
    }
  }
  for (int rr = n*m+2*m+1; rr <= n*m+2*m+n; rr++){
    int r = rr-1;
    b[r] = 1.;
    for (int kk = 1; kk <= n*m; kk++){
      int k = kk-1;
      if (getII(kk) == rr-n*m-2*m){
        A[r][k] = 1.;
      }
    }
  }
  for (int rr = n*m+2*m+n+1; rr <= n*m+2*m+2*n; rr++){
    int r = rr-1;
    b[r] = -1.;
    for (int kk = 1; kk <= n*m; kk++){
      int k = kk-1;
      if (getII(kk) == rr-n*m-2*m-n){
        A[r][k] = -1.;
      }
    }
  }
  int rr = n*m+2*m+2*n;
  for (int p = 0; p < pos.length; p++){
    int pp = pos[p]+1;
    for (int z = 0; z < m-1; z++){
      int zz = z+1;
      rr++;
      int r = rr-1;
      b[r] = zeta[p][z];
      for (int kk = 1; kk <= n*m; kk++){
        int k = kk-1;
        if (getII(kk) == pp && getJJ(kk) == zz){
          A[r][k] = 1.;
        }
      }
      rr++;
      r = rr-1;
      b[r] = -zeta[p][z];
      for (int kk = 1; kk <= n*m; kk++){
        int k = kk-1;
        if (getII(kk) == pp && getJJ(kk) == zz){
          A[r][k] = -1.;
        }
      }
    }
  }
}
```

```
/**
 * linear inequality constraints on a N&I model:
 * Ax <= b including nonnegativity, sum to 1 at each position and overall
 * disaccharide abundances
 * @param n chain length in PModel
 * @param bbs set of building blocks (disaccharides)
 */
public NIModelLIC(int n, BBSet bbs){
  this.n = n;
  m = bbs.m;
  A = new double[m*n+2*m+2*n][n*m];
  b = new double[m*n+2*m+2*n];
  for (int rr = 1; rr <= n*m; rr++){
    int r = rr-1;
    b[r] = 0.;
    A[r][r] = -1.;
  }
  for (int rr = n*m+1; rr <= n*m+m; rr++){
    int r = rr-1;
    b[r] = bbs.rho[r-n*m]*(double) (n-1);
    for (int kk = 1; kk <= n*m; kk++){
      int k = kk-1;
      if (getJJ(kk) == rr-n*m && getII(kk) != 1){
        A[r][k] = 1.;
      }
    }
  }
  for (int rr = n*m+m+1; rr <= n*m+2*m; rr++){
    int r = rr-1;
    b[r] = -bbs.rho[r-n*m-m]*(double) (n-1);
    for (int kk = 1; kk <= n*m; kk++){
      int k = kk-1;
      if (getJJ(kk) == rr-n*m-m && getII(kk) != 1){
        A[r][k] = -1.;
      }
    }
  }
  for (int rr = n*m+2*m+1; rr <= n*m+2*m+n; rr++){
    int r = rr-1;
    b[r] = 1.;
    for (int kk = 1; kk <= n*m; kk++){
      int k = kk-1;
      if (getII(kk) == rr-n*m-2*m){
        A[r][k] = 1.;
      }
    }
  }
  for (int rr = n*m+2*m+n+1; rr <= n*m+2*m+2*n; rr++){
    int r = rr-1;
    b[r] = -1.;
    for (int kk = 1; kk <= n*m; kk++){
      int k = kk-1;
      if (getII(kk) == rr-n*m-2*m-n){
        A[r][k] = -1.;
      }
```

```
    }
  }
}
/**
 * index kk (1 to n*m) in vector gamma corresponding to entry (ii,jj) in matrix Gamma
 * @param ii row index in matrix Gamma (1 to n)
 * @param jj column index in matrix Gamma (1 to m)
 * @return index kk (1 to n*m) in vector gamma corresponding to entry
 * (ii,jj) in matrix Gamma
 */
int getKK(int ii, int jj){
  int res = ii+(jj-1)*n;
  return res;
}
/**
 * column index jj (1 to m) in matrix Gamma corresponding to index kk
 * (1 to n*m) in vector gamma
 * @param kk index (1 to n*m) in vector gamma
 * @return column index jj (1 to m) in matrix Gamma corresponding to
 * index kk (1 to n*m) in vector gamma
 */
int getJJ(int kk){
  int res = 1+(kk-1)/n;
  return res;
}
/**
 * row index ii (1 to n) in matrix Gamma corresponding to index kk
 * (1 to n*m) in vector gamma
 * @param kk index (1 to n*m) in vector gamma
 * @return row index ii (1 to n) in matrix Gamma corresponding to
 * index kk (1 to n*m) in vector gamma
 */
int getII(int kk){
  int res = kk-(getJJ(kk)-1)*n;
  return res;
}
/**
 * for testing
 * @param args
 */
public static void main(String[] args){
  String inDir = "input\\";
  String file = inDir+"US.ab.txt";
  BBSet bbs = new BBSet(file);
  int n = 16;
  Random rand = new Random();
  int m = bbs.m;
  int[] pos = new int[1];
  pos[0] = n-1;
  double[][] zeta = new double[pos.length][m];
  zeta[0][0] = 0.95;
  NIModelLIC plic = new NIModelLIC(n , bbs, zeta, pos);
  for (int sim = 0; sim < 1; sim++){
    double[][] gam = new double[n][m];
    for (int i = 0; i < n; i++){
```

```
        for (int j = 0; j < m; j++){
          gam[i][j] = rand.nextDouble();
        }
      }
      ProjOnPolyHSet proj = new ProjOnPolyHSet(NIModel.toVector(gam),
          plic.A, plic.b, rand);
      gam = NIModel.toMatrix(proj.optimum, n);
      MatrixOp.printMat(gam);
    }
    MatrixOp.printVec(bbs.rho);
    System.out.println("Note: projection is not perfect, "
        + "yielding sometimes entries < 0 or > 1."
        + " This is accounted for (corrected) in class NIModelSA");
  }
}
```

```
import java.io.BufferedReader;
import java.io.BufferedWriter;
import java.io.FileReader;
import java.io.FileWriter;
import java.util.Random;
import java.util.StringTokenizer;
import java.util.Vector;
/**
 * Fitting of N&I model parameters with heparinase digest constraints via simulated annealing
 */
public class NIModelSA {
  /**
   * position models (e.g. one for hepI and one for hepIII)
   */
  NIModel[] pm = null;
  /**
   * f[i][] is the distribution of fragment length (experimental data) for digest model i
   */
  double[][] f = null;
  /**
   * for perturbations
   */
  Random rand = null;
  /**
   * chain length
   */
  int n = 0;
  /**
   * number of building blocks
   */
  int m = 0;
  /**
   * number of digests
   */
  int nd = 0;
  /**
   * enzyme specificities/yields
   */
  CSpec[] cs = null;
  /**
   * set of building blocks
   */
  BBSet bbs = null;
  /**
   * position model parameters
   */
  double[][] gamma = null;
  /**
   * best encountered model
   */
  double[][] gamBest = null;
  /**
   * buffer for this.gamma
```

```
 */
double[][] gamBuff = null;
/**
 * linear inequality constraints provided by overall building block proportions,
 * sum to 1 at each position and nonnegativity
 */
NIModelLIC plic = null;
/**
 * number of perturbations at each temperature
 */
int nPert = 400;
/**
 * annealing schedule (geometric temperature decrease)
 */
double alpha = 0.999;
/**
 * best encountered value of the objective function
 */
double bestE = 0.;
/**
 * Fitting of N&I model parameters with heparinase digest constraints via simulated annealing;
 * special constructor which adds constraints
 * @param n BKHS chain length
 * @param bbs disachharides and their overall proportions
 * @param specFile cleavage specificities/yields
 * @param consFile constraints (experimental fragment length distributions)
 * @param outFile output file for modeled fragment length distributions
 * @param modFile output file for optimized matrix Gamma of N&I model
 * @param zeta specified compositions at positions in pos (additional constraints)
 * @param pos positions at which composition is further specified (additional constraints)
 * @param rand
 */
public NIModelSA(int n, BBSet bbs, String[] specFile, String[] consFile, String[] outFile,
    String modFile, double[][] zeta, int[] pos, Random rand){
  this.bbs = bbs;
  m = bbs.m;
  this.n = n;
  this.rand = rand;
  nd = specFile.length;
  cs = new CSpec[nd];
  for (int i = 0; i < nd; i++){
    cs[i] = new CSpec(specFile[i], bbs);
  }
  loadConstraints(consFile);
  initModel(zeta, pos);
  double t = initT();
  double t0 = t;
  int nup = 1;
  double E = bestE;
  while(nup > 0){
    t *= alpha;
    nup = 0;
    for (int i = 0; i < nPert; i++){
      perturb();
      double E2 = getE();
```

```
      boolean accept = false;
      if (E2 < E){
        accept = true;
      }
      else{
        double p = Math.exp(-Math.abs(E2-E)/t);
        if (rand.nextDouble() < p){
          accept = true;
          nup++;
        }
      }
      if (accept){
        E = E2;
        if (E < bestE){
          bestE = E;
          saveGamma();
        }
      }
      else{
        restore();
      }
    }
    if (t < t0/100000.){
      break;
    }
  }
  for (int i = 0; i < n; i++){
    for (int j = 0; j < m; j++){
      gamma[i][j] = gamBest[i][j];
    }
  }
  pm = new NIModel[nd];
  for (int i = 0; i < nd; i++){
    pm[i] = new NIModel(bbs, cs[i], gamma, f[i].length);
    saveFit(outFile[i], i);
  }
  savePM(modFile);
}
/**
 * initialization when additional constraints
 * (specified compositions zeta in positions pos) are given
 * @param zeta disaccharide composition
 * @param pos position in chains
 */
void initModel(double[][] zeta, int[] pos){
  gamma = new double[n][m];
  gamBuff = new double[n][m];
  plic = new NIModelLIC(n, bbs, zeta, pos);
  for (int i = 0; i < n; i++){
    for (int j = 0; j < m; j++){
      gamma[i][j] = rand.nextDouble();
    }
  }
  ProjOnPolyHSet proj = new ProjOnPolyHSet(NIModel.toVector(gamma), plic.A, plic.b, rand);
  gamma = NIModel.toMatrix(proj.optimum, n);
```

```
    pm = new NIModel[nd];
    for (int i = 0; i < nd; i++){
      pm[i] = new NIModel(bbs, cs[i], gamma, f[i].length);
    }
    for (int i = 0; i < n; i++){
      for (int j = 0; j < m; j++){
        gamBuff[i][j] = gamma[i][j];
      }
    }
  }
  /**
   * Fitting of parameters of a N&I model with heparinase digest constraints
   * via simulated annealing
   * @param n BKHS chain length
   * @param bbs disachharides and their overall proportions
   * @param specFile cleavage specificities/yields
   * @param consFile constraints (experimental fragment length distributions)
   * @param outFile output file for modeled fragment length distributions
   * @param modFile output file for optimized matrix Gamma of N&I model
   * @param rand
   */
  public NIModelSA(int n, BBSet bbs, String[] specFile, String[] consFile,
      String[] outFile, String modFile, Random rand){
    this.bbs = bbs;
    m = bbs.m;
    this.n = n;
    this.rand = rand;
    nd = specFile.length;
    cs = new CSpec[nd];
    for (int i = 0; i < nd; i++){
      cs[i] = new CSpec(specFile[i], bbs);
    }
    loadConstraints(consFile);
    initModel();
    double t = initT();
    int nup = 1;
    double E = bestE;
    while(nup > 0){
      t *= alpha;
      nup = 0;
      for (int i = 0; i < nPert; i++){
        perturb();
        double E2 = getE();
        boolean accept = false;
        if (E2 < E){
          accept = true;
        }
        else{
          double p = Math.exp(-Math.abs(E2-E)/t);
          if (rand.nextDouble() < p){
            accept = true;
            nup++;
          }
        }
        if (accept){
```

```
        E = E2;
        if (E < bestE){
          bestE = E;
          saveGamma();
        }
      }
      else{
        restore();
      }
    }
  }
  for (int i = 0; i < n; i++){
    for (int j = 0; j < m; j++){
      gamma[i][j] = gamBest[i][j];
    }
  }
  pm = new NIModel[nd];
  for (int i = 0; i < nd; i++){
    pm[i] = new NIModel(bbs, cs[i], gamma, f[i].length);
    saveFit(outFile[i], i);
  }
  savePM(modFile);
}
/**
 * saves matrix Gamma of the optimized N&I model
 * @param file
 */
void savePM(String file){
  try{
    FileWriter fw = new FileWriter(file);
    BufferedWriter bw = new BufferedWriter(fw);
    bw.write("position");
    for (int j = 0; j < m; j++){
      bw.write("\t"+bbs.name[j]);
    }
    bw.newLine();
    for (int i = 0; i < n; i++){
      Integer I = new Integer(i+1);
      bw.write(I.toString());
      for (int j = 0; j < m; j++){
        Double D = new Double(pm[0].gamma[i][j]);
        bw.write("\t"+D.toString());
      }
      bw.newLine();
    }
    bw.close();
    fw.close();
  }
  catch(Exception ex){
    ex.printStackTrace();
  }
}
/**
 * saves experimental and modeled (optimized) distribution of fragment length
 * after digestion by heparinase i
```

```
 * @param file output file
 * @param i heparinase number
 */
void saveFit(String file, int i){
  try{
    FileWriter fw = new FileWriter(file);
    BufferedWriter bw = new BufferedWriter(fw);
    bw.write("l\tfexp\thmod");
    bw.newLine();
    for (int l = 0; l < pm[i].lm; l++){
      Integer I = new Integer(l+1);
      Double F = new Double(f[i][l]);
      Double H = new Double(pm[i].h[l]);
      bw.write(I.toString()+"\t"+F.toString()+"\t"+H.toString());
      bw.newLine();
    }
    bw.close();
    fw.close();
  }
  catch(Exception ex){
    ex.printStackTrace();
  }
}
/**
 * buffers gamma into gamBest
 */
void saveGamma(){
  gamBest = new double[n][m];
  for (int i = 0; i < n; i++){
    for (int j = 0; j < m; j++){
      gamBest[i][j] = gamma[i][j];
    }
  }
}
/**
 * estimates initial temperature (pr(upward move) = 0.5)
 * @return initial temperature
 */
double initT(){
  double res = 0.;
  bestE = getE();
  saveGamma();
  double dE = 0.;
  for (int i = 0; i < nPert; i++){
    perturb();
    dE += Math.abs(bestE-getE());
    restore();
  }
  dE /= (double) nPert;
  res = -dE/Math.log(0.5);
  return res;
}
/**
 * one random perturbation of one gamma entry and projection,
 * after buffering current gamma
```

```
 */
void perturb(){
  for (int i = 0; i < n; i++){
    for (int j = 0; j < m; j++){
      gamBuff[i][j] = gamma[i][j];
    }
  }
  gamma[1+rand.nextInt(n-1)][rand.nextInt(m)] = rand.nextDouble();
  ProjOnPolyHSet proj = new ProjOnPolyHSet(NIModel.toVector(gamma), plic.A, plic.b, rand);
  gamma = NIModel.toMatrix(proj.optimum, n);
  pm = new NIModel[nd];
  for (int i = 0; i < nd; i++){
    pm[i] = new NIModel(bbs, cs[i], gamma, f[i].length);
  }
}
/**
 * restores previous gamma when a perturbation was not accepted
 */
void restore(){
  for (int i = 0; i < n; i++){
    for (int j = 0; j < m; j++){
      gamma[i][j] = gamBuff[i][j];
    }
  }
  pm = new NIModel[nd];
  for (int i = 0; i < nd; i++){
    pm[i] = new NIModel(bbs, cs[i], gamma, f[i].length);
  }
}
/**
 * initialization of variables; gamma is set to a random matrix and projected
 */
void initModel(){
  gamma = new double[n][m];
  gamBuff = new double[n][m];
  plic = new NIModelLIC(n, bbs);
  for (int i = 0; i < n; i++){
    for (int j = 0; j < m; j++){
      gamma[i][j] = rand.nextDouble();
    }
  }
  ProjOnPolyHSet proj = new ProjOnPolyHSet(NIModel.toVector(gamma), plic.A, plic.b, rand);
  gamma = NIModel.toMatrix(proj.optimum, n);
  pm = new NIModel[nd];
  for (int i = 0; i < nd; i++){
    pm[i] = new NIModel(bbs, cs[i], gamma, f[i].length);
  }
  for (int i = 0; i < n; i++){
    for (int j = 0; j < m; j++){
      gamBuff[i][j] = gamma[i][j];
    }
  }
}
/**
 * returns the objective function: L1 distance between experimental
```

```
 * and modeled fragment length distributions
 * @return L1 distance between experimental and modeled fragment
 * length distributions
 */
double getE(){
  double res = 0.;
  for (int i = 0; i < nd; i++){
    double d = 0.;
    for (int j = 0; j < pm[i].lm; j++){
      d += Math.abs(pm[i].h[j]-f[i][j]);
    }
    res += d;
  }
  return res;
}
/**
 * loads experimental fragment length distributions
 * @param file input file
 */
void loadConstraints(String[] file){
  f = new double[nd][];
  for (int i = 0; i < nd; i++){
    Vector<String> v = new Vector<String>();
    try{
      FileReader fr = new FileReader(file[i]);
      BufferedReader br = new BufferedReader(fr);
      String line = br.readLine();
      while((line = br.readLine()) != null){
        StringTokenizer st = new StringTokenizer(line, "\t");
        if (st.countTokens() == 2){
          v.add(line);
        }
      }
      br.close();
      fr.close();
    }
    catch(Exception ex){
      ex.printStackTrace();
    }
    f[i] = new double[v.size()];
    for (int j = 0; j < v.size(); j++){
      String line = v.elementAt(j);
      StringTokenizer st = new StringTokenizer(line, "\t");
      Integer L = new Integer(st.nextToken());
      Double D = new Double(st.nextToken());
      f[i][L.intValue()-1] = D.doubleValue();
    }
  }
}
/**
 * fits N&I model without added constraint at the reducing end;
 * for supplementary material
 * @param inDir input directory (ends with "\\")
 * @param outDir output directory (ends with "\\")
 * @param rand
```

```
 */
public static void withoutREC(String inDir, String outDir){
  Random rand = new Random(1);
  BBSet bbs = new BBSet(inDir+"US.ab.txt");
  int n = 16;
  for (int s = 1; s <= 100; s++){
    Integer S = new Integer(s);
    String pref = "s"+S.toString();
    String[] specFile = new String[2];
    specFile[0] = inDir+"US.hepI.txt";
    specFile[1] = inDir+"US.hepIII.txt";
    String[] consFile = new String[2];
    consFile[0] = inDir+"hepI.f.txt";
    consFile[1] = inDir+"hepIII.f.txt";
    String[] outFile = new String[2];
    outFile[0] = outDir+"PM."+pref+".hepI.fit.res";
    outFile[1] = outDir+"PM."+pref+".hepIII.fit.res";
    String modFile = outDir+"PM."+pref+".gamma.res";
    new NIModelSA(n, bbs, specFile, consFile, outFile, modFile, rand);
  }
}
/**
 * fits N&I model without added constraint at the reducing end
 * and for different values of BKHS chain length n; for supplementary material
 * @param inDir input directory (ends with "\\")
 * @param outDir output directory (ends with "\\")
 * @param rand
 */
public static void withoutRECandN(String inDir, String outDir){
  Random rand = new Random(1);
  BBSet bbs = new BBSet(inDir+"US.ab.txt");
  for (int n = 12; n <= 20; n++){
    Integer I = new Integer(n);
    for (int s = 1; s <= 10; s++){
      Integer S = new Integer(s);
      String pref = "N"+I.toString()+"s"+S.toString();
      String[] specFile = new String[2];
      specFile[0] = inDir+"US.hepI.txt";
      specFile[1] = inDir+"US.hepIII.txt";
      String[] consFile = new String[2];
      consFile[0] = inDir+"hepI.f.txt";
      consFile[1] = inDir+"hepIII.f.txt";
      String[] outFile = new String[2];
      outFile[0] = outDir+"PM."+pref+".hepI.fit.res";
      outFile[1] = outDir+"PM."+pref+".hepIII.fit.res";
      String modFile = outDir+"PM."+pref+".gamma.res";
      new NIModelSA(n, bbs, specFile, consFile, outFile, modFile, rand);
    }
  }
}
/**
 * fits N&I model with added constraint at the reducing end
 * and for different values of BKHS chain length n; for supplementary material
 * @param inDir input directory (ends with "\\")
 * @param outDir output directory (ends with "\\")
```

```
   */
  public static void withREC(String inDir, String outDir){
    Random rand = new Random(1);
    BBSet bbs = new BBSet(inDir+"US.ab.txt");
    int n = 16;
    int[] pos = new int[1];
    pos[0] = n-1;
    int m = bbs.m;
    double[][] zeta = new double[pos.length][m];
    zeta[0][0] = 0.44;
    zeta[0][1] = 0.56;
    for (int s = 1; s <= 50; s++){
      Integer S = new Integer(s);
      String pref = "s"+S.toString();
      String[] specFile = new String[2];
      specFile[0] = inDir+"US.hepI.txt";
      specFile[1] = inDir+"US.hepIII.txt";
      String[] consFile = new String[2];
      consFile[0] = inDir+"hepI.f.txt";
      consFile[1] = inDir+"hepIII.f.txt";
      String[] outFile = new String[2];
      outFile[0] = outDir+"PMRE."+pref+".hepI.fit.res";
      outFile[1] = outDir+"PMRE."+pref+".hepIII.fit.res";
      String modFile = outDir+"PMRE."+pref+".gamma.res";
      new NIModelSA(n, bbs, specFile, consFile, outFile, modFile, zeta, pos, rand);
    }
  }
  /**
   *
   * @param args
   */
  public static void main(String[] args){
    String inDir = "input\\";
    String outDir = "output\\";
    withoutREC(inDir, outDir);
    withoutRECandN(inDir, outDir);
    withREC(inDir, outDir);
  }
}
```

```
import java.util.Vector;
/**
 * each instance gives infeasibility of the constraint set
 * (overall disaccharide composition and heparinase digest)
 * for BKHS chains of length n
 */
public class ProfileFeasibility {
  /**
   * enumerates molecular species (all possible sequences)
   */
  Species sp = null;
  /**
   * matrix for constraints
   */
  double[][] A = null;
  /**
   * vector for constraints
   */
  double[] b = null;
  /**
   * wrapper for all constraints in equality form
   */
  LinEqCons lec = null;
  /**
   * phase I of the simplex
   */
  SimplexPhaseI sp1 = null;
  /**
   * infeasibility (produced by this.sp1)
   */
  double infeasibility = 0.;
  /**
   * disaccaride labels
   */
  String[] lab = null;
  /**
   * gives infeasibility of the constraint set (overall disaccharide composition and
   * heparinase digest) for BKHS chains of length n
   * @param m number of disaccharides
   * @param n BKHS chain length
   * @param lab disaccharide labels
   * @param inDir input directory (ends with "\\")
   * @param outDir output directory (ends with "\\")
   */
  public ProfileFeasibility(int m, int n, String[] lab, String inDir, String outDir){
    this.lab = lab;
    sp = new Species(m, n);
    BBSet bbs = new BBSet(inDir+"US.ab.txt");
    String[] csFile = new String[2];
    String[] fragFile = new String[2];
    csFile[0] = new String(inDir+"US.hepI.txt");
    csFile[1] = new String(inDir+"US.hepIII.txt");
    fragFile[0] = new String(inDir+"hepI.f.txt");
```

```
    fragFile[1] = new String(inDir+"hepIII.f.txt");
    lec = sp.getCompleteLEC(bbs, csFile, fragFile);
    A = lec.A;
    b = lec.b;
    sp1 = new SimplexPhaseI(A, b);
    infeasibility = sp1.finalCost;
    System.err.println(n+"\t"+infeasibility);
  }
  /**
   * makes profile of infeasibility for BKHS chain length from 5 to 20
   * @param inDir input directory (ends with "\\")
   * @param outDir output directory (ends with "\\")
   */
  public static void makeProfile(String inDir, String outDir){
    String[] lab = new String[2];
    lab[0] = new String("U");
    lab[1] = new String("S");
    Vector<String> v = new Vector<String>();
    v.add("n\tinfeasibility");
    for (int n = 5; n <= 20; n++){
      ProfileFeasibility hf = new ProfileFeasibility(2,n,lab, inDir, outDir);
      Integer N = new Integer(n);
      Double D = new Double(hf.infeasibility);
      v.add(N.toString()+"\t"+D.toString());
    }
    Utils.saveFile(v, outDir+"Feasibility.res");
  }
  /**
   *
   * @param args
   */
  public static void main(String[] args){

    String inDir = "input\\";
    String outDir = "output\\";
    makeProfile(inDir, outDir);
  }
}
```

```
import java.util.Random;
/**
 * projection of a vector y onto a polyhedral set (Ax <= b) via coordinate
 * descent: minimize w.r.t. x ||x-y||^2 subject to Ax <= b
 */
public class ProjOnPolyHSet {
  /**
   * certificate (false if coordinate descent was too slow)
   */
  boolean success = false;
  /**
   * projection of y onto polyhedral set Ax <= b
   */
  public double[] optimum = null;
  /**
   * projection of a vector y onto a polyhedral set (Ax <= b) via coordinate
   * descent: minimize w.r.t. x ||x-y||^2 subject to Ax <= b
   * @param y vector to project
   * @param A matrix in inequality constraints Ax <= b
   * @param b vector in inequality constraints Ax <= b
   * @param rand used to seed the coordinate descent
   */
  public ProjOnPolyHSet(double[] y, double[][] A, double[] b, Random rand){
    double[][] AT = MatrixOp.transpose(A);
    double[][] P = MatrixOp.multMat(A, AT);
    for (int i = 0; i < P.length; i++){
      for (int j = 0; j < P[i].length; j++){
        P[i][j] *= 0.5;
      }
    }
    double[] t = MatrixOp.multMatVec(A, y);
    for (int j = 0; j < t.length; j++){
      t[j] = b[j]-t[j];
    }
    double[] s = new double[P.length];
    for (int j = 0; j < s.length; j++){
      s[j] = rand.nextDouble();
    }
    CoordDescNNQP cd = new CoordDescNNQP(P, t, s);
    success = cd.success;
    optimum = MatrixOp.multMatVec(AT, cd.optimum);
    for (int j = 0; j < optimum.length; j++){
      optimum[j] = y[j]-0.5*optimum[j];
    }
  }
}
```

```
import java.util.*;
/**
 * convenience class to sort pairs
 */
public class SandVal implements Comparator<Object>{
  /**
   * String field
   */
    public String s = null;
    /**
     * double field, used for sorting
     */
    double val = 0.;
    /**
     * 1 for sorting by ascending values and -1 by descending values
     */
    int sign = 1;
    /**
     * convenience class to sort two arrays, based on values in one,
     * while preserving pairs
     * @param s String (label)
     * @param val value to sort by
     * @param dir direction (ascending "a" or descending "d")
     */
    public SandVal(String s, double val, String dir){
    this.s = s;
    this.val = val;
    if (dir.toLowerCase().startsWith("a"))
        sign = 1;
    else
        sign = -1;
    }
    /**
     * defines order
     */
    public int compare(Object o1, Object o2){
      SandVal sv1 = (SandVal) o1;
      SandVal sv2 = (SandVal) o2;
    if (sv1.val == sv2.val){
        return(0);
    }
    else{
        if (sv1.val < sv2.val){
          return(-1*sign);
        }
        else{
          return(sign);
        }
      }
    }
}
```

```
/**
 * This class represents a linear constraint Ax = b in standard form:
 * matrix A (m x n) has rank m < n and b >= 0.
 */
public class SFormCons {
  /**
   * matrix for linear equality Ax = b
   */
  double[][] A = null;
  /**
   * vector in constraint Ax = b, matrix A being transformed
   * so that b >= 0
   */
  double[] b = null;
  /**
   * number of columns
   */
  int n = 0;
  /**
   * number of rows (rank of A)
   */
  int m = 0;
  /**
   * creates a linear equality constraint in standard form (Ax = b with
   * b >= 0) starting from a m x n matrix A (of rank m) and a vector b
   * @param mA m x n matrix of rank m < n
   * @param mb m dimensional vector
   */
  public SFormCons(double[][] mA, double[] mb){
    m = mA.length;
    n = mA[0].length;
    A = new double[m][n];
    b = new double[m];
    for (int i = 0; i < m; i++){
      if (mb[i] >= 0.){
        b[i] = mb[i];
        for (int j = 0; j < n; j++){
          A[i][j] = mA[i][j];
        }
      }
      else{
        b[i] = -mb[i];
        for (int j = 0; j < n; j++){
          A[i][j] = -mA[i][j];
        }
      }
    }
  }
  /**
   * prints out the constraint
   */
  void print(){
    for (int i = 0; i < m; i++){
```

```
      for (int j = 0; j < n; j++){
        System.out.print(A[i][j]+"\t");
      }
      System.out.println(b[i]);
    }
  }
  /**
   * for testing
   * @param args
   */
  public static void main(String[] args){
    double[][] mA = new double[2][3];
    double[] mb = new double[2];
    mA[0][0] = 1.;
    mA[0][1] = 2.;
    mA[0][2] = -3.;
    mb[0] = -4.;
    mA[1][0] = 5.;
    mA[1][1] = 6.;
    mA[1][2] = -7.;
    mb[1] = -8.;
    SFormCons sfc = new SFormCons(mA, mb);
    sfc.print();
  }
}
```

```
import java.util.Enumeration;
import java.util.Hashtable;
import java.util.Iterator;
import java.util.TreeSet;
/**
 * simplex method for linear programming (phase II)
 */
public class Simplex {
  /**
   * simplex tableau
   */
  Tableau tab = null;
  /**
   * values of certificate are: -1 for no feasible point, 0 for unbounded
   * below in phase II and 1 for optimum found
   */
  int certificate = -3;
  /**
   * certificate in words
   */
  String certificateString = null;
  /**
   * buffer copy of the tableau
   */
  double[][] y = null;
  /**
   * basic variables are always kept in the first m columns,
   * this hashtable maps variable indices to column indices
   */
  Hashtable<Integer,Integer> var2col = new Hashtable<Integer,Integer>();
  /**
   * basic variables are always kept in the first m columns,
   * this hashtable maps column indices to variable indices
   */
  Hashtable<Integer,Integer> col2var = new Hashtable<Integer,Integer>();
  /**
   * used to keep track of which variables indices are basic,
   * in order to implement Bland's rule
   */
  TreeSet<Integer> basic = new TreeSet<Integer>();
  /**
   * used to keep track of which variables indices are nonbasic,
   * in order to implement Bland's rule
   */
  TreeSet<Integer> nonbasic = new TreeSet<Integer>();
  /**
   * feasible point obtained at the end of phaseI
   */
  double[] feasiblePoint = null;
  /**
   * the point at the end of phase II
   */
  public double[] optimum = null;
```

```java
/**
 * inconsistency threshold for phase II
 */
double epsilon = 1e-5;
/**
 * number of constraints in phase II
 */
int m = 0;
/**
 * number of variables in phase II
 */
int n = 0;
/**
 * solution, i.e. final cost
 */
double finalCost = 0.;
/**
 * simplex method for linear programming
 * @param sp1 phase I of the simplex (tableau)
 * @param c vector of cost coefficients
 */
public Simplex(SimplexPhaseI sp1, double[] c){
  init(sp1, c);
  boolean phaseII = true;
  while(phaseII){
    int q = getQ();
    int p = -1;
    if (q == -1){
      phaseII = false;
    }
    else{
      p = getP(q);
      if (p == -1){
        certificate = -3;
        phaseII = false;
      }
      else{
        pivot(p, q);
        saveTab();
      }
    }
  }
  optimum = new double[n];
  Iterator<Integer> it = basic.iterator();
  while(it.hasNext()){
    Integer var = it.next();
    Integer col = var2col.get(var);
    optimum[var.intValue()] = tab.y[col.intValue()][tab.n];
  }
  finalCost = 0.;
  for (int i = 0; i < c.length; i++){
    finalCost += c[i]*optimum[i];
  }
}
/**
```

```
 * cost = objective function value
 * @return objective function value
 */
public double getCost(){
  return -tab.y[tab.y.length-1][tab.y[0].length-1];
}
/**
 * pivoting operation in the tableau
 * @param p index of the column to leave the basis
 * @param q index of the column to enter the basis
 */
void pivot(int p, int q){
  for (int i = 0; i < tab.nrows; i++){
    if (i == p){
      for (int j = 0; j < tab.ncols; j++){
        tab.y[p][j] = y[p][j]/y[p][q];
      }
    }
    else{
      for (int j = 0; j < tab.ncols; j++){
        tab.y[i][j] = y[i][j]-y[p][j]*y[i][q]/y[p][q];
      }
    }
  }
  swapColumns(p, q);
}
/**
 * swaps columns p and q in the tableau and updates mappings
 * between column and variable indices
 * @param p index of the column to leave the basis
 * @param q index of the column to enter the basis
 */
void swapColumns(int p, int q){
  double[] buf = new double[tab.y.length];
  for (int i = 0; i < buf.length; i++){
    buf[i] = tab.y[i][p];
  }
  for (int i = 0; i < buf.length; i++){
    tab.y[i][p] = tab.y[i][q];
  }
  for (int i = 0; i < buf.length; i++){
    tab.y[i][q] = buf[i];
  }
  Integer I = col2var.get(new Integer(p));
  int varp = I.intValue();
  I = col2var.get(new Integer(q));
  int varq = I.intValue();
  col2var.put(new Integer(p), new Integer(varq));
  var2col.put(new Integer(varq), new Integer(p));
  col2var.put(new Integer(q), new Integer(varp));
  var2col.put(new Integer(varp), new Integer(q));
  basic.remove(new Integer(varp));
  nonbasic.add(new Integer(varp));
  nonbasic.remove(new Integer(varq));
  basic.add(new Integer(varq));
```

```
}
/**
 * Given the index q of the column to enter next the basic set, finds the
 * index q of the column to leave the basic set, or -1 if the linear
 * problem is unbounded below. This method also implements Bland's rule
 * @param q index of the column to enter the basic set
 * @return index of the column to leave the basis
 */
int getP(int q){
  int res = -1;
  double min = 0.;
  boolean first = true;
  Iterator<Integer> it = basic.iterator();
  while (it.hasNext()){
    Integer var = it.next();
    Integer I = var2col.get(var);
    int row = I.intValue();
    if (tab.y[row][q] > 0.){
      if (first){
        min = tab.y[row][tab.n]/tab.y[row][q];
        first = false;
        res = row;
      }
      else{
        if (tab.y[row][tab.n]/tab.y[row][q] < min){
          min = tab.y[row][tab.n]/tab.y[row][q];
          res = row;
        }
      }
    }
  }
  return res;
}
/**
 * returns the index q of the column to enter the basic set in
 * order to lower the cost, or -1 if the current set of basic
 * variables is optimal
 * @return the index q of the column to enter the basis
 */
int getQ(){
  int res = -1;
  double minr = 0.;
  Iterator<Integer> it = nonbasic.iterator();
  while(it.hasNext()){
    Integer var = it.next();
    Integer I = var2col.get(var);
    int col = I.intValue();
    if (tab.y[tab.m][col] < minr){
      minr = tab.y[tab.m][col];
      res = col;
    }
  }
  return res;
}
/**
```

```
   * initialization
   * @param sp1 output of phase I
   * @param c vector of cost coefficients
   */
  void init(SimplexPhaseI sp1, double[] c){
    m = sp1.m;
    n = sp1.n;
    tab = new Tableau(sp1, c);
    y = new double[tab.y.length][tab.y[0].length];
    saveTab();
    col2var = new Hashtable<Integer,Integer>();
    var2col = new Hashtable<Integer,Integer>();
    Enumeration<Integer> en = sp1.col2var.keys();
    while(en.hasMoreElements()){
      Integer col = en.nextElement();
      Integer var = sp1.col2var.get(col);
      col2var.put(col, var);
      var2col.put(var, col);
    }
    basic = new TreeSet<Integer>();
    nonbasic = new TreeSet<Integer>();
    Iterator<Integer> it = sp1.basic.iterator();
    while(it.hasNext()){
      basic.add(it.next());
    }
    it = sp1.nonbasic.iterator();
    while(it.hasNext()){
      nonbasic.add(it.next());
    }
    feasiblePoint = new double[n];
    for (int j = 0; j < n; j++){
      feasiblePoint[j] = sp1.feasiblePoint[j];
    }
  }
  /**
   * buffers the current tableau
   */
  void saveTab(){
    for (int i = 0; i < tab.nrows; i++){
      for (int j = 0; j < tab.ncols; j++){
        y[i][j] = tab.y[i][j];
      }
    }
  }
}
```

```
import java.util.Enumeration;
import java.util.HashSet;
import java.util.Hashtable;
import java.util.Iterator;
import java.util.Random;
import java.util.TreeSet;
/**
 * implementation of the Simplex method, but limited to phase I implemented
 * via addition of m artificial variables
 */
public class SimplexPhaseI {
  /**
   * contains the linear constraint Ax = b in standard for, that is,
   * if some elements of b were negative, they are turned positive and the
   * corresponding row of A is multiplied by -1
   */
  SFormCons sfc = null;
  /**
   * simplex tableau
   */
  Tableau tab = null;
  /**
   * values of certificate are:
   * -1 for no feasible point, 0 for unbounded below in phase II and 1
   * for optimum found
   */
  int certificate = -3;
  /**
   * certificate in words
   */
  public String certificateString = null;
  /**
   * the largest absolute value among artificial variables at the end
   * of phase I (feasible problem only if absAVmax < epsilon)
   */
  double absAVmax = 0.;
  /**
   * buffer of the tableau
   */
  double[][] y = null;
  /**
   * basic variables are always kept in the first m columns, this hashtable
   * maps variable indices to column indices
   */
  Hashtable<Integer,Integer> var2col = new Hashtable<Integer,Integer>();
  /**
   * basic variables are always kept in the first m columns, this hashtable
   * maps column indices to variable indices
   */
  Hashtable<Integer,Integer> col2var = new Hashtable<Integer,Integer>();
  /**
   * used to keep track of which variables indices are basic,
   * in order to implement Bland's rule
```

```
 */
TreeSet<Integer> basic = new TreeSet<Integer>();
/**
 * used to keep track of which variables indices are nonbasic,
 * in order to implement Bland's rule
 */
TreeSet<Integer> nonbasic = new TreeSet<Integer>();
/**
 * keeps track of indices of the artificial variables
 */
HashSet<Integer> artificial = new HashSet<Integer>();
/**
 * the point obtained at the end of phaseI, feasible or not
 */
double[] phaseIPoint = null;
/**
 * same as phaseIpoint but omitting the artificial variables
 */
public double[] feasiblePoint = null;
/**
 * inconsistency threshold for phase I
 */
double epsilon = 1e-14;
/**
 * number of constraints
 */
int m = 0;
/**
 * number of variables in phase II (i.e. n is n+m in phase I)
 */
public int n = 0;
/**
 * at the end of phase I an initial tableau for phase II is created;
 * note that this tableau does not have a last row for relative costs
 */
double[][] phaseIITableau = null;
/**
 * final infeasibility
 */
double finalCost = 0.;
/**
 * implementation of the Simplex method, limited to phase I
 * implemented via addition of m artificial variables
 * @param A matrix in constraint Ax = b
 * @param b vector in constraint Ax = b
 */
public SimplexPhaseI(double[][] A, double[] b){
  sfc = new SFormCons(A, b);
  m = A.length;
  n = A[0].length; // for phase II only, phase I uses tab.n
  // Phase I
  AVSFormCons avsfc = new AVSFormCons(A, b);
  tab = new Tableau(avsfc);
  y = new double[tab.y.length][tab.y[0].length];
  saveTab();
```

```
  for (int i = 0; i < tab.n; i++){
    col2var.put(new Integer(i), new Integer(i));
    var2col.put(new Integer(i), new Integer(i));
    if (i < tab.m){
      basic.add(new Integer(i));
      artificial.add(new Integer(i));
    }
    else
      nonbasic.add(new Integer(i));
  }
  boolean phaseI = true;
  while(phaseI){
    int q = getQ();
    int p = -1;
    if (q == -1){
      phaseI = false;
    }
    else{
      p = getP(q);
      if (p == -1){
        certificate = -3;
        phaseI = false;
      }
      else{
        pivot(p, q);
        saveTab();
      }
    }
    double cost = getCost();
    finalCost = Math.abs(cost);
    if (cost < 1e-13){
      phaseI = false;
    }
  }
  fixDegeneracy();
  makePhaseIPoint();
  makeFeasiblePoint();
  updateForPhaseII();
}
/**
 * creates an initial tableau for phase II and updates mapping
 * between variables and columns
 */
void updateForPhaseII(){
  Hashtable<Integer,Integer> col2varB = new Hashtable<Integer,Integer>();
  TreeSet<Integer> basicB = new TreeSet<Integer>();
  TreeSet<Integer> nonbasicB = new TreeSet<Integer>();
  phaseIITableau = new double[m][n+1];
  // copy of the tableau (without last row) and removal of artificial variables
  int col = -1;
  for (int j = 0; j < tab.n; j++){
    Integer J = new Integer(j);
    Integer var = col2var.get(J);
    if (!artificial.contains(var)){
      col++;
```

```
      Integer varB = new Integer(var.intValue()-tab.m);
      col2varB.put(new Integer(col), varB);
      for (int i = 0; i < tab.m; i++){
        phaseIITableau[i][col] = tab.y[i][j];
      }
      if (j < tab.m){
        basicB.add(varB);
      }
      else{
        nonbasicB.add(varB);
      }
    }
  }
  for (int i = 0; i < tab.m; i++){
    phaseIITableau[i][n] = tab.y[i][tab.n];
  }
  // update of index mappings
  col2var = new Hashtable<Integer,Integer>();
  var2col = new Hashtable<Integer,Integer>();
  basic = new TreeSet<Integer>();
  nonbasic = new TreeSet<Integer>();
  Enumeration<Integer> en = col2varB.keys();
  while(en.hasMoreElements()){
    Integer cl = en.nextElement();
    Integer var = col2varB.get(cl);
    col2var.put(cl, var);
    var2col.put(var, cl);
  }
  Iterator<Integer> it = basicB.iterator();
  while(it.hasNext()){
    basic.add(it.next());
  }
  it = nonbasicB.iterator();
  while(it.hasNext()){
    nonbasic.add(it.next());
  }
}
/**
 * saves the found feasible point; note that fixDegeneracy() and
 * makePhaseIPoint() must have been called before
 */
void makeFeasiblePoint(){
  certificateString = "feasible";
  feasiblePoint = new double[n];
  for (int j = 0; j < n; j++){
    feasiblePoint[j] = phaseIPoint[m+j];
  }
}
/**
 * saves the last point of phase I (including artificial variables)
 */
void makePhaseIPoint(){
  phaseIPoint = new double[tab.n];
  Iterator<Integer> it = basic.iterator();
  while(it.hasNext()){
```

```
    Integer var = it.next();
    Integer col = var2col.get(var);
    phaseIPoint[var.intValue()] = tab.y[col.intValue()][tab.n];
  }
}
/**
 * in case of degeneracy some of the final basic variables might still be artificial
 * variables; if so, these basic variables must be swapped with nonbasic variables
 * that are not artificial variables; this is performed by this method, updating also
 * mappings between tableau columns and variable indices
 */
void fixDegeneracy(){
  HashSet<Integer> toswap = new HashSet<Integer>();
  Iterator<Integer> it = basic.iterator();
  while(it.hasNext()){
    Integer var = it.next();
    if (artificial.contains(var)){
      toswap.add(var);
    }
  }
  if (toswap.size() > 0){
    it = toswap.iterator();
    while(it.hasNext()){
      Integer I = it.next();
      Integer P = var2col.get(I);
      Integer Q = null;
      Iterator<Integer> it2 = nonbasic.iterator();
      while(it2.hasNext()){
        Integer J = it2.next();
        if (!artificial.contains(J)){
          Q = var2col.get(J);
          break;
        }
      }
      pivot(P.intValue(), Q.intValue());
    }
  }
}
/**
 * the current cost (objective function value)
 * @return the current cost
 */
double getCost(){
  return -tab.y[tab.y.length-1][tab.y[0].length-1];
}
/**
 * pivoting operation in the tableau
 * @param p index of the column to leave the basis
 * @param q index of the column to enter the basis
 */
void pivot(int p, int q){
  for (int i = 0; i < tab.nrows; i++){
    if (i == p){
      for (int j = 0; j < tab.ncols; j++){
        tab.y[p][j] = y[p][j]/y[p][q];
```

```
        }
      }
      else{
        for (int j = 0; j < tab.ncols; j++){
          tab.y[i][j] = y[i][j]-y[p][j]*y[i][q]/y[p][q];
        }
      }
    }
    swapColumns(p, q);
  }
  /**
   * swaps columns p and q in the tableau and updates mappings
   * between column and variable indices
   * @param p index of the column to leave the basis
   * @param q index of the column to enter the basis
   */
  void swapColumns(int p, int q){
    double[] buf = new double[tab.y.length];
    for (int i = 0; i < buf.length; i++){
      buf[i] = tab.y[i][p];
    }
    for (int i = 0; i < buf.length; i++){
      tab.y[i][p] = tab.y[i][q];
    }
    for (int i = 0; i < buf.length; i++){
      tab.y[i][q] = buf[i];
    }
    Integer I = col2var.get(new Integer(p));
    int varp = I.intValue();
    I = col2var.get(new Integer(q));
    int varq = I.intValue();
    col2var.put(new Integer(p), new Integer(varq));
    var2col.put(new Integer(varq), new Integer(p));
    col2var.put(new Integer(q), new Integer(varp));
    var2col.put(new Integer(varp), new Integer(q));
    basic.remove(new Integer(varp));
    nonbasic.add(new Integer(varp));
    nonbasic.remove(new Integer(varq));
    basic.add(new Integer(varq));
  }
  /**
   * Given the index q of the column to enter next the basic set, finds the
   * index q of the column to leave the basic set, or -1 if the linear
   * problem is unbounded below. This method also implements Bland’s rule
   * @param q index of the column to enter the basic set
   * @return index of the column to leave the basis
   */
  int getP(int q){
    int res = -1;
    double min = 0.;
    boolean first = true;
    Iterator<Integer> it = basic.iterator();
    while (it.hasNext()){
      Integer var = it.next();
      Integer I = var2col.get(var);
```

```
    int row = I.intValue();
    if (tab.y[row][q] > 0.){
      if (first){
        min = tab.y[row][tab.n]/tab.y[row][q];
        first = false;
        res = row;
      }
      else{
        if (tab.y[row][tab.n]/tab.y[row][q] < min){
          min = tab.y[row][tab.n]/tab.y[row][q];
          res = row;
        }
      }
    }
  }
  return res;
}
/**
 * returns the index q of the column to enter the basic set in
 * order to lower the cost, or -1 if the current set of basic
 * variables is optimal.
 * @return the index q of the column to enter the basis
 */
int getQ(){
  int res = -1;
  double minr = 0.;
  Iterator<Integer> it = nonbasic.iterator();
  while(it.hasNext()){
    Integer var = it.next();
    Integer I = var2col.get(var);
    int col = I.intValue();
    if (tab.y[tab.m][col] < minr){
      minr = tab.y[tab.m][col];
      res = col;
    }
  }
  return res;
}
/**
 * buffers the current tableau
 */
void saveTab(){
  for (int i = 0; i < tab.nrows; i++){
    for (int j = 0; j < tab.ncols; j++){
      y[i][j] = tab.y[i][j];
    }
  }
}
/**
 * for testing
 * @param args
 */
public static void main(String[] args){
  int n = 100;
  int m = 8;
```

```
    Random rand = new Random();
    double[][] A = new double[m][n];
    double[] b = new double[m];
    double[] x = new double[n];
    double sum = 0.;
    for (int j = 0; j < n; j++){
      x[j] = rand.nextDouble();
      sum += x[j];
    }
    for (int j = 0; j < n; j++){
      x[j] /= sum;
    }
    for (int i = 0; i < m; i++){
      b[i] = 0.;
      for (int j = 0; j < n; j++){
        A[i][j] = rand.nextDouble();
        b[i] += x[j]*A[i][j];
      }
    }
    SimplexPhaseI sp1 = new SimplexPhaseI(A, b);
    System.out.println(sp1.finalCost);
    double[] c = new double[n];
    for (int j = 0; j < n; j++){
      c[j] = 1.;
    }
    Simplex sp = new Simplex(sp1, c);
    System.out.println(sp.finalCost);
  }
}
```

```
import java.util.StringTokenizer;
import java.util.Vector;
/**
 * enumeration of all possible sequences of length n with m disaccharides
 */
public class Species {
  /**
   * chain length
   */
  int n = 0;
  /**
   * number of building blocks
   */
  int m = 0;
  /**
   * number of species = m^n
   */
  int N = 0;
  /**
   * seq[i] is the sequence of species i
   */
  int[][] seq = null;
  /**
   * enumeration of all possible sequences of length n with m disaccharides
   * @param m number of disaccharides
   * @param n BKHS chain length
   */
  public Species(int m, int n){
    this.m = m;
    this.n = n;
    N = (int) Math.pow((double) m, (double) n);
    seq = new int[N][n];
    int[] buff = new int[n];
    int pos = 0;
    for (int s = 1; s < N; s++){
      pos = 0;
      boolean kg = true;
      while(kg){
        buff[pos]++;
        if (buff[pos] < m){
          kg = false;
        }
        else{
          buff[pos] = 0;
          pos++;
        }
      }
      for (int i = 0; i < n; i++){
        seq[s][i] = buff[i];
      }
    }
  }
  /**
```

```
 * linear constraint for homogeneity of disaccharide composition along chains
 * @param bbs disaccharide set
 * @return linear constraint for homogeneity of disaccharide composition
 * along chains
 */
LinEqCons getHomogeneityLEC(BBSet bbs){
  double[][] A = new double[(n-1)*(m-1)][N];
  double[] b = new double[(n-1)*(m-1)];
  String[] tp = new String[(n-1)*(m-1)];
  int r = -1;
  for (int i = 1; i < n; i++){
    for (int j = 0; j < m-1; j++){
      r++;
      b[r] = bbs.rho[j];
      tp[r] = new String("hom");
      for (int s = 0; s < N; s++){
        if (seq[s][i] == j){
          A[r][s] = 1.;
        }
      }
    }
  }
  return new LinEqCons(A, b, tp);
}
/**
 * linear constraint for one heparinase digest (fragments lengths f[])
 * @param f distribution of fragment lengths
 * @param cs cleavage specificities of one heparinase
 * @param bbs disaccharide set
 * @return linear constraint for one heparinase digest
 */
LinEqCons getFragLEC(double[] f, CSpec cs, BBSet bbs){
  double[][] A = new double[f.length][N];
  double[] b = new double[f.length];
  String[] lab = new String[f.length];
  for (int i = 0; i < b.length; i++){
    b[i] = f[i];
    lab[i] = new String("frag");
  }
  int lm = f.length;
  for (int l = 0; l < lm-1; l++){
    for (int s = 0; s < N; s++){
      A[l][s] = getFragContrib(s, l+1, cs);
    }
  }
  for (int s = 0; s < N; s++){
    A[lm-1][s] = 0.;
    for (int l = lm-1; l < n-1; l++){
      A[lm-1][s] += getFragContrib(s, l+1, cs);
    }
  }
  double denom = 0.;
  for (int i = 0; i < bbs.m; i++){
    denom += bbs.rho[i]*cs.c[i];
  }
```

```
    denom *= (double) (n-1);
    for (int i = 0; i < A.length; i++){
      for (int j = 0; j < A[i].length; j++){
        A[i][j] /= denom;
      }
    }
    return new LinEqCons(A, b, lab);
  }
  /**
   * contribution of species s to fragments of length ll
   * @param s species (sequence) index
   * @param ll fragment length
   * @param cs cleavage specificities
   * @return contribution of species s to fragments of length ll
   */
  double getFragContrib(int s, int ll, CSpec cs){
    double res = 0.;
    if (ll > n){
      return res;
    }
    for (int i = 1; i < n-ll; i++){
      double t = cs.c[seq[s][i]];
      for (int j = 1; j <= ll-1; j++){
        t *= (1.-cs.c[seq[s][i+j]]);
      }
      t *= cs.c[seq[s][i+ll]];
      res += t;
    }
    double t = cs.c[seq[s][n-ll]];
    for (int j = n-ll+1; j < n; j++){
      t *= (1.-cs.c[seq[s][j]]);
    }
    res += t;
    return res;
  }
  /**
   * linear constraint for overall disaccharide composition
   * @param rho overall disaccharide composition
   * @return linear constraint for overall disaccharide composition
   */
  LinEqCons getCompLEC(double[] rho){
    int m = rho.length;
    double[][] A = new double[m][N];
    double[] b = new double[m];
    String[] lab = new String[m];
    for (int i = 0; i < m; i++){
      b[i] = rho[i];
      lab[i] = new String("comp");
      for (int s = 0; s < N; s++){
        for (int j = 1; j < n; j++){
          if (seq[s][j] == i){
            A[i][s] += 1.;
          }
        }
        A[i][s] /= (double) (n-1);
```

```
    }
  }
  return new LinEqCons(A, b, lab);
}
/**
 * linear constraint for normalization (sum to 1)
 * @return linear constraint for normalization (sum to 1)
 */
LinEqCons getNormLEC(){
  double[][] A = new double[1][N];
  double[] b = new double[1];
  String[] lab = new String[1];
  b[0] = 1.;
  lab[0] = new String("norm");
  for (int i = 0; i < N; i++){
    A[0][i] = 1.;
  }
  return new LinEqCons(A, b, lab);
}
/**
 * vector of individual species abundances; this method is based on a
 * N&I model and utilized to numerically check linear constraints
 * @param gamma matrix Gamma in model N&I
 * @return vector of individual species abundances under N&I with Gamma
 */
double[] getP(double[][] gamma){
  double[] res = new double[N];
  for (int s = 0; s < N; s++){
    res[s] = 1.;
    for (int i = 0; i < n; i++){
      res[s] *= gamma[i][seq[s][i]];
    }
  }
  return res;
}
/**
 * complete set of linear constraints (sum-to-1, overall disaccharide
 * composition and fragment length distributions)
 * @param bbs disaccharide set
 * @param csFile files of cleavage specificities
 * @param fragFile files of fragment length distributions
 * @return complete set of linear constraints (sum-to-1, overall
 * disaccharide composition and fragment length distributions)
 */
LinEqCons getCompleteLEC(BBSet bbs, String[] csFile, String[] fragFile){
  Vector<LinEqCons> v = new Vector<LinEqCons>();
  v.add(getNormLEC());
  v.add(LinEqCons.removeLastRow(getCompLEC(bbs.rho)));
  for (int i = 0; i < csFile.length; i++){
    CSpec cs = new CSpec(csFile[i], bbs);
    double[] f = loadFragAbund(fragFile[i]);
    v.add(LinEqCons.removeLastRow(getFragLEC(f, cs, bbs)));
  }
  return new LinEqCons(v);
}
```

```
/**
 * loads matrix Gamma of model N&I from a file;
 * used for testing of linear constraints
 * @param file ASCII file with one header row: position \t u \t s
 * @return matrix Gamma of a N&I model
 */
public static double[][] loadGamma(String file){
  Vector<String> v = Utils.loadFileNoheader(file);
  StringTokenizer st = new StringTokenizer(v.elementAt(0), "\t");
  int m = st.countTokens()-1;
  int n = v.size();
  double[][] res = new double[n][m];
  for (int i = 0; i < n; i++){
    st = new StringTokenizer(v.elementAt(i), "\t");
    st.nextToken();
    int j = -1;
    while(st.hasMoreTokens()){
      j++;
      Double D = new Double(st.nextToken());
      res[i][j] = D.doubleValue();
    }
  }
  return res;
}
/**
 * loads fragment length abundances for one heparinase digest
 * @param file ASCII file with header row: length \t abundance;
 * note that lengths are assumed to be in ascending order, the last row
 * ll represents abundances of lengths at least ll and abundances must sum to 1
 * @return fragment length abundances for one heparinase digest
 */
public static double[] loadFragAbund(String file){
  Vector<String> v = Utils.loadFileNoheader(file);
  StringTokenizer st = new StringTokenizer(v.elementAt(0), "\t");
  int n = v.size();
  double[] res = new double[n];
  for (int i = 0; i < n; i++){
    st = new StringTokenizer(v.elementAt(i), "\t");
    st.nextToken();
    Double D = new Double(st.nextToken());
    res[i] = D.doubleValue();
  }
  return res;
}
/**
 * testing of the sum-to-1 constraint, with a N&I model
 * @param inDir input directory (ends with "\\")
 */
public static void testNormCons(String inDir){
  System.out.println("testNormCons");
  Species sp = new Species(2,16);
  double[] p = sp.getP(loadGamma(inDir+"GammaExample.txt"));
  LinEqCons lec = sp.getNormLEC();
  double[] b = MatrixOp.multMatVec(lec.A, p);
  for (int i = 0; i < b.length; i++){
```

```
      System.out.println(b[i]+"\t"+lec.b[i]);
    }
    System.out.println("*****");
  }
  /**
   * testing of the constraint for overall disaccharide composition,
   * with a N&I model
   * @param inDir input directory (ends with "\\")
   */
  public static void testCompCons(String inDir){
    System.out.println("testCompCons");
    String file = inDir+"US.ab.txt";
    BBSet bbs = new BBSet(file);
    Species sp = new Species(2,16);
    double[] p = sp.getP(loadGamma(inDir+"GammaExample.txt"));
    LinEqCons lec = sp.getCompLEC(bbs.rho);
    double[] b = MatrixOp.multMatVec(lec.A, p);
    for (int i = 0; i < b.length; i++){
      System.out.println(b[i]+"\t"+lec.b[i]);
    }
    System.out.println("*****");
  }
  /**
   * testing of the constraints for fragment length distributions,
   * with a N&I model; note that since N&I cannot reproduce fragment
   * length distributions, values of constraints are calculated
   * from the N&I model
   * @param inDir input directory (ends with "\\")
   */
  public static void testFragCons(String inDir){
    System.out.println("testFragCons");
    String file = inDir+"US.ab.txt";
    BBSet bbs = new BBSet(file);
    Species sp = new Species(2,16);
    double[][] gamma = loadGamma(inDir+"GammaExample.txt");
    double[] p = sp.getP(gamma);
    System.out.println("hepI (based on N&I model)");
    CSpec cs = new CSpec(inDir+"US.hepI.txt", bbs);
    NIModel nim = new NIModel(bbs, cs, gamma, 11);
    double[] f = nim.h;
    LinEqCons lec = sp.getFragLEC(f, cs, bbs);
    double[] b = MatrixOp.multMatVec(lec.A, p);
    for (int i = 0; i < b.length; i++){
      System.out.println(b[i]+"\t"+lec.b[i]);
    }
    System.out.println("*****");
    System.out.println("hepIII (based on N&I model)");
    cs = new CSpec(inDir+"US.hepIII.txt", bbs);
    nim = new NIModel(bbs, cs, gamma, 6);
    f = nim.h;
    lec = sp.getFragLEC(f, cs, bbs);
    b = MatrixOp.multMatVec(lec.A, p);
    for (int i = 0; i < b.length; i++){
      System.out.println(b[i]+"\t"+lec.b[i]);
    }
```

```
    System.out.println("*****");
  }
  /**
   * testing of homogeneity constraint with model H&I
   * @param inDir input directory (ends with "\\")
   */
  public static void testHomogeneityCons(String inDir){
    BBSet bbs = new BBSet(inDir+"US.ab.txt");
    Species sp = new Species(2,16);
    LinEqCons lec = sp.getHomogeneityLEC(bbs);
    SimplexPhaseI sp1 = new SimplexPhaseI(lec.A, lec.b);
    System.out.println("infeasibility = "+sp1.finalCost);
    System.out.println("*****");
    int N = sp.N;
    double[] p = new double[N];
    for (int s = 0; s < N; s++){
      p[s] = 1.;
      for (int i = 0; i < sp.n; i++){
        p[s] *= bbs.rho[sp.seq[s][i]];
      }
    }
    double[] b = MatrixOp.multMatVec(lec.A, p);
    for (int i = 0; i < b.length; i++){
      System.out.println(b[i]+"\t"+lec.b[i]);
    }
  }
  /**
   * testing of computed linear constraints with N&I or H&I model
   * @param inDir input directory (ends with "\\")
   */
  public static void testing(String inDir){
    testNormCons(inDir);
    testCompCons(inDir);
    testFragCons(inDir);
    testHomogeneityCons(inDir);
  }
  /**
   * for testing
   * @param args
   */
  public static void main(String[] args){
    String inDir = "input\\";
    testing(inDir);
  }
}
```

```
/**
 * simplex tableau
 */
public class Tableau {
  /**
   * number of columns in the tableau: n+1
   */
  int ncols = 0;
  /**
   * number of variables
   */
  int n = 0;
  /**
   * number of rows in the tableau: m+1
   */
  int nrows = 0;
  /**
   * number of constraints
   */
  int m = 0;
  /**
   * simplex tableau
   */
  double[][] y = null;
  /**
   * cost coefficients (the objective function)
   */
  double[] c = null;
  /**
   * constructor for phase I: creates a simplex tableau based on a constraint in
   * canonical form (linear constraint in standard form with added artificial variables)
   * @param avsfc linear constraint in standard form with added artificial variables
   * @param c cost coefficients
   */
  public Tableau(AVSFormCons avsfc){
    m = avsfc.m;
    nrows = m+1;
    n = avsfc.n;
    ncols = n+1;
    this.c = new double[n];
    for (int j = 0; j < m; j++){
      c[j] = 1.;
    }
    y = new double[nrows][ncols];
    for (int i = 0; i < m; i++){
      for (int j = 0; j < n; j++){
        y[i][j] = avsfc.A[i][j];
      }
      y[i][n] = avsfc.b[i];
    }
    for (int j = 0; j < n; j++)
      y[m][j] = c[j];
    reduce();
```

```
}
/**
 * constructor for phase II based on the results of phase I
 * @param sp1 phase I tableau
 * @param coef cost coefficients for phase II
 */
public Tableau(SimplexPhaseI sp1, double[] coef){
  m = sp1.m;
  nrows = m+1;
  n = sp1.n;
  ncols = n+1;
  c = new double[n];
  for (int j = 0; j < n; j++){
    Integer J = new Integer(j);
    Integer col = sp1.var2col.get(J);
    c[col.intValue()] = coef[j];
  }
  y = new double[nrows][ncols];
  for (int i = 0; i < sp1.m; i++){
    for (int j = 0; j < ncols; j++){
      y[i][j] = sp1.phaseIITableau[i][j];
    }
  }
  for (int j = 0; j < n; j++)
    y[m][j] = c[j];
  reduce();
}
/**
 * constructor for phase II based on an array-tableau
 * already in canonical form, at the exception of the last row
 * (cost coefficients) which is reduced at the end of this constructor
 * @param t array-tableau in canonical form
 */
public Tableau(double[][] t){
  m = t.length-1;
  nrows = m+1;
  n = t[0].length-1;
  ncols = n+1;
  c = new double[n];
  for (int j = 0; j < n; j++){
    c[j] = t[m][j];
  }
  y = new double[nrows][ncols];
  for (int i = 0; i < nrows; i++){
    for (int j = 0; j < ncols; j++){
      y[i][j] = t[i][j];
    }
  }
  reduce();
}
/**
 * pivot operations to reduce the last row of the tableau, so that
 * coefficients of the basic variables are all equal to 0
 */
void reduce(){
```

```
    for (int i = 0; i < m; i++){
      double coeff = y[m][i];
      for (int j = 0; j < ncols; j++){
        y[m][j] = y[m][j]-y[i][j]*coeff;
      }
    }
  }
  /**
   * prints out the tableau
   */
  void print(){
    for (int i = 0; i < nrows; i++){
      for (int j = 0; j < n; j++){
        double d = y[i][j];
        if (Math.abs(d) < 1e-12)
          d = 0.;
        System.out.print(d+"\t");
      }
      double d = y[i][n];
      if (Math.abs(d) < 1e-12)
        d = 0.;
      System.out.println(d);
    }
    System.out.println("-------------");
  }
  /**
   * for testing
   * @param args
   */
  public static void main(String[] args){
    double[][] A = new double[2][3];
    double[] b = new double[2];
    A[0][0] = 2.;
    A[0][1] = 1.;
    A[0][2] = 2.;
    b[0] = 4.;
    A[1][0] = 3.;
    A[1][1] = 3.;
    A[1][2] = 1.;
    b[1] = 3.;
    AVSFormCons avsfc = new AVSFormCons(A, b);
    Tableau tab = new Tableau(avsfc);
    tab.print();
  }
}
```

```
import java.io.BufferedReader;
import java.io.BufferedWriter;
import java.io.FileReader;
import java.io.FileWriter;
import java.util.Random;
import java.util.StringTokenizer;
import java.util.Vector;
/**
 * general methods (mostly I/O) used by several classes
 */
public class Utils {
  /**
   * random selection of index i in F based on probabilities F[i+1]-F[i]
   * @param F cumulative probabilities
   * @param rand
   * @return random index i in F based on probabilities F[i+1]-F[i]
   */
  public static int getRandIndexInF(double[] F, Random rand){
    double d = rand.nextDouble()*0.99999999;
    int res = 0;
    if (d <= F[res]){
      return res;
    }
    else{
      while(d > F[res]){
        res++;
      }
      return res;
    }
  }
  /**
   * minimum
   * @param x
   * @return min of x
   */
  public static double getMin(double[] x){
    double res = x[0];
    for (int i = 0; i < x.length; i++){
      if (x[i] < res){
        res = x[i];
      }
    }
    return res;
  }
  /**
   * maximum
   * @param x
   * @return max of x
   */
  public static double getMax(double[] x){
    double res = x[0];
    for (int i = 0; i < x.length; i++){
      if (x[i] > res){
```

```
      res = x[i];
    }
  }
  return res;
}
/**
 * average
 * @param x
 * @return average of x
 */
public static double getAverage(double[] x){
  double res = 0.;
  for (int i = 0; i < x.length; i++){
      res += x[i];
  }
  res /= (double) x.length;
  return res;
}
/**
 * standard deviation
 * @param x
 * @return standard deviation of x
 */
public static double getSD(double[] x){
  double res = 0.;
  double av = getAverage(x);
  for (int i = 0; i < x.length; i++){
      res += (x[i]-av)*(x[i]-av);
  }
  res /= (double) (x.length-1);
  res = Math.sqrt(res);
  return res;
}
/**
 * loads an ASCII file into a vector, line by line
 * @param file  ASCII file, tab-delimited
 * @return Vector representation (one line in each element) of file
 */
public static Vector<String> loadFileNoheader(String file){
  Vector<String> res = new Vector<String>();
  try{
    FileReader fr = new FileReader(file);
    BufferedReader br = new BufferedReader(fr);
    String line = br.readLine();
    while((line = br.readLine()) != null){
      StringTokenizer st = new StringTokenizer(line, "\t");
      if (st.countTokens() >= 2){
        res.add(line);
      }
    }
    br.close();
    fr.close();
  }
  catch(Exception ex){
    ex.printStackTrace();
```

```
    }
    return res;
  }
  /**
   * saves each element of Vector v into a line of ASCII file 'file'
   * @param v contains file lines, each one being tab-delimited
   * @param file output file
   */
  public static void saveFile(Vector<String> v, String file){
    try{
      FileWriter fw = new FileWriter(file);
      BufferedWriter bw = new BufferedWriter(fw);
      for (int i = 0; i < v.size(); i++){
        bw.write(v.elementAt(i));
        bw.newLine();
      }
      bw.close();
      fw.close();
    }
    catch(Exception ex){
      ex.printStackTrace();
    }
  }
}
```
